# An Adaptable Porous Metal–Organic Framework for Structural Visualization of Beyond Rule of 5 Molecules on the Microgram Scale

**DOI:** 10.1021/jacs.6c08312

**Published:** 2026-08-04

**Authors:** Taichi Baba, Yu Tagami, Natsumi Watanabe, Bun Chan, Tomoki Nakagawa, Yiying Zhu, Arihiro Iwasaki, Pavel M. Usov, Yuki Wada, Masaki Kawano

**Affiliations:** † Department of Chemistry, School of Science, 693022 Institute of Science Tokyo, Meguro-ku, Tokyo 152-8550, Japan; ‡ Department of Applied Chemistry, Faculty of Science and Engineering, 84021 Chuo University, Bunkyo-ku, Tokyo 112-8551, Japan; § Graduate School of Engineering, 12961 Nagasaki University, Nagasaki-shi, Nagasaki 852-8521, Japan; ∥ RIKEN Center for Computational Science, Kobe-shi, Hyogo 650-0047, Japan; ⊥ TEKMOF Co., Ltd., Minato-ku, Tokyo 108-0023, Japan

## Abstract

Beyond rule of 5 (bRo5) pharmaceutical compounds represent major structural challenges due to their large size and complexity. Here, we show that a metal–organic framework, APF-40, enables encapsulation and structural visualization of bRo5 molecules. It comprises a new benzoate-substituted hexaazaphenalene ligand and Zn_8_ clusters forming a doubly interpenetrated framework. The dynamic movement of the two nets facilitates adaptive behavior, efficiently immobilizing molecules along with their solvation spheres. Structures of 12 guests, including eight bRo5 compounds with subtly differing substituent modifications— methyl-to-ethyl, methoxy-to-hydroxy, and alkane-to-alkene—were precisely elucidated. Significantly, one compound, isolated from a commercially available sample, was identified as a novel ivermectin derivative. This work demonstrates μg-order structure determination of molecules larger than 500 Da, offering new possibilities for characterizing compounds previously intractable for established analytical techniques.

## Graphical abstract

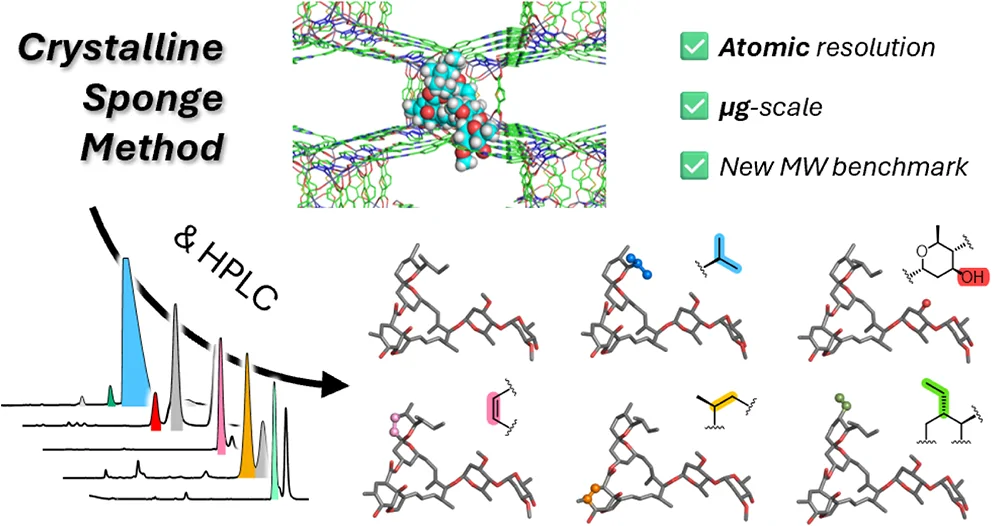

## Introduction

Beyond rule of 5 (bRo5) molecular drugs have emerged as a transformative paradigm in pharmaceutical research due to their unique therapeutic potential.^1^ These molecules reside outside the Lipinski’s rule of five chemical space^2^ while retaining drug-like properties. Specifically, their molecular weights tend to exceed 500 Da combined with other rule violations (>5 hydrogen bond donors, >10 acceptors or logP > 5). However, conventional analytical methods suffer inherent limitations when applied to molecules in this size range. Their high degree of molecular complexity makes it difficult to interpret nuclear magnetic resonance (NMR) spectra. Although single-crystal X-ray diffraction (SCXRD) is the most promising method for molecular structure determination that can provide absolute configuration, crystallization of bRo5 molecules is a nontrivial task with no upper bound on the time and effort it might take to screen and optimize the crystallization conditions. In the past decade, several diffraction-based methods have been developed to analyze bRo5 drug molecules. One of the promising methods, microcrystal electron diffraction (MicroED),^3–5^ has less stringent requirements for the crystal size and quality, however the target molecules still need to be crystallized and ordered to diffract well enough to analyze their structures. Another recent development is cocrystallization techniques,^6–12^ which can significantly reduce the crystallization effort by introducing molecules that readily form cocrystals with the broad range of compounds. The earliest work in this field dates to the 1990s, when triphenylphosphine oxide^6^ or porphyrin^7^ derivatives were employed to cocrystallize a series of aromatic compounds. Since then, a variety of cocrystallization agents have been developed with an expanded range of capabilities, including selective isolation of specific components from mixtures. In this approach, formation of a complex between the target compound and the cocrystallization agent is the key process; however, increasing the size of participating molecules renders it entropically unfavorable. As a result, reports of successful application of this method to molecules exceeding 500 Da remain scarce. Recently, a palladium-based discrete cage complex termed the “second-generation crystalline sponge” was introduced, extending structural analysis to compounds with molecular weights exceeding 1000 Da and falling within the bRo5 chemical space.^12,13^ While this material can also be applied as a crystalline sponge (*vide infra*) owing to its ability to assemble porous crystals, the majority of reported examples followed the cocrystallization procedure. In addition, structural analysis of the microgram sample amounts has been demonstrated through the use of microvolume capillaries.^14^ However, even with the usage of the “second-generation crystalline sponge”, the number of successful examples of structural analysis of molecules exceeding 500 Da remains limited. At present, there are no cocrystallization agents available to routinely carry out structure determination in this chemical space.

Alternatively, preformed single-crystal matrices, such as metal–organic frameworks (MOFs), have been developed to overcome traditional crystallization requirements by incorporating and aligning guest molecules into their ordered channels. In this approach, defined as the “crystalline sponge method”,^15,16^ the single-crystal nature of the supporting matrix is crucial, as it enables direct structure determination of included guests through X-ray crystallographic analysis. This method benefits from requiring only a small number of preformed crystals to be soaked in a solution of the target compound followed by SCXRD. As a result, the required sample amount could be potentially reduced to the nanogram level^17,18^ representing a significant advantage over the cocrystallization methods, which typically operate on the microgram scale.^14^ Unfortunately, the current iteration of crystalline sponges also encounters molecular weight limitations at the threshold of bRo5 chemical space. The diffusion of molecules throughout the crystal is restricted by channel dimensions, specifically the sizes of minimal apertures. As a result, the largest guest successfully analyzed using this technique to date only reached 678 Da or 48 non-hydrogen atoms^12,19–34^ (Figure 1 and Table S1). There is still only a handful of examples exceeding 500 Da, which is sorely inadequate for covering the bRo5 space. While large-pore MOFs (>2 nm) could resolve the diffusion limitations, they face other potential drawbacks. They tend to crystallize in highly symmetric space groups, including tetragonal, hexagonal or cubic systems,^35^ leading to pseudosymmetry problems that severely complicate the assignment of electron densities.^17^ Moreover, their large channels offer very few interaction points to the encapsulated guests increasing the likelihood of severe disorder. This behavior highlights the difficulty of balancing the pore size and interactivity inside frameworks to accommodate increasingly larger molecules.^36^ Among the currently reported MOF structures, hardly any simultaneously satisfy these stringent criteria presenting a major opportunity for development of new materials targeting the crystalline sponge application.

**Figure 1:**
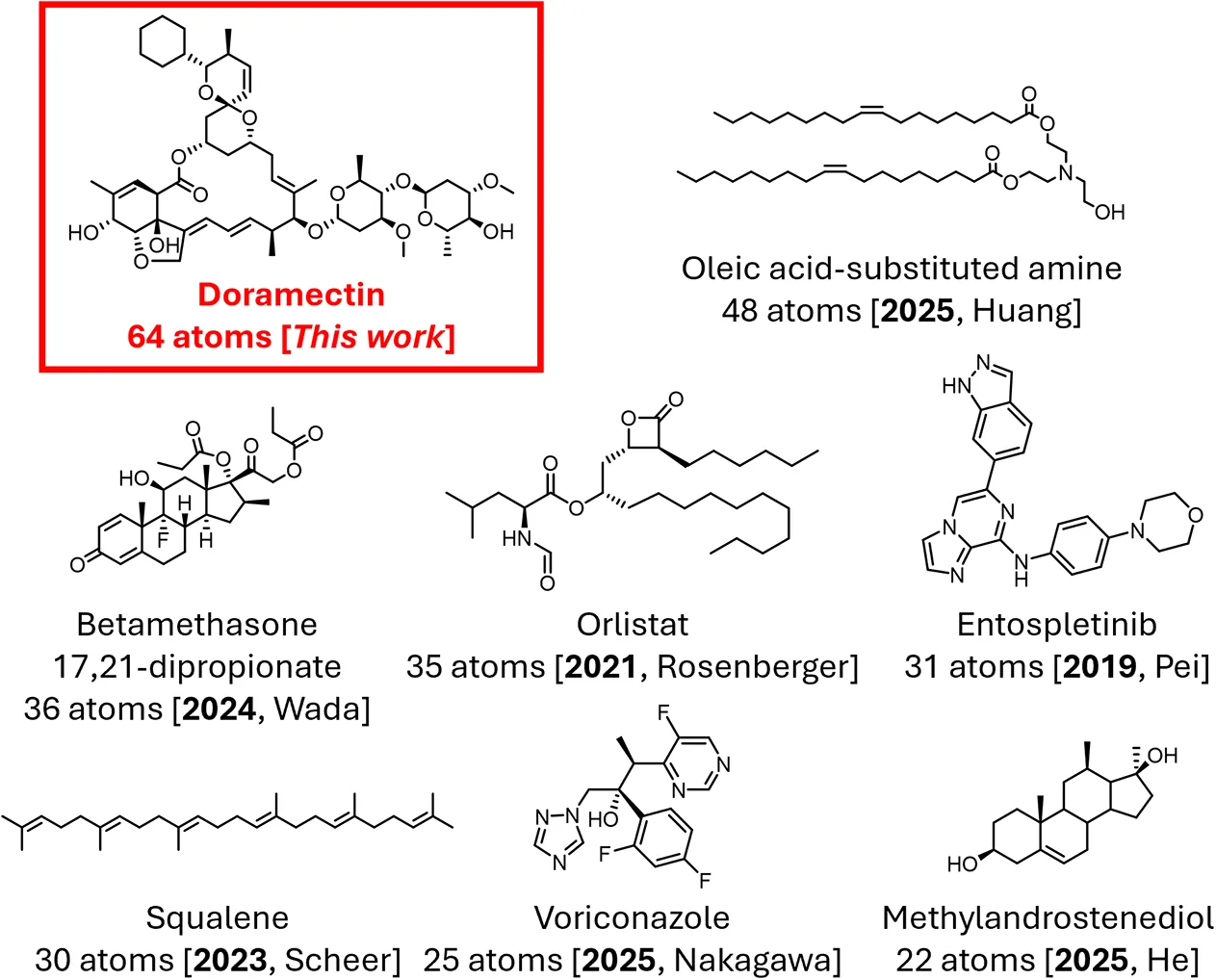
Largest guests analyzed by encapsulation into the representative crystalline sponge materials. The molecular sizes are ordered by the number of non-hydrogen atoms.

## Results and Discussion

### MOF Design for the Analysis of Compounds Larger Than 500 Da

Previously, we described a MOF series for guest encapsulation and structure determination.^20,24^ These systems incorporate pyridyl-based ligands based on hexaazaphenalene (HAP) core, where all α-positions of the parent phenalene skeleton are substituted with six nitrogen atoms. This modification lowers the orbital energy and enhances N–H acidity, stabilizing the delocalized anionic charge.^37,38^ Additionally, the nitrogen atoms can readily form hydrogen bonds with other species further enhancing the ligand interactivity.^39–43^ Search of the MOF literature identified a series of structures based on the octanuclear Zn^2+^ cluster,^44^ which contains 2,5-thiophenedicarboxylate (TDC) and 1,2,3-triazolate (TAZ) ligands, and can support large pore space when combined with *C*3 symmetric carboxylate ligands. According to these design criteria, three benzoate coordinating groups were introduced at β-positions of the HAP core to create H_4_TCHAP following the outlined synthetic route (Scheme 1).

**Scheme 1:**
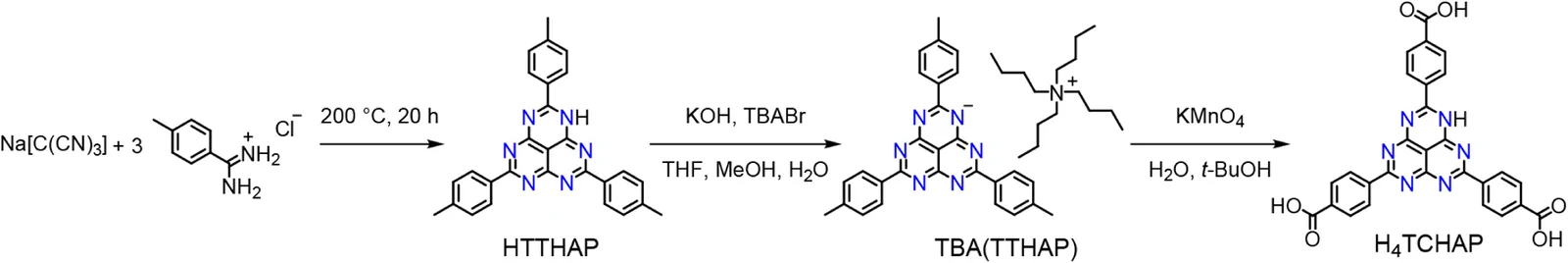
Synthesis of H_4_TCHAP^a^

a H_4_TCHAP synthesis using condensation, cation exchange and oxidation reactions.

The novel MOF, denoted as APF-40 (APF = adaptable porous framework), was crystallized under solvothermal conditions from a mixture of H_4_TCHAP, ZnI_2_, HTAZ, H_2_TDC and acetic acid modulator in *N,N*-dimethylformamide (DMF), as pale-yellow plate crystals with sufficient size for SCXRD (Figure 2A). The resultant framework displayed the molecular formula of (NH_2_Me_2_)_2_[Zn_8_(TDC)_3_(TAZ)_6_(H_2_O)_2_(HTCHAP)_2_], with charge-balancing dimethylammonium cations (DMA^+^) generated from decomposition of DMF. APF-40 incorporated the target octanuclear clusters interconnected by HTCHAP^3–^ linkers assembling into a doubly interpenetrated 3D network, which crystallized in the monoclinic *C*2/*m* space group. The structure featured large channels (17.4 × 12.5 Å^2^) along the *c*-axis, surpassing other MOFs used for the crystalline sponge method (Table S1), which coexisted with narrower channels occurring between neighboring Zn_8_ clusters from the two nets. They were filled with DMA^+^ and water molecules providing auxiliary hydrogen bonding that could adapt in response to guest encapsulation (Figure 2B to D). The two interpenetrated nets were joined together through hydrogen bonding between coordinated water molecules (Figure 2E) enhancing MOF structural integrity and allowing it to maintain pore accessibility.

**Figure 2:**
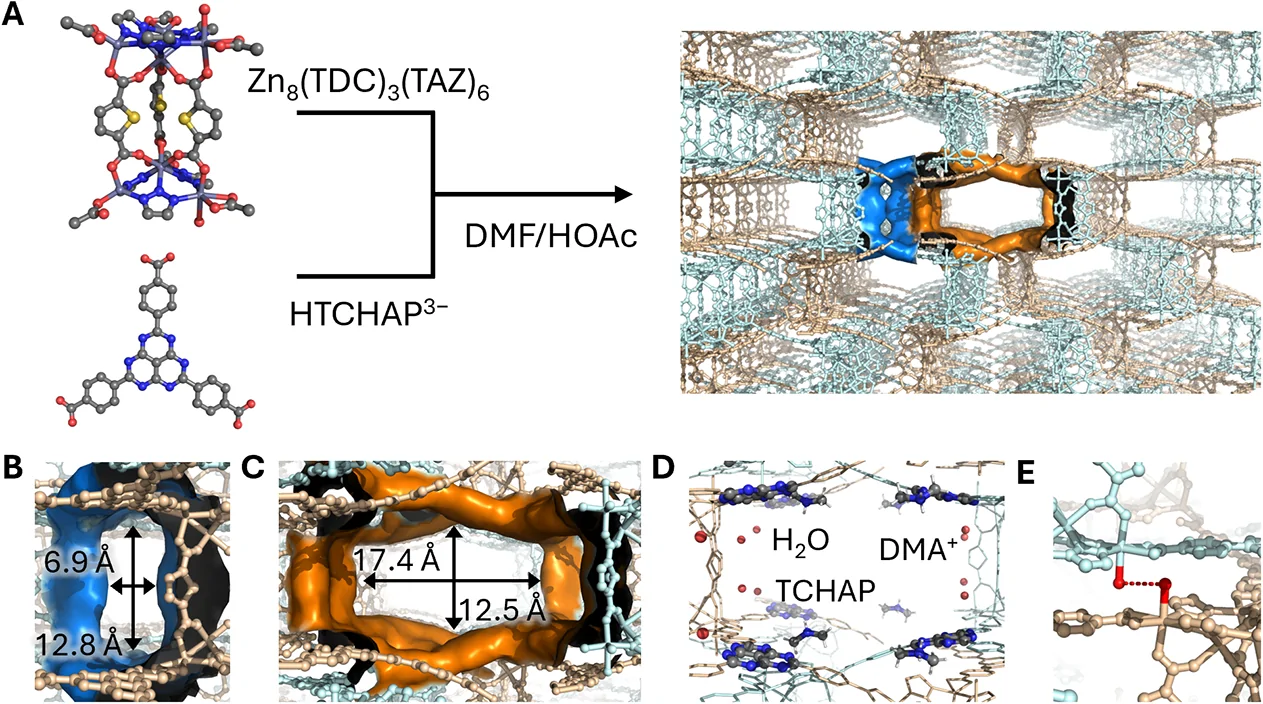
Synthetic scheme and structural details of APF-40. (A) Assembly of the APF-40 structure, which is comprised of octanuclear Zn_8_(TDC)_3_(TAZ)_6_(H_2_O)_2_ clusters connected by HTCHAP^3–^ ligands featuring a doubly interpenetrated framework with narrow (*blue surface*) and wide channels (*orange surface*). (B) Structure of narrow channel, (C) structure of wide channel and (D) interactive sites. (E) Doubly interpenetrated framework is stabilized through hydrogen bonds between two coordinated water molecules. Atom coloring scheme: Zn–purple, C–gray, H–white, O–red, N–blue and S–yellow.

### Screening Compounds for Encapsulation into APF-40

In host–guest chemistry, size and shape complementarity between hosts and guests are key factors governing the inclusion process.^45^ To assess guest immobilization and identify the optimal size range, we selected seven pharmaceutical compounds divided into three groups: small steroids (spironolactone, dexamethasone and betamethasone), flexible long-chain compounds exceeding 500 Da (stearyl glycyrrhetinate and fulvestrant) and medium-sized bRo5 avermectin derivatives (Figure 3). All guests were successfully encapsulated without extensive condition optimization (Table S2), demonstrating high versatility of APF-40. Notably, we determined the structure of doramectin (899.13 Da), substantially expanding the molecular size range reachable by the MOF-based structural analysis.

**Figure 3:**
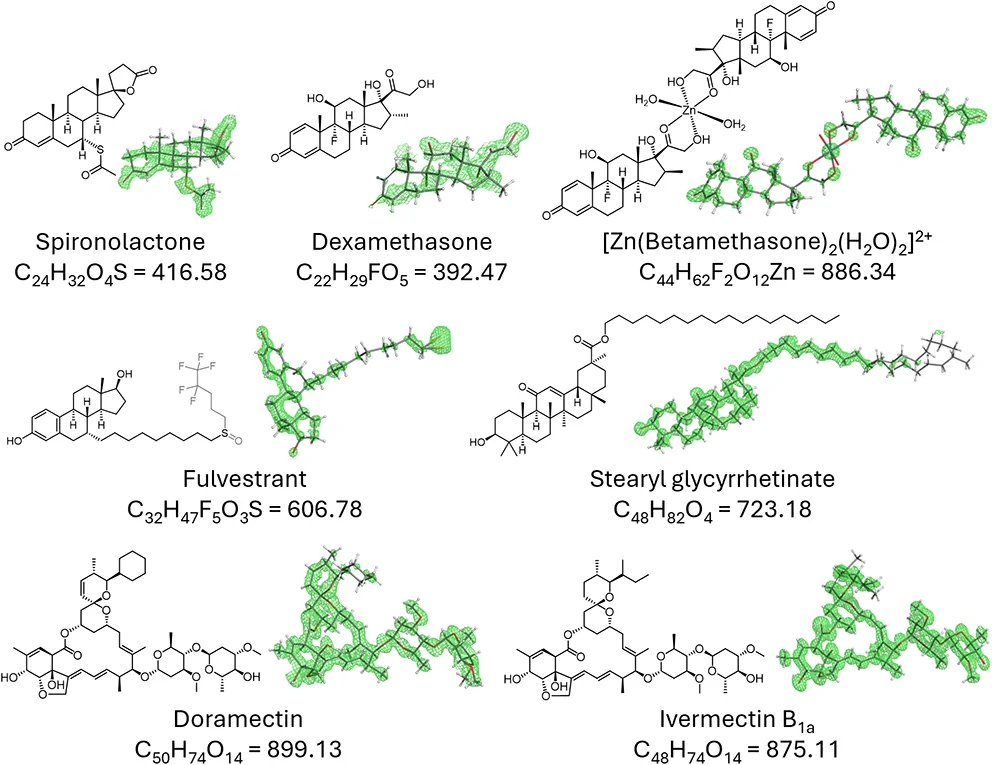
Molecular structures and corresponding electron density maps (2Fo–Fc) of seven guests determined in APF-40. The formulas and molecular weights (Da) are shown. Betamethasone is encapsulated as bis-betamethasone zinc­(II) complex. Structure of fulvestrant is only partially resolved due to disorder of the fluoroalkyl tail.

### Structural Elucidation of Small Molecules in APF-40

Refinement of APF-40 structures containing small steroids revealed highly variable behavior despite their molecular similarity. Soaking framework crystals in spironolactone solution induced a space group transformation from *C*2/*m* to the noncentrosymmetric *C*2 (Flack parameter 0.124(6)), with three crystallographic sites identified showing occupancies of 64.4(9)%, 42.5(10)% and 25% (Figure S13). Spironolactone molecules assembled into a dimeric structure by forming hydrogen bonds through one water molecule between the two highest occupancy sites (Figure 4A). On the other hand, the third site with the lowest occupancy existed in the monomeric form. Both the monomer and the dimer resided inside the narrow channel, which widened upon spironolactone inclusion demonstrating an adaptive framework dynamics (Figure S14). For dexamethasone, only one site could be resolved inside APF-40 with 30% occupancy (Flack parameter 0.410(6)) due to a limited number of hydrogen bonding interactions through hydroxy groups and size mismatch relative to the pore dimensions (Figure 4B and Figure S15). In contrast, its diastereomer, betamethasone, was encapsulated with the occupancy of 68.5(8)% generating [Zn­(betamethasone)_2_(H_2_O)_2_]^2+^ complex (Figure S16). The dimer was supported by eight hydrogen bonds with the framework backbone (Figure 4C) significantly restricting its movement and reducing the Flack parameter of the resultant structure to 0.151(8). Betamethasone and dexamethasone differ only in stereochemistry of the C16 methyl group, yet this subtle change drastically altered their encapsulation behavior (Figure 3). Overall, betamethasone and spironolactone formed dimeric assemblies inside the pore of APF-40—a zinc-bridged complex and water-mediated hydrogen-bonded dimers, respectively, while dexamethasone remained in its monomeric form. This dimerization process compensated for suboptimal matching of guest sizes compared to significantly larger framework pores, allowing to achieve sufficient occupancy and high structure refinement accuracy, which confirmed the suitability of APF-40 for the analysis of molecules larger than 500 Da.

**Figure 4:**
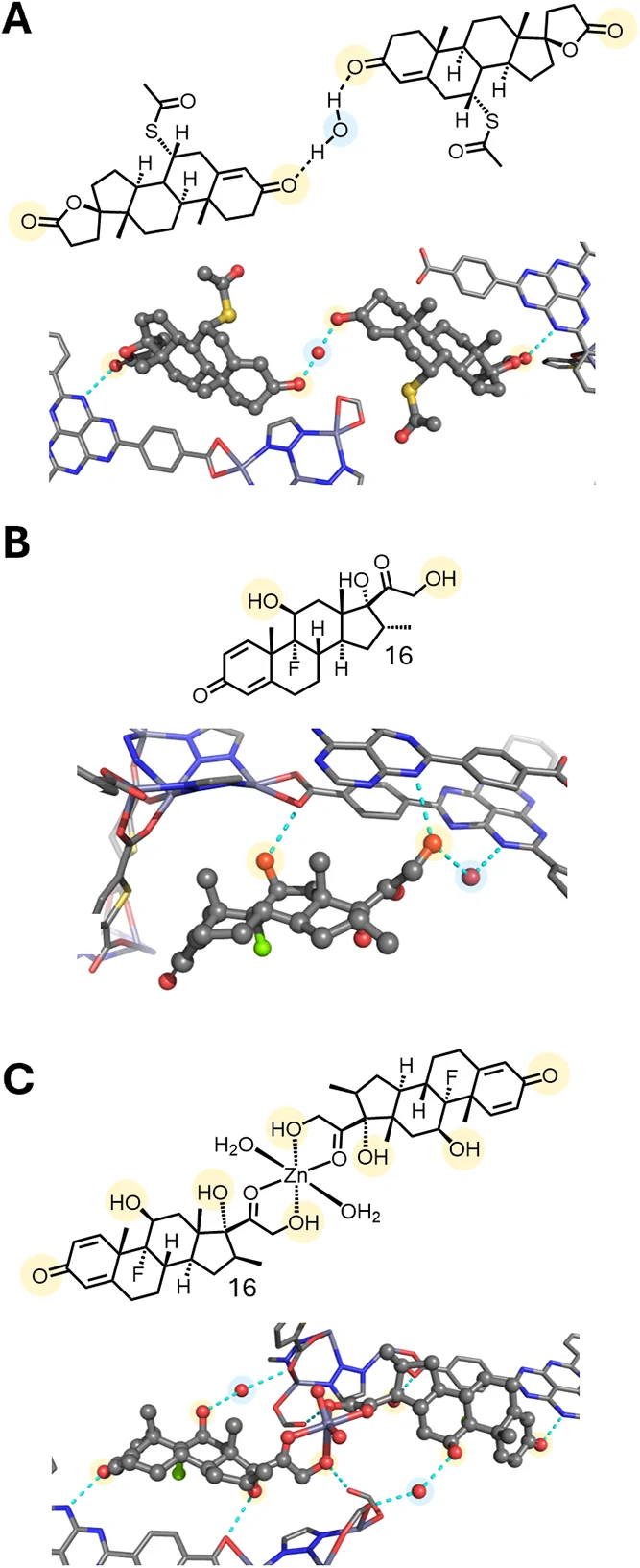
Structures and interactions of analyzed steroid guests. (A) The dimeric structures of water-mediated hydrogen-bonded spironolactone molecules visualized in APF-40 shown in ball-and-stick models. (B) The monomeric structure of dexamethasone. (C) The dimeric structures of bis-betamethasone complex. Oxygen atoms of hydrogen-bonding sites in guests and water molecules are highlighted in yellow and light blue colors, respectively. Cyan dash lines represent hydrogen bond contacts. Atom coloring scheme: Zn–purple, C–gray, O–red, N–blue, S–yellow and F–light green.

### Structural Elucidation of Compounds Containing Flexible Moieties in APF-40

Long-chain alkyl groups are ubiquitous motifs in natural products and pharmaceuticals; however, their structural characterization remains fundamentally challenging due to conformational flexibility and weak intermolecular interactions impeding their crystallization. We postulated that the interactive channels of APF-40 could spatially organize and order such flexible chains. To evaluate this hypothesis, two pharmaceutical compounds whose crystal structures have not been reported in the CCDC database, stearyl glycyrrhetinate and fulvestrant, were tested for encapsulation into APF-40.

Stearyl glycyrrhetinate (C_48_H_82_O_4_, 723.18 Da) is an anti-inflammatory medicine containing C_18_ alkyl group, whose molecular motion is difficult to restrict, which precludes its ordered arrangement. SCXRD analysis of the encapsulated crystal of APF-40 revealed an unprecedented binding mode with seven independent crystallographic sites exhibiting varying occupancies: A and M at 100%; and B, C, D, F and G ranging from 37.5(3)% to 62.5(3)% (Figure S17). The guests in the fully occupied sites assembled into supramolecular dimers supported by dispersion contacts between stearyl groups and hydrogen bonds mediated by four water molecules and two DMA^+^ cations (Figure 5A). This assembly was further immobilized by hydrogen bonding with the framework. Despite the conformational flexibility of stearyl groups, requiring two-component disorder modeling at sites A and M, intermolecular dispersion forces provided sufficient stabilization limiting their movement within the pore. In contrast, the lower-occupancy sites exhibited a monomeric character forming hydrogen bonds only with the backbone of APF-40, comparable to the structure of dexamethasone. Remarkably, despite the disorder of C_18_ chains, well-defined electron density was observed enabling reliable structure determination with a Flack parameter of 0.046(3).

**Figure 5:**
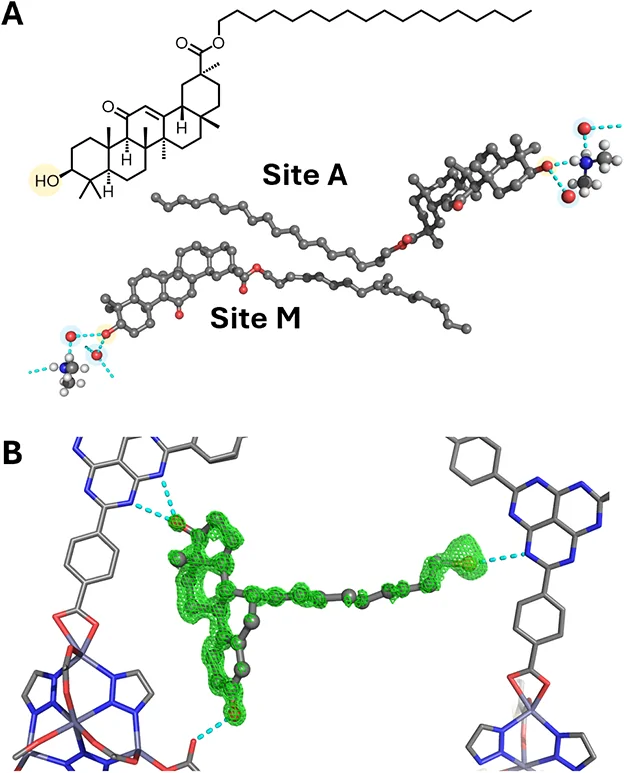
Structures and crystallographic features of encapsulated guest compounds containing flexible moieties. (A) Chemical structure of stearyl glycyrrhetinate and ball-and-stick model of dimeric stearyl glycyrrhetinate immobilized with two DMA^+^ and four water molecules in sites A and M. (B) Ball-and-stick model of fulvestrant immobilized at three hydrogen bonding sites showing electron density map (2Fo–Fc) (map threshold is 1.30 e/Å^3^). Oxygen atoms of hydrogen-bonding sites in stearyl glycyrrhetinate and water molecules are highlighted in yellow and light blue colors, respectively. Cyan dash lines represent hydrogen bond contacts. Atom coloring scheme: Zn–purple, C–gray, H–white, O–red, N–blue and S–yellow.

Fulvestrant (C_32_H_47_F_5_O_3_S, 606.78 Da) is a selective estrogen receptor degrader used for breast cancer therapy containing C_9_ alkyl and -(CH_2_)_3_CF_2_CF_3_ fluoroalkyl chains linked via a sulfinyl group. SCXRD analysis of the fulvestrant single crystal revealed that both of the flexible moieties possessed two disordered pairs with occupancies of 44.4(7)% to 55.6(7)% and 27.1(12)% to 72.9(12)% for C_9_ alkyl and fluoroalkyl chains, respectively (Figure S19). On the other hand, encapsulation of fulvestrant into APF-40 provided crystallographically precise electron density for the steroid core and C_9_ alkyl chain (Figure S18). The guest was immobilized through three hydrogen bonds at the periphery of the molecule: two hydroxy groups in the steroid skeleton and sulfur atom in the alkyl tail, suppressing the intrinsic disorder of flexible groups (Figure 5B). As a result, the fulvestrant site exhibited 88.2(10)% occupancy and had accurately assigned stereochemistry (Flack parameter 0.031(4)). However, electron densities for the expected sulfinyl and fluoroalkyl groups were not observed, suggesting either severe disorder or degradation during encapsulation. Although fulvestrant can be susceptible to bond cleavage,^46^ mass spectrometry (MS) analysis of the guests extracted from APF-40 did not detect significant quantities of degradation products, therefore confirming that it was encapsulated intact (Figure S20). Then, the observed weak electron densities were caused by conformational disorder rather than missing fluoroalkyl groups.

### Structural Elucidation of bRo5 Compounds in APF-40

Having demonstrated the ability of APF-40 to structurally characterize flexible molecules exceeding 500 Da, next, our attention was turned to the bRo5 chemical space. The avermectin derivatives hold a particular significance: ivermectin is a synthetic derivative of avermectin B_1_, which improved antiparasitic therapy and earned William C. Campbell and Satoshi O̅mura the 2015 Nobel Prize in Physiology or Medicine.^47,48^ Two commercially available derivatives, doramectin (C_50_H_74_O_14_, 899.13 Da) and ivermectin B_1a_ (C_48_H_74_O_14_, 875.11 Da) were selected.

Doramectin is a 16-member macrocyclic lactone substituted with the hydrophobic cyclohexyl group and α-l-oleandrose based disaccharide unit at C25 and C13, respectively, highlighting its amphiphilicity. The combination of these structural features in one molecule complicates the structural characterization. SCXRD analysis after encapsulation into APF-40 revealed doramectin at two crystallographically independent sites with occupancies of 80.6(8)% and 35.8(8)% (Figure S21). Notably, the guests were positioned between unit cells of the original lattice, which broke the translational symmetry. Therefore, data indexing and integration were performed in a doubled *c*-axis supercell. The higher occupancy site Z arranged into an unexpected supramolecular assembly. Doramectin molecules were captured within the narrow channel as a dimer mediated by hydrogen bonds from the hydroxyl group on C7 and the ether group on the outer saccharide heterocycle (Figure 6A). The two interpenetrated nets of APF-40 underwent a dynamic shift expanding the cavity to optimally fit the doramectin dimer (Figure S22). This adaptive reorganization enabled the guest to form six hydrogen bonds facilitating its capture and enhancing the occupancy of site Z (Figure 6B). As a result, the diffraction resolution was sufficient to reliably model all atoms with minimal restraints except for the flexible cyclohexyl group, fully resolving the structure with correct stereochemistry of all 19 chiral centers (Figure S62). At the lower-occupancy site Y, doramectin adopted a different interaction mode supported exclusively by hydrogen bonds with HAP cores. These results emphasized the multimodal binding and recognition abilities of the APF-40 pore.

**Figure 6:**
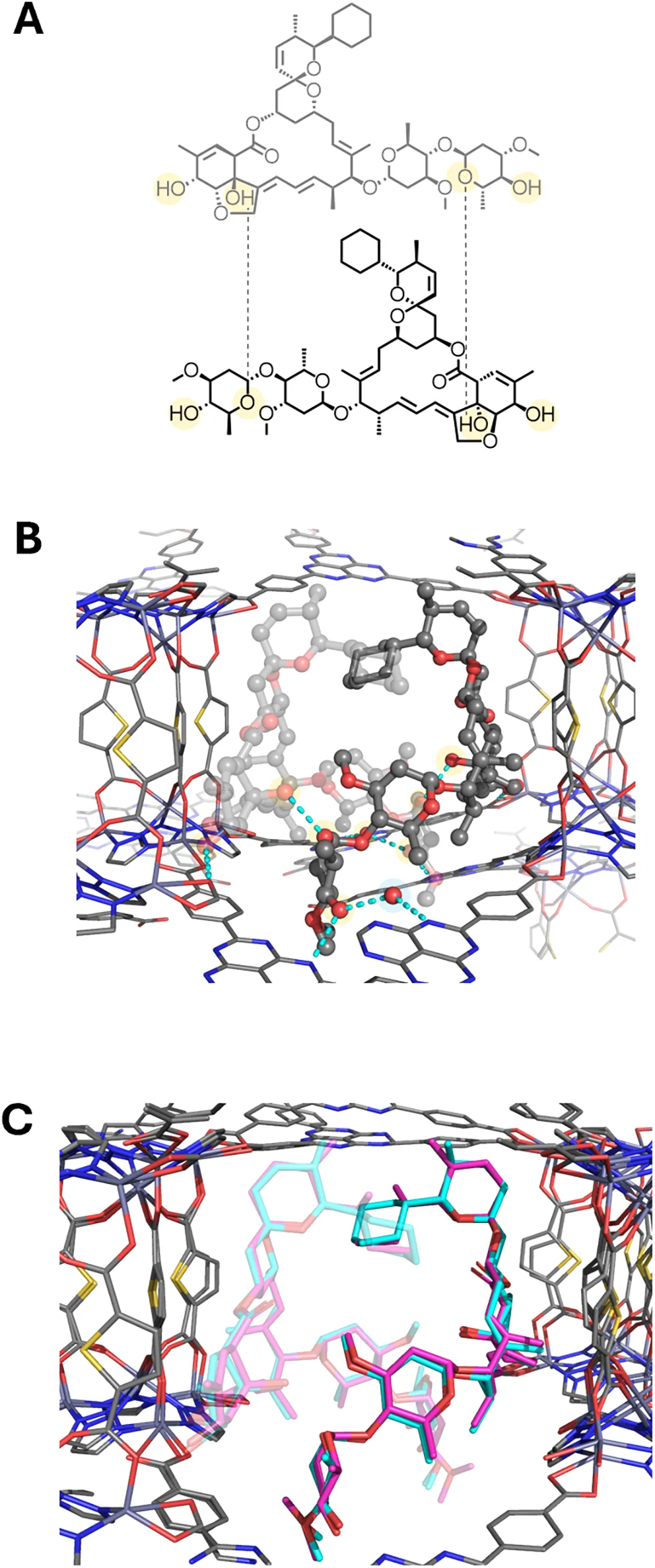
Structures and crystallographic features of encapsulated bRo5 compounds. (A) Interaction manner and (B) ball-and-stick model of dimeric doramectin encapsulated in site Z. (C) Stick model of ivermectin B_1a_ (*magenta*) overlaid over doramectin molecules exhibiting the same interaction types (*cyan*). Oxygen atoms of hydrogen-bonding sites in guests and water molecules are highlighted in yellow and light blue colors, respectively. Cyan dash lines represent hydrogen bond contacts. Atom coloring scheme: Zn–purple, C–gray, O–red, N–blue and S–yellow.

Next, APF-40 was applied for the analysis of ivermectin B_1a_, which differs from doramectin by the *sec*-butyl substituent on the C25 atom and the single C22–C23 bond, thus testing the influence of subtle structural variations on the guest encapsulation behavior. Crystallographic analysis revealed the presence of two binding sites with occupancies of 100% and 42.7(8)% (Figure S23). In the fully occupied site Z, ivermectin B_1a_ molecules assembled into hydrogen-bonded guest–guest dimers, occupying nearly identical locations in the pore to those of doramectin (Figure 6C). This highly reproducible guest immobilization phenomenon suggested that the binding site could effectively complement the geometry of avermectin derivatives promoting the thermodynamic stabilization of the resultant host–guest assemblies. The lower-occupancy site Y resided in the large channel of APF-40 and was supported by two hydrogen bonds connected between ether and hydroxyl group in spiroketal and benzofuran moieties to the HAP core of framework rather than guest–guest interaction contacts. These structures represent a landmark achievement since both ivermectin B_1a_ and doramectin, with their molecular weights of 875.11 and 899.13 Da, respectively, are considerably larger than all other molecules previously characterized by the crystalline sponge method (Table S1). It was demonstrated for the first time that diffusion-mediated immobilization inside MOF matrices could successfully handle >700 Da compounds leading to their structural visualization.

### Analysis of Trace Components in the Commercial Ivermectin B_1a_ Sample

Closer scrutiny of the ivermectin B_1a_ structure model revealed unusually large atomic displacement parameters (ADPs) around the spiroketal group even in the fully occupied site Z (Figure 7A). Specifically, low-contour electron density maps contained sharp, well-resolved features indicative of static rather than dynamic disorder (Figure 7B). Given that the purity of commercial sample was only 94.9%, we hypothesized that the disordered region could have originated from different overlapping molecules residing in the same space. To test this hypothesis, we performed HPLC separation and conducted integrated structural analysis of minor components by combining crystalline sponge encapsulation, NMR spectroscopy and mass spectrometry (MS).^49^ HPLC separations were performed using methanol or acetonitrile mixtures with water as mobile phases (Figures S24 to S29).

**Figure 7:**
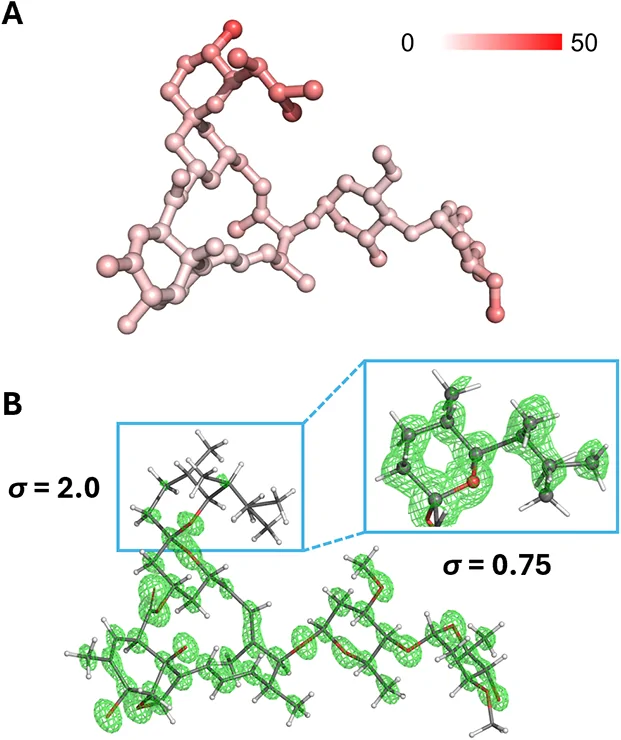
Structure of commercial ivermectin B_1a_ highlighting disorder around the spiroketal motif. (A) Isotropic atomic displacement parameters of ivermectin B_1a_ before purification. (B) electron density maps (2Fo–Fc) of ivermectin B_1a_ encapsulated in APF-40.

Fractions A and B were identified as ivermectin B_1a_ and B_1b_ (recovery = 90% and 0.6%, respectively) by ^1^H NMR and MS. The spectroscopic data for fraction A matched with reported ivermectin B_1a_.^50^ Whereas for fraction B, disappearance of a triplet signal corresponding to methyl protons in the *sec*-butyl group at 0.94 ppm and a decrease (Δ*m*/*z* = −14.026) in the molecular weight implied demethylation of the *sec*-butyl group to the corresponding *iso-*propyl (Figure 8A and B, and Figures S30 to S34). Moreover, peaks 1 and 3 detected in the vicinity of the main fraction A were assumed to contain closely related derivatives (Figure S24). Through sequential purification, we successfully obtained four additional fractions C, D, E and F in minute amounts (0.1–0.4 mg, recovery = 0.09–0.4%) with purity confirmed by NMR (Figure 8B, and Tables S3 and S4). While these sample quantities were insufficient for 2D NMR analysis, application of the crystalline sponge method employing APF-40 afforded adequate molecular structure including absolute configuration.

**Figure 8:**
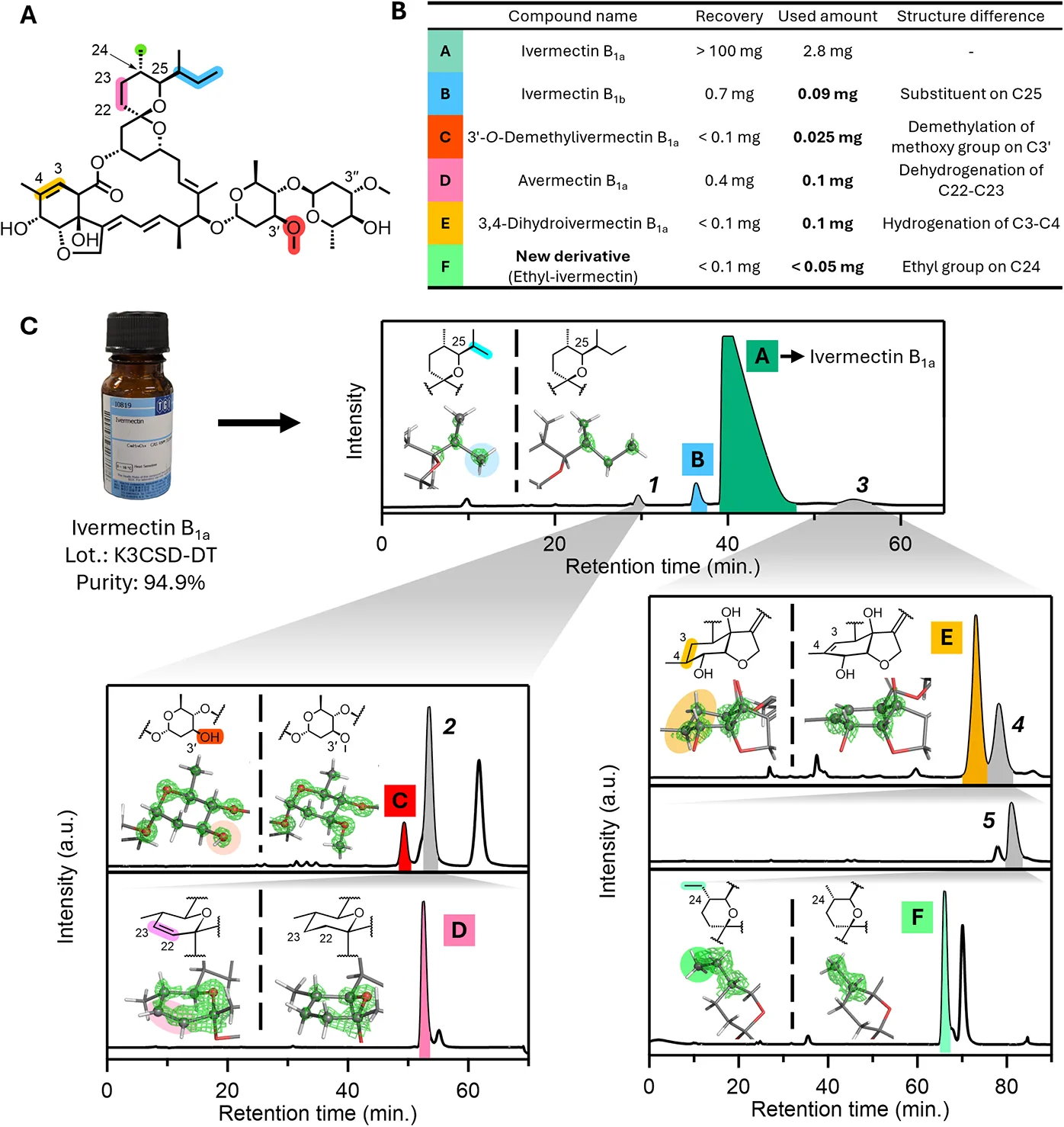
Crystal structures of six ivermectin derivatives elucidated by the crystalline sponge method coupled with HPLC separation. (A) Molecular structure of ivermectin B_1a_ highlighting structural differences relative to ivermectin B_1b_ (*light blue*), 3′-*O*-demethylivermectin B_1a_ (*red*), avermectin B_1a_ (*pink*), 3,4-dihydroivermectin B_1a_ (*orange*) and ethyl-ivermectin (*light green*). (B) Ivermectin derivatives and their corresponding fractions with HPLC separation recoveries, sample amounts used for the encapsulation into APF-40 and structural deviations from ivermectin B_1a_. (C) Scheme of HPLC separation starting from commercial sample to obtain six pure fractions. Molecular structures with electron density maps (2Fo–Fc) of derivatives (*left*) and ivermectin B_1a_ (*right*) are shown as insets inside each HPLC chart.

In fraction C, a decrease in molecular weight was observed in the MS (Δ*m*/*z* = −14.024) (Figure S35) attributed to a CH_2_ unit. At the same time, the ^1^H NMR signals corresponding to methoxy protons at C3′ and C3″ positions of the disaccharide unit integrated to 3H instead of the expected 6H, suggesting a demethylated derivative (Figure 9A and Figure S37). However, the presence of multiple methoxy groups with similar chemical environments, combined with considerations of the biosynthetic pathway in the AveBVII mutant strain of *S. avermitilis*,^51^ indicated that either demethylated compound could exist. It was therefore difficult to correctly assign the modified group from the ^1^H NMR and MS data alone. Crystallographic structural analysis using APF-40 clearly revealed lack of electron density around the C3′a methyl group, providing evidence for the demethylation of α-l-oleandrose attached to the macrocycle (Figure 9B and Figure S53).

**Figure 9:**
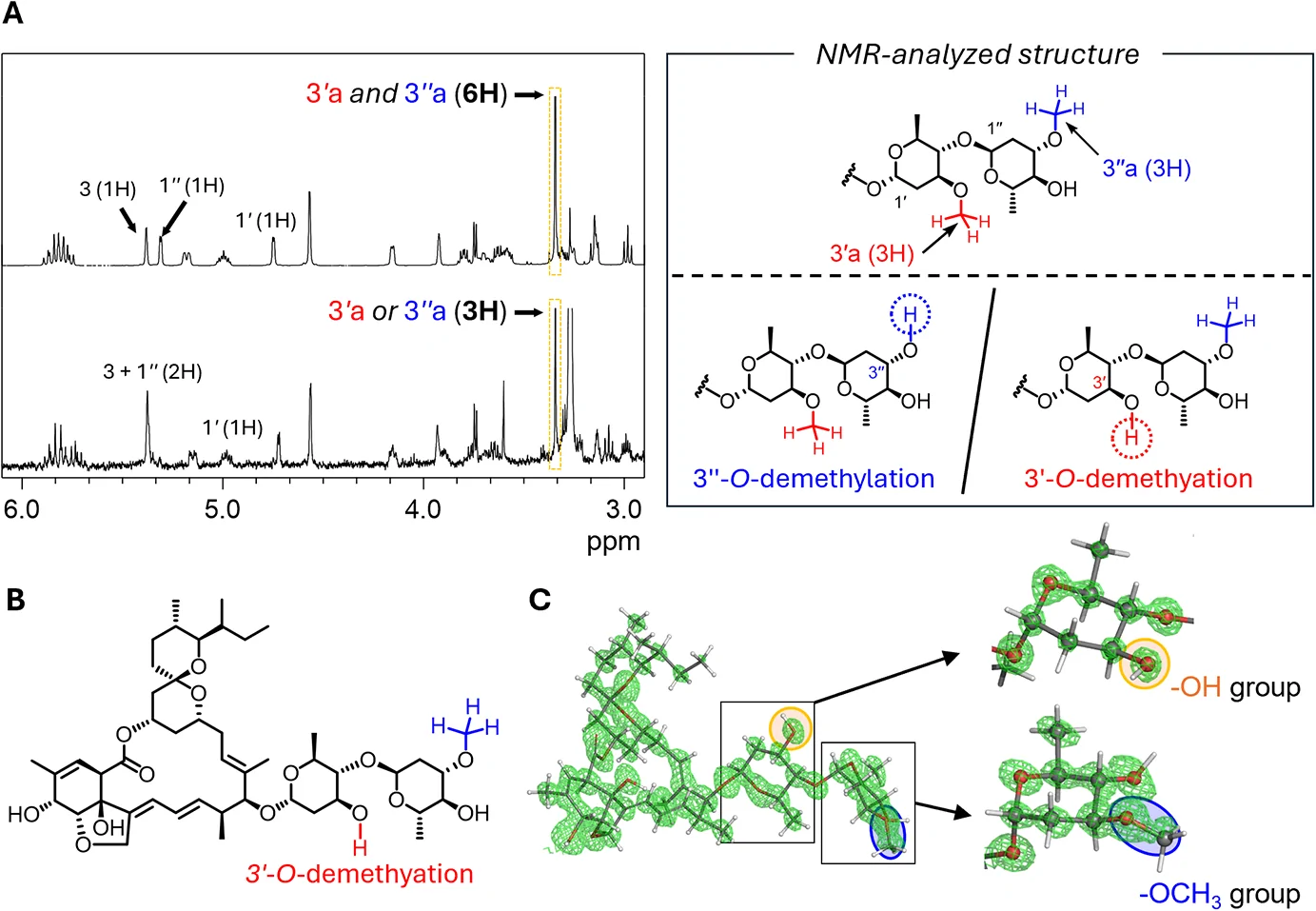
Analysis of the ivermectin derivative from fraction C. (A) ^1^H NMR spectra of ivermectin B_1a_ (*top*) and fraction C (*bottom*) and their corresponding molecular structures from the ^1^H NMR spectra assignment (*right*). Both possible demethylated compounds are shown. (B) The molecular structure of fraction C determined by SCXRD inside APF-40 showing the correct 3′-*O*-demethylation. (C) Crystal structure of fraction C with 2Fo–Fc electron density map (map threshold is 1.02 e/Å^3^) and the 2Fo–Fc electron density maps of each saccharide moiety (map thresholds are 1.0 e/Å^3^ and 1.3 e/Å^3^ for the demethylated α-l-oleandrose and the terminal α-l-oleandrose, respectively).

Reliable structural determination of each fraction was achieved using minimal sample amounts (0.05–0.1 mg) by combining local functional group information obtained from ^1^H NMR and MS measurements with the overall three-dimensional structure visualized within the pores of APF-40. After the interpretation of the corresponding data, fractions D, E and F were identified as other ivermectin B_1a_ derivatives: avermectin B_1a_, 3,4-dihydroivermectin B_1a_ and never before reported compound termed ethyl-ivermectin. Their existence can be rationalized based on known chemical and biochemical processes. Specifically, avermectin B_1a_ is an unreacted intermediate in the ivermectin B_1a_ chemical synthesis.^52^ 3,4-Dihydroivermectin B_1a_ is the product of over-reduction.^52^ Finally, the ethyl-ivermectin compound could have arisen from incorporation of butyric acid instead of propionic acid (Figure S57).^53,54^

More specifically, in the ^1^H NMR spectrum of fraction D, two doublet-of-doublet signals corresponding to the alkene protons appeared in low-magnetic field (δ_H_ = 5.72 and 5.52) (Figures S39 and S40), suggesting that the structure deviation was from dehydrogenation of the C22–C23 single bond. This conclusion was fully consistent with the MS results showing a decrease in molecular weight (Δ*m*/*z* = – 2.018) corresponding to a H_2_ unit (Figure S38). SCXRD analysis within APF-40 unambiguously confirmed the flattened six-membered ring arising from the C22–C23 double bond as observed in the high-resolution electron density map (Figure 8C and Figure S54). From this structural difference, fraction D could be assigned as avermectin B_1a_.

Fraction E was identified as a derivative of ivermectin B_1a_ where the cyclohexene moiety was replaced with the fully hydrogenated cyclohexane. This assignment was made based on the appearance of multiplet signals in the alkane proton region (δ_H_ = 1.24–1.82) accompanied by the disappearance of the singlet alkene proton at C3 (δ_H_ = 5.38) in the ^1^H NMR spectrum (Figures S42 and S43). Furthermore, an increase in molecular weight (Δ*m*/*z* = +2.015) due to the addition of a H_2_ unit was detected in the MS data (Figure S41). Although these results confirmed hydrogenation at the C3–C4 bond, the absolute configuration of C4 remained unclear due to insufficient sample for 2D NMR analysis. Subsequent SCXRD analysis using APF-40 as a crystalline sponge revealed a chair conformation of the cyclohexane motif containing the C3–C4 single bond and unambiguously determined the absolute configuration of C4 (Figure 8C and Figure S55).

In fraction F, MS displayed an increase in molecular weight (Δ*m*/*z* = +14.017) corresponding to a CH_2_ unit (Figure S44). At the same time, the ^1^H NMR spectrum featured a new triplet signal at 0.85 ppm, indicative of an additional alkyl group which was accompanied by the disappearance of the doublet at 0.78 ppm assigned to the C24a methyl protons in the spiroketal moiety of ivermectin B_1a_. These results suggested a substitution of the methyl group with ethyl (Figures S45 and S46). The residual electron density map (2Fo–Fc) from the SCXRD analysis using APF-40 confirmed the existence of the C24b ethyl group attached to the C24 atom in the spiroketal motif (Figure 8C and Figure S56), thus validating the structure assignment.

The five derivatives of ivermectin B_1a_ obtained after HPLC separation and visualized inside APF-40 were immobilized as intermolecular dimers, similar to doramectin and ivermectin B_1a_ (Figures S52 to S56). Due to this thermodynamically favorable assembly mediated by multipoint hydrogen bonding networks surrounding guest dimers, the analyzed molecules were encapsulated with high occupancies (60–85.1%). They exhibited atomically resolved electron density maps, which enabled accurate determination of functional group variations, including bond type, alkyl substitution and stereochemical configuration assignment of all chiral centers. The full analysis could be completed within one month using as little as 25 μg of sample isolated after a single HPLC run. Although ivermectin has been extensively studied since its initial discovery in 1974 with more than 1,000 derivatives identified,^47,48^ the fraction F compound represents a new addition to this vast collection with no registered CAS number. This result offers a powerful demonstration of the effectiveness of microscale precision structure analysis of compounds exceeding 500 Da. The molecules hidden in plain sight as trace impurities in commercially available reagents are often overlooked by conventional analytical methods. However, they can be uncovered and identified using the synergistic combination of HPLC separation coupled with crystalline sponge structure elucidation.

Returning back to the original motivation for the HPLC separation study, the structure of purified ivermectin B_1a_, isolated as fraction A, was reanalyzed inside APF-40 to examine the changes in ADPs around the spiroketal region (Figure S51). After purification, the values were reduced to levels comparable with other atoms in the ivermectin B_1a_ molecule (Figure S58). Guest contamination with structurally similar components strongly affected model refinement by introducing localized ADP anomalies, which can then serve as indicators of sample purity helping to decide whether HPLC separation is necessary or not.

### Host–Guest Interactions

The series of successfully analyzed compounds encompass a wide range of molecular weights and interactions. The accompanying host–guest binding energies were calculated using the resultant crystal structure models to gain deeper understanding of the immobilization phenomena inside large pore MOFs. First, the extent of framework deformation due to guest accommodation was evaluated by plotting distances between Zn_8_ clusters of different interpenetrated nets representing the pore corners against the binding energies (Figure S59). The results revealed that the greater net displacement, expressed by the dimensions of encapsulation pocket in APF-40 (*d*_1_ and *d*_3_) (Figure S59, A and B), correlated with increased stabilization of the host–guest structure, indicative of adaptive behavior. Next, the binding energies were correlated with molecular weights of encapsulated guests in both the larger-pore APF-40 (17.4 × 12.5 Å^2^) and previously reported smaller-pore APF-80 (8.34 × 4.36 Å^2^).^24^ The resultant plots exhibited smaller intercept and slope values for APF-40 compared to APF-80 (Figure S60). This behavior implies an increasing difficulty of forming stable host–guest assemblies inside more porous structures with less reliance on molecular sizes alone. As a consequence, to achieve sufficient guest immobilization, other adaptable structural features, such as adjustable sliding of interpenetrated nets, are required to compensate for the inherent lack of interactivity of large void spaces inside frameworks.

## Conclusion

In this study, a novel benzoate-substituted hexaazaphenalenyl ligand (H_4_TCHAP) featuring *C*3-symmetry was developed. Screening of previously reported MOF literature for structures incorporating similarly shaped ligands guided the design of a new framework denoted as APF-40. It was crystallized from a mixture of H_4_TCHAP combined with Zn^2+^ ions, and 1,2,3-triazolate and 2,5-thiophenedicarboxylate coligands under solvothermal conditions. APF-40 contains doubly interpenetrated three-dimensional networks constructed from octanuclear Zn clusters interconnected by HTCHAP^3–^ linkers, supporting large channels that surpassed other MOFs previously employed in the crystalline sponge method. The channel surfaces are decorated with hydrogen-bonding networks mediated by water molecules and dimethylammonium (DMA^+^) cations, providing auxiliary hydrophilic interaction sites that could adapt to the shape and interaction preferences of encapsulated guests. Because of these features, APF-40 facilitated structure determination of seven pharmaceutical compounds, including doramectin (MW = 899.13 Da, 64 non-hydrogen atoms), which establishes the new size benchmark for guest structures solved by the crystalline sponge method. SCXRD analysis of encapsulated APF-40 crystals revealed that guests were immobilized by hydrogen-bonding networks formed between the framework backbone, water molecules and DMA^+^ cations. The doubly interpenetrated MOF scaffold also demonstrated the ability to expand or contract its pore dimensions to accommodate molecules of diverse sizes and shapes. Furthermore, when guests were too small relative to the pore dimensions, they formed larger dimeric assemblies inside the channels through hydrogen bonding or coordination to Zn^2+^ ions, thereby counteracting the size mismatch. This dimerization behavior broadly occurred across different encapsulated molecules, representing a distinctive feature not commonly observed in other crystalline sponge systems. These observations highlight a unique mutual adaptability between APF-40 and its guests: the framework adjusts its pore geometry to capture the guest, which in turn further self-assembles inside the pore to maximize the interactions, collectively stabilizing the resultant host–guest structures.

In the course of these studies, unexpectedly large ADPs were detected in the spiroketal moiety of encapsulated ivermectin B_1a_. This anomaly was attributed to the presence of structurally similar compounds occupying the same crystallographic site. The commercial ivermectin sample was therefore subjected to HPLC purification, followed by complementary integrated structural analysis combining NMR, MS and the crystalline sponge method. Six ivermectin derivatives were isolated as pure compounds in microgram quantities, which was insufficient for detailed NMR characterization, such as 2D NMR. In particular, one demethylated saccharide derivative could not be unambiguously assigned by NMR and MS alone. Nevertheless, single-crystal X-ray diffraction analysis using APF-40 enabled structure determination of all six compounds, including their absolute configurations. This analytic strategy also led to the identification of a previously unknown derivative termed ethyl-ivermectin, which was present as a trace component (<0.1%) in the commercial sample. Comparison of the structural models of ivermectin B_1a_ before and after HPLC separation confirmed that the anomalously high ADP values decreased to levels comparable with other atoms once impurities were removed. This result demonstrated that concomitant encapsulation of structurally similar molecules can significantly affect crystallographic refinement. Therefore, monitoring for unreasonable ADP anomalies can serve as an indicator of sample purity and help to judge the feasibility of HPLC purification. Collectively, these findings demonstrate the effectiveness of accurate microgram scale structure determination of compounds exceeding 500 Da using APF-40 as a crystalline sponge matrix. The developed methodology has the potential to become broadly applicable for the structural elucidation of difficult-to-analyze molecules in trace quantities.

## Supplementary Material



## Notes

Experimental Section: materials; general methods; single-crystal X-ray structural analysis; synthetic procedures and characterization of TBA­(TTHAP); H_4_TCHAP and APF-40; solvent exchange of APF-40 and guest encapsulations in APF-40; experimental scheme of HPLC separation of the commercial ivermectin B_1a_ sample; characterization of six ivermectin derivatives separated from the commercial ivermectin B_1a_ sample; crystallographic results: structures of all guest sites; comparison of isotropic displacement parameters of ivermectin B_1a_ before and after HPLC separation; crystallographic restraints and constraints; calculation results of MOF-guest binding energy; crystallographic tables; checkCIF alerts and their responses (PDF)
