# Supplementary material for: An Adaptable Porous Metal–Organic Framework for Structural Visualization of Beyond Rule of 5 Molecules on the Microgram Scale
Source: J Am Chem Soc. 2026 Aug 4;148(32):34430–41. doi: 10.1021/jacs.6c08312 (PMC13495750; doi:10.1021/jacs.6c08312)
Supplement: Supplementary file 1 [file ja6c08312_si_001.pdf]

Supplementary Information

An Adaptable Porous Metal–Organic Framework for Structural Visualization of Beyond Rule of 5 Molecules on the Microgram Scale

Taichi Baba†, Yu Tagami†, Natsumi Watanabe, Bun Chan, Tomoki Nakagawa, Yiying Zhu, Arihiro Iwasaki*, Pavel M. Usov, Yuki Wada*, Masaki Kawano*

†These authors contributed equally to this work.

*Corresponding author. A. Iwasaki, Email: aiwasaki686@g.chuo-u.ac.jp, Y. Wada, Email: yuki.wada@chem.sci.isct.ac.jp, M. Kawano, Email: mkawano@chem.sci.isct.ac.jp.

Contents

1. Materials and Methods section. S4
1.1 Materials S4
1.2 General methods S4
1.3. Single crystal X-ray structural analysis. S5
2. Experimental section S6
2.1. Synthesis of tetrabutylammonium 2,5,8-tris(4′-methylphenyl)-1,3,4,6,7,9-hexaazaphenalenyl (TBA(TTHAP)). S6
2.2. Synthesis of 2,5,8-tris(4′-carboxyphenyl)-1,3,4,6,7,9-hexaazaphenalene (H_4TCHAP). S7
2.3 Synthesis of APF-40. .... S8
3. Characterization of ligands and APF-40. .... S9
4. Structural determination of guest molecules by encapsulation within APF-40. .... S17
4.1. Representative crystalline sponge materials. S17
4.2. General procedure for guest encapsulation into APF-40 .... S18
4.3. Single crystal X-ray analysis of small molecules in APF-40. .... S20
4.4. Single crystal X-ray analysis of compounds containing flexible moieties in APF-40. .... S24
4.5. Characterization of fulvestrant extracted from the guest-loaded APF-40 crystals S26
4.6. Single crystal X-ray analysis of bRo5 compounds in APF-40. .... S27
5. HPLC separation and characterization of six ivermectin derivatives from the commercial reagent. S30
5.1. Experimental scheme of HPLC separation S30
5.2. HPLC charts. S31
5.3. Characterization of HPLC-separated ivermectin derivatives by MS and ^{1}H NMR. S34
5.4. ^{1}H NMR signals of fraction A, B, C, D, E and F. S36
5.5. Characterization of fraction A by MS and ^{1}H NMR. S38
5.6. Structural characterization of fraction B by MS and ^{1}H NMR S39
5.7. Structural characterization of fraction C by MS and ^{1}H NMR S41
5.8. Structural characterization of fraction D by MS and ^{1}H NMR. S43
5.9. Structural characterization of fraction E by MS and ^{1}H NMR. S45
5.10. Structural characterization of fraction F by MS and ^{1}H NMR. S47
5.11. ^{1}H NMR spectra of mixed fraction 1, 2, 3 and 4. .... S49
6. Structural determination of HPLC-separated ivermectin derivatives by encapsulation within APF-40. .... S51
6.1. Single crystal X-ray analysis of ivermectin derivatives. S51

6.2. Proposed biosynthetic mechanism for the introduction of ethyl group into ethyl-ivermectin. S57
6.3. Comparison of isotropic displacement parameters of ivermectin B_{1a} before and after HPLC separation. S58
7. MOF-guest binding energy calculation S59
7.1. Calculation methods. S59
7.2. Correlation between binding energy and framework deformation. S60
7.3. Correlation between molecular weight of the guests and binding energy S61
7.4. Summary of calculated binding energies and crystallographic occupancies. S62
8. Details about the crystallographic analysis of guest-loaded APF-40. ....S63
8.1. Visualization of crystallographic restraints and constraints. S63
8.2. Crystallographic tables. S66
8.3. A or B- level checkcif alerts and their responses. S72
9. References. S88

1. Materials and Methods section.

1.1 Materials

Solvents and reagents for ligand and MOF syntheses were purchased from KANTO CHEMICAL CO., INC., TCI Co., Ltd., FUJIFILM Wako Pure Chemical Corporation and Sigma Aldrich Co., and used without further purification. For the encapsulation experiments, solvents and guest compounds were purchased from TCI Co., Ltd. and FUJIFILM Wako Pure Chemical Corporation, and used without further purification except for ivermectin B_{1a} (Lot number: K3CSD-DT).

1.2 General methods

^{1}H and ^{13}C{^{1}H} NMR spectra for TBA(TTHAP) and H_4TCHAP were collected on a JEOL JNM-ECA400 II spectrometer. ^{1}H NMR chemical shifts were referenced to the signal of tetramethylsilane (TMS) as an external reference (TMS: δ 0.00). ^{13}C{^{1}H} NMR chemical shifts were referenced to the residual solvent signal (($(CHD_2)_2SO$: δ 39.52). ^{1}H NMR spectra for ivermectin B_{1a} before HPLC purification, 3′-*O*-demethylivermectin B_{1a}, avermectin B_{1a}, ivermectin B_{1b}, ivermectin B_{1a} after HPLC purification, 3,4-dihydroivermectin B_{1a} and ethyl-ivermectin were collected on a JEOL ECZ-500 spectrometer. ^{1}H NMR chemical shifts were referenced to the residual solvent signal (CHD_2CN: δ 1.94). UV-vis spectra were collected on a JASCO V-770 spectrophotometer. For solution-state measurements, quartz cell (optical path length = 1 cm) was used. The solid-state measurements were performed in the diffuse reflectance mode. The samples were mixed with $BaSO_4$, which was used as a background. Thermogravimetric analysis differential scanning calorimetry (TGA-DSC) was measured using Simultaneous Thermogravimetry STA449F3 Jupiter analyzer NETZSCH. The powdered samples were loaded into Al_2O_3 pans and heated at 5 K/min rate under a flow of N_2. Elemental analyses were measured using JScience JM-10 instrument. Fourier transform infrared (FTIR) spectra were measured on a Nicolet iS5 spectrometer from Thermo Fisher Scientific using attenuated total reflectance (ATR) method. Rigaku Miniflex powder diffractometer with D/teX Ultra (1D) detector using Cu Kα radiation was used for PXRD measurements. The powder sample suspended in DMF was placed onto a silicon plate and covered by a Mylar film. ESI mass spectra were obtained on a JEOL JMS-T100LP AccuTOF LC-plus 4G in methanol. COSMOSIL Cholester Packed Column (ϕ 20 mm × 250 mm) and COSMOSIL 5C_{18}-MS-II Packed Column (ϕ 20 mm × 250 mm) from Nacalai Tesque were used for reversed-phase column chromatography. Analytical high-performance liquid chromatography (HPLC) was performed using a pump (PU-4580, JASCO or LC-10ADVP, Shimadzu) and a UV detector (MD-4015 or MD-2010, JASCO). Preparative HPLC was carried out using a pump (PU-4580, JASCO) and a UV detector (UV-4575, JASCO).

1.3. Single crystal X-ray structural analysis.

The diffraction data for TBA(TTHAP) was recorded on a Rigaku Synergy-R/DWTI APEX II instrument with a Hypix-6000HE detector equipped with Rigaku GNNP low temperature device using PhotonJet-R X-ray source with MicroMax™-007 rotating anode Cu Kα radiation (λ = 1.54184 Å). The diffraction data were reduced and corrected using CrysAlisPro 1.171.40.84a. For all other single crystals, the X-ray analysis was performed on a diffractometer equipped on the synchrotron beamline BL-5A at KEK (the High Energy Accelerator Research Organization, Japan) with a Pilatus3 S6M detector (λ = 0.7500 Å, T = 95 K). XDS[1] software was used for processing and data reduction. The structures were solved by Dual space methods (SHELXT-2018)[2] and refined by full-matrix least squares calculations on F^2 (SHELXL-2018)[3] using the Olex2 program package[4]. The reflections were cut off using the SHEL instruction in SHELXL based on the high-resolution limit defined by XDS data processing. All hydrogen atoms except those on water molecules and hydroxyl or thiol groups were placed in ideal positions and refined using a riding model. All non-hydrogen atoms, except for the low-occupancy encapsulated dexamethasone, were refined with anisotropic displacement parameters. The dexamethasone atoms were refined with isotropic displacement parameters.

2. Experimental section

2.1. Synthesis of tetrabutylammonium 2,5,8-tris(4′-methylphenyl)-1,3,4,6,7,9-hexaazaphenalenyl (TBA(TTHAP)).

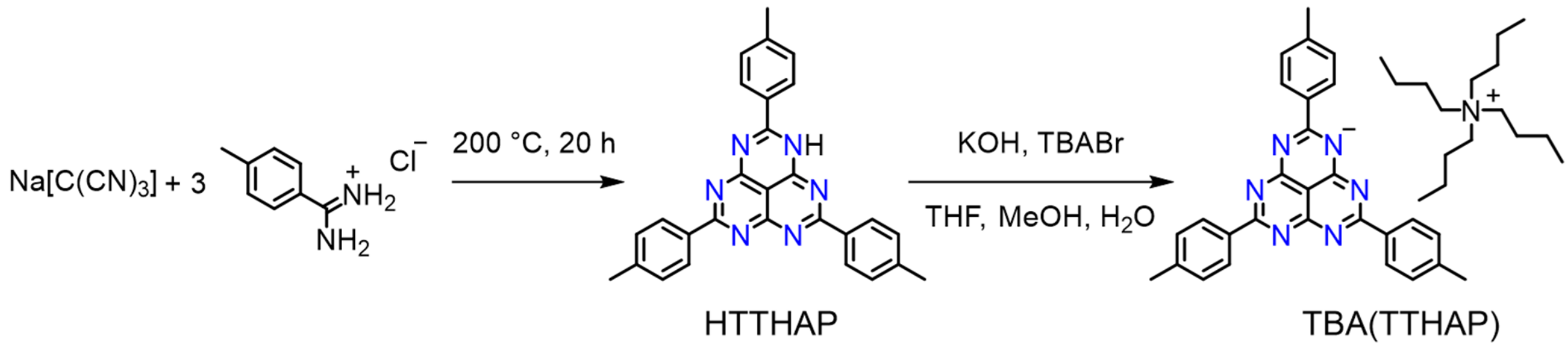

Scheme S1. Synthesis of TBA(TTHAP).

Sodium tricyanomethanide (0.80 g, 7.1 mmol) and 4-methylbenzamidine hydrochloride (4.12 g, 23.4 mmol) were placed in a Teflon-lined stainless-steel autoclave and heated at 200 °C for 20 h. The resultant brown solid was dissolved in DMSO (*ca.* 40 mL) with heating at 110 °C. Then, water (150 mL) and toluene (150 mL) were added to the solution causing the precipitation of brown solid. It was collected by filtration and dried *in vacuo* to give 1.79 g of crude 2,5,8-tris(4′-methylphenyl)-1,3,4,6,7,9-hexaazaphenalene (HTTHAP), which was subsequently purified during the conversion to the tetrabutylammonium salt via deprotonation and cation exchange. The HTTHAP solid (0.78 g, 1.8 mmol) was dissolved in a mixture of tetrahydrofuran (50 mL), methanol (50 mL) and 1 M aqueous potassium hydroxide solution (50 mL). Tetrabutylammonium bromide (1.10 g, 3.41 mmol) was added, and the mixture was stirred until complete dissolution. After evaporating tetrahydrofuran and methanol, dichloromethane was added to the remaining aqueous phase. The organic layer was separated and washed with water and brine to remove the residual salts. The organic solution was reduced to dryness to give a brown solid. It was dissolved in a minimal amount of DMSO. Water was added to the resultant solution until precipitation has ceased. The precipitate was isolated by filtration to give the desired TBA(TTHAP) product as a pale yellow solid (0.72 g, 1.1 mmol, yield = 61%). Anal. Calcd. for $C_{44}H_{57}N_7(C_3H_7NO)_{1.79}$ = $C_{49.49}H_{68.81}N_{8.83}O_{1.83}$: C, 72.78; H, 8.49; N, 15.14. Found: C, 72.78; H, 8.23; N, 15.22%. Absorption maxima (in acetonitrile) [λ_{max} / nm (ε / L·mol^{-1}·cm^{-1})]: 280 (6.3 × 10^4), 322 (1.2 × 10^4) (Fig. S1). ^{1}H NMR (400 MHz, DMSO-d_6) δ 8.44 (d, *J* = 7.8 Hz, 6H), 7.33 (d, *J* = 7.8 Hz, 6H), 3.09 (t, *J* = 8.5 Hz, 8H), 2.41 (s, 9H), 1.54–1.47 (m, 8H), 1.25 (td, *J* = 14.5, 7.2 Hz, 8H), 0.89 (t, *J* = 7.2 Hz, 12H) (Fig. S2). ^{13}C{^{1}H} NMR (101 MHz, DMSO-d_6) δ 167.4, 166.6, 139.7, 136.9, 128.7, 128.3, 102.0, 57.5, 23.0, 21.0, 19.1, 13.4 (Fig. S3). Single crystals were obtained by vapor diffusion of diethyl ether into a DMF solution of TBA(TTHAP) (Fig. S4).

2.2. Synthesis of 2,5,8-tris(4′-carboxyphenyl)-1,3,4,6,7,9-hexaazaphenalene (H_4TCHAP).

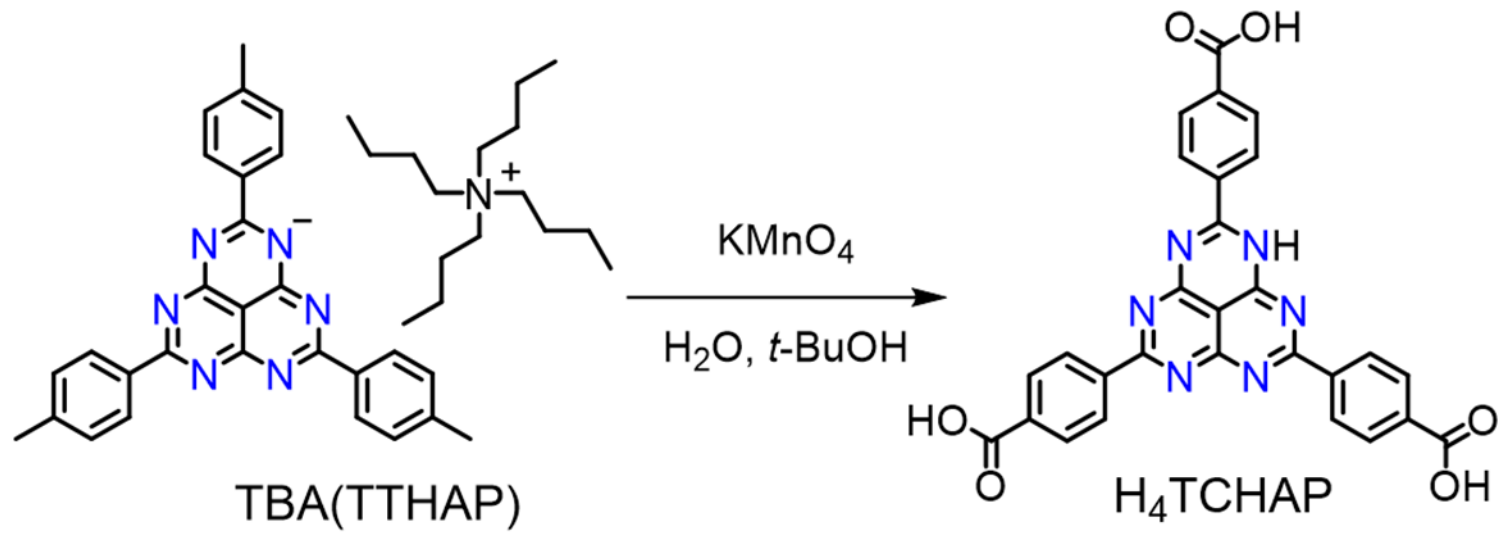

Scheme S2. Synthesis of H_4TCHAP.

TBA(TTHAP) (1.00 g, 1.46 mmol) and potassium permanganate (3.48 g, 22.0 mmol) were placed in a round bottom flask followed by addition of water (100 mL) and *tert*-butyl alcohol (50 mL). The reaction mixture was heated at 100 °C for 24 h. After cooling to room temperature, the solid was removed by filtration. 4 M aqueous hydrochloric acid solution was added to the resulting yellow filtrate until the pH was below 3. The precipitated yellow solid was collected by filtration. Then, it was dissolved in DMSO with heating at 80 °C. Ethanol was added dropwise to the resultant hot solution until it become cloudy. It was then cooled down to room temperature causing precipitation of yellow solid, which was isolated by filtration and dried *in vacuo* to give the desired H_4TCHAP (0.35 g, 0.66 mmol, yield = 45%). Anal. Calcd. for $C_{28}H_{16}N_6O_6(C_2H_6OS)_{2.58}(H_2O)_{1.88} = C_{33.16}H_{35.24}N_6O_{10.46}S_{2.58}$: C, 51.88; H, 4.62; N, 10.95. Found: C, 51.88; H, 4.63; N, 10.95. Absorption maxima (in DMF) [λ_{max} / nm (ε / L·mol^{-1}·cm^{-1})]: 284 (7.3×10^4), 336 (1.3×10^4) (Fig. S1). ^{1}H NMR (400 MHz, DMSO-d_6) δ 13.25 (s, 3H), 8.54 (d, J = 8.5 Hz, 6H), 8.11 (d, J = 8.5 Hz, 6H) (Fig. S5). ^{13}C{^{1}H} NMR (101 MHz, DMSO-d_6 + excess amount of TBAOH aqueous solution) δ 169.5, 168.0, 166.8, 142.3, 139.6, 129.0, 127.5, 102.0, 57.6, 23.1, 19.2, 13.5 (Fig. S6). Colorless plate single crystals of H_4TCHAP were obtained by vapor diffusion of ethanol into a DMSO solution (Fig. S7).

2.3 Synthesis of APF-40.

H_4TCHAP (9.0 mg, 17 µmol), ZnI_2 (27 mg, 85 µmol), 1*H*-1,2,3-triazole (HTAZ, 3 µL, 52 µmol) and 2,5-thiophenedicarboxylic acid (H_2TDC, 4.0 mg, 23 µmol) were dissolved in dry DMF (1 mL) and acetic acid (50 µL). The reaction mixture was placed in a Teflon-lined stainless-steel autoclave and heated in an oven at 120 °C for 40 h. After cooling to room temperature, the resultant colorless plate crystals of APF-40 were transferred to a glass vial with the solvent. The suspension was decanted, leaving the crystals behind. Fresh DMF was added to the vial, and the solution was decanted again. This process was repeated three times. The crystals were stored in DMF for PXRD, solvent exchange and guest encapsulation studies (Figs. S8 and S9). For elemental analysis and TGA-DSC (Fig. S10), as well as UV-vis (Fig. S11) and IR spectroscopies (Fig. S12), they were collected by filtration, washed by fresh DMF and acetone, and dried (14 mg, yield = 63%). Anal. Calcd. for $Zn_8(C_{28}H_{13}N_6O_6)_2(C_6H_2O_4S)_3(C_2H_2N_3)_6(C_2H_8N)_2(C_3H_6O)_{2.49}(H_2O)_{10.39}$ = $C_{97.47}H_{95.72}N_{32}O_{36.88}S_3Zn_8$: C, 40.25; H, 3.32; N, 15.41. Found: C, 40.25; H, 3.17; N, 15.41.

3. Characterization of ligands and APF-40.

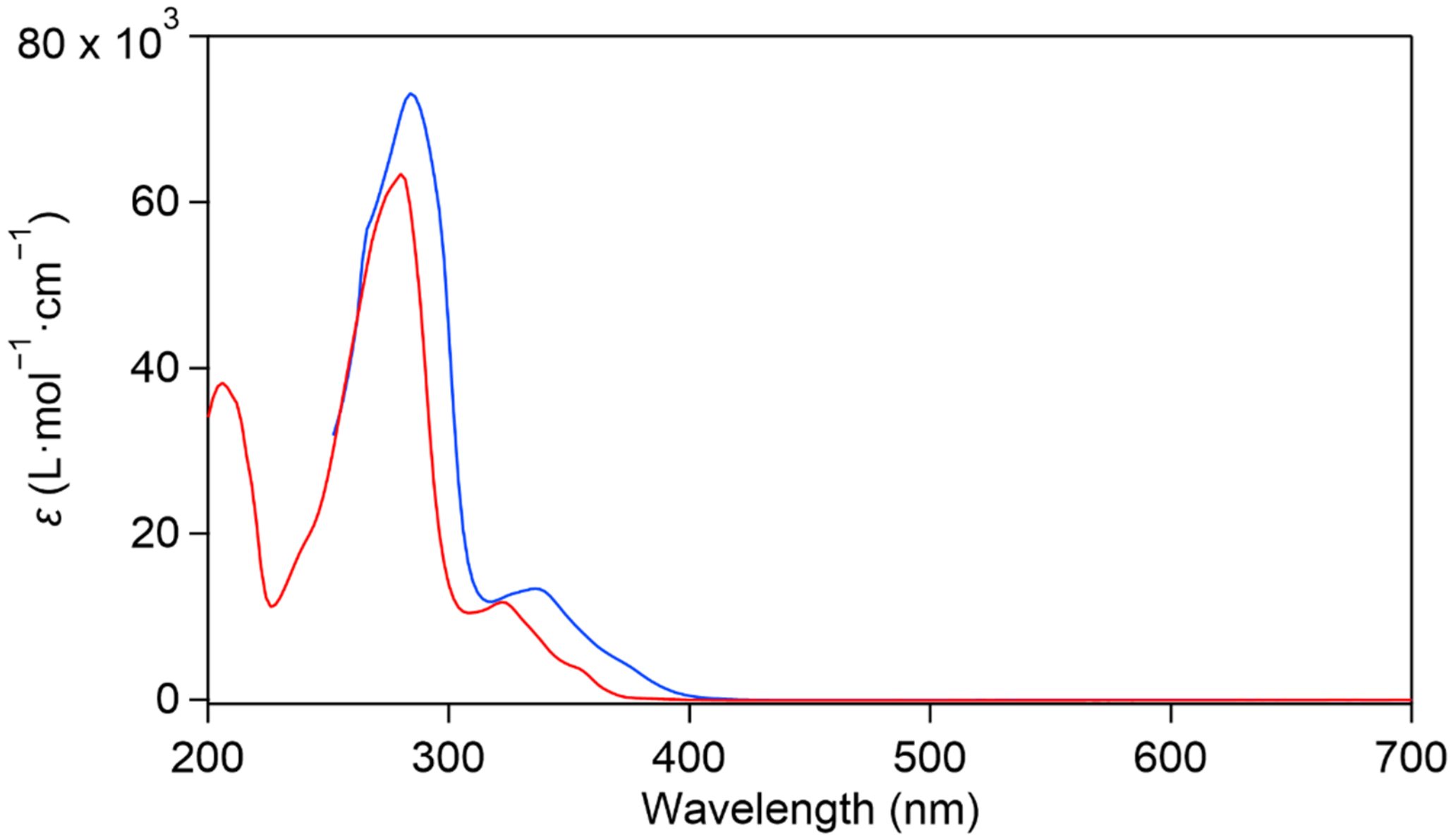

Fig. S1. Solution-state UV-vis spectra of TBA(TTHAP) in acetonitrile (*red*) and H_4TCHAP in DMF (*blue*).

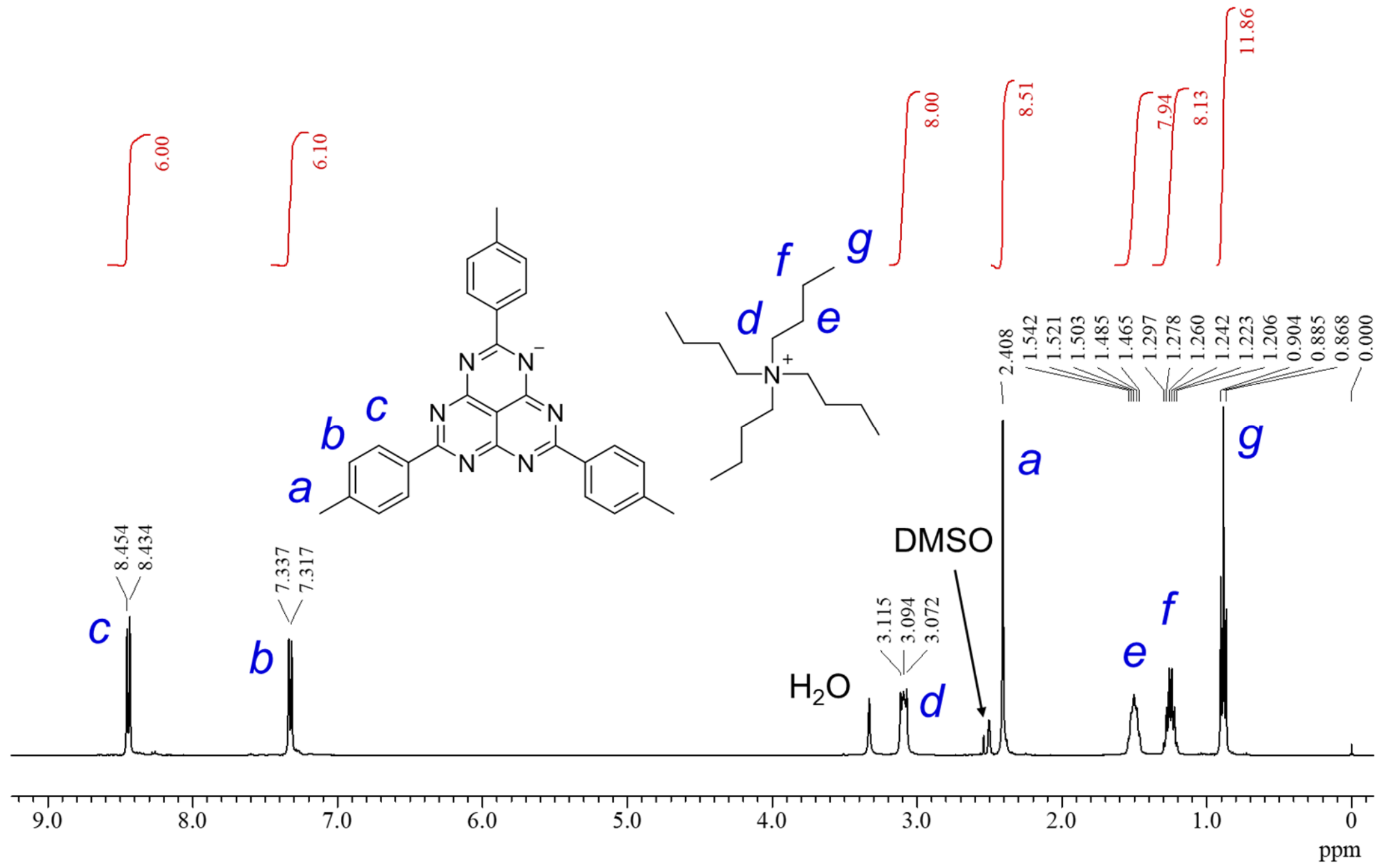

Fig. S2. ^{1}H NMR spectrum of TBA(TTHAP) (DMSO-d_6, 400 MHz).

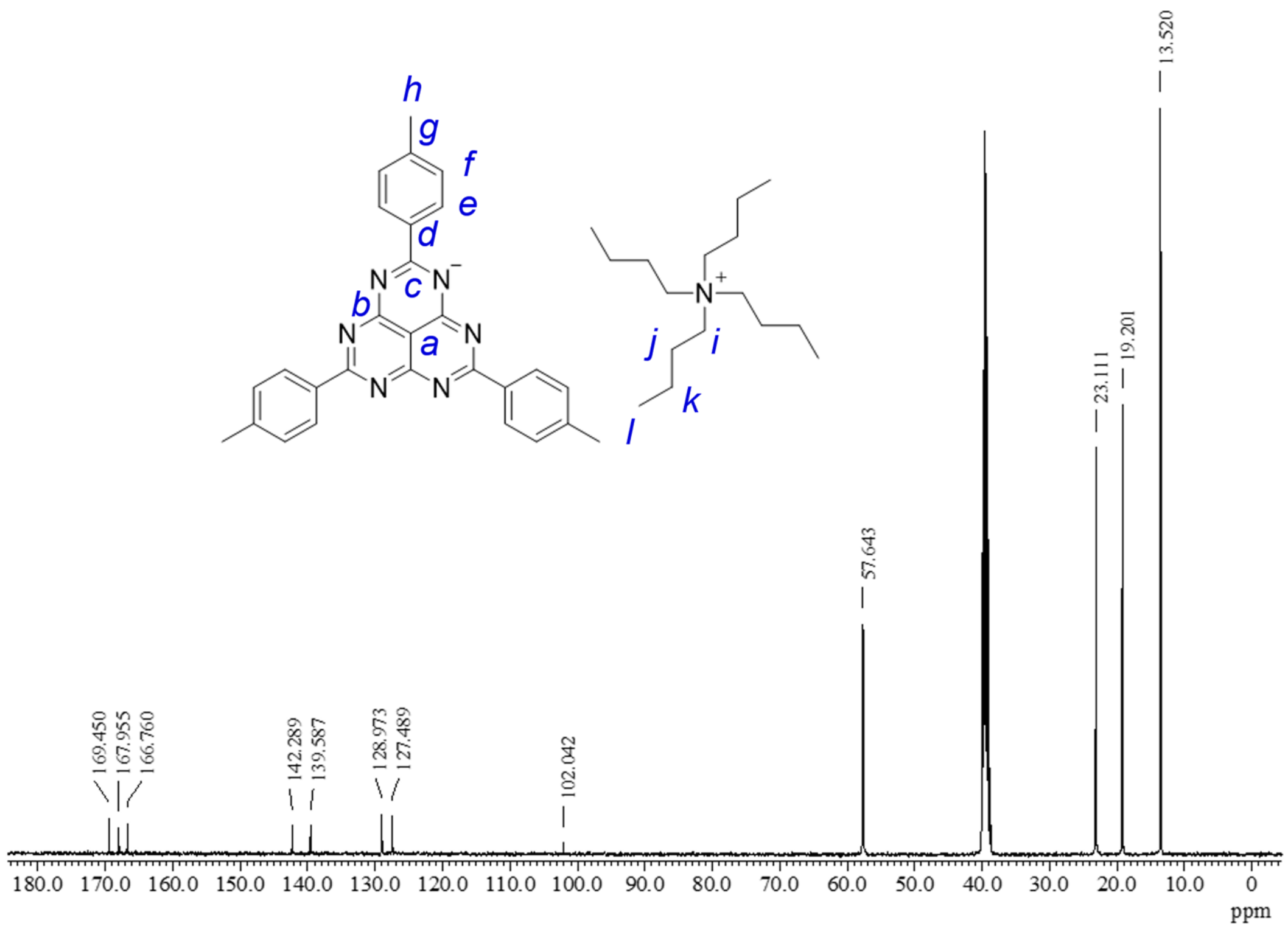

Fig. S3. $^{13}C\{^{1}H\}$ NMR spectrum of TBA(TTHAP) (DMSO-d_6, 101 MHz).

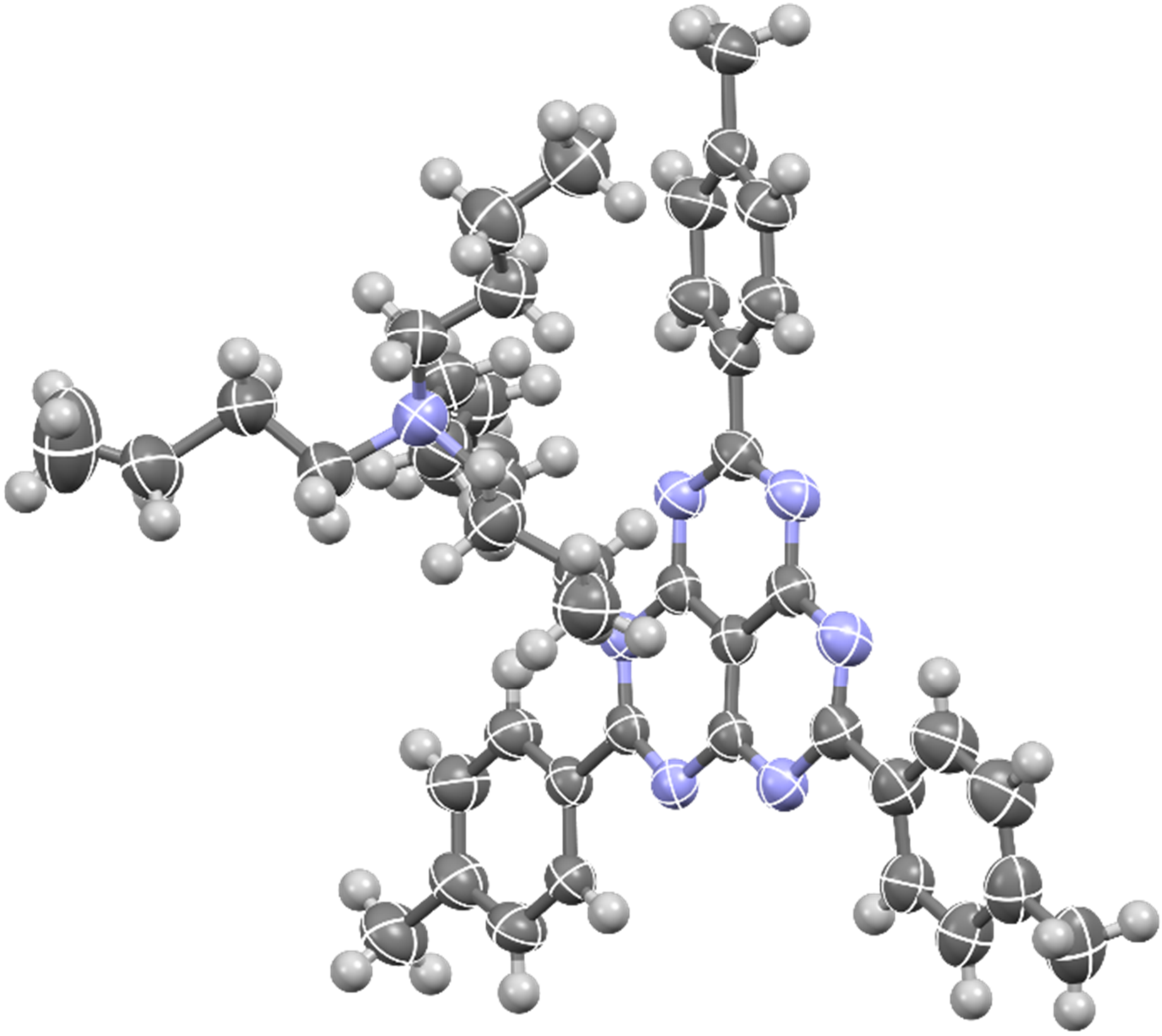

Fig. S4. The single crystal X-ray structure of TBA(TTHAP). C – grey, H – white and N – blue.

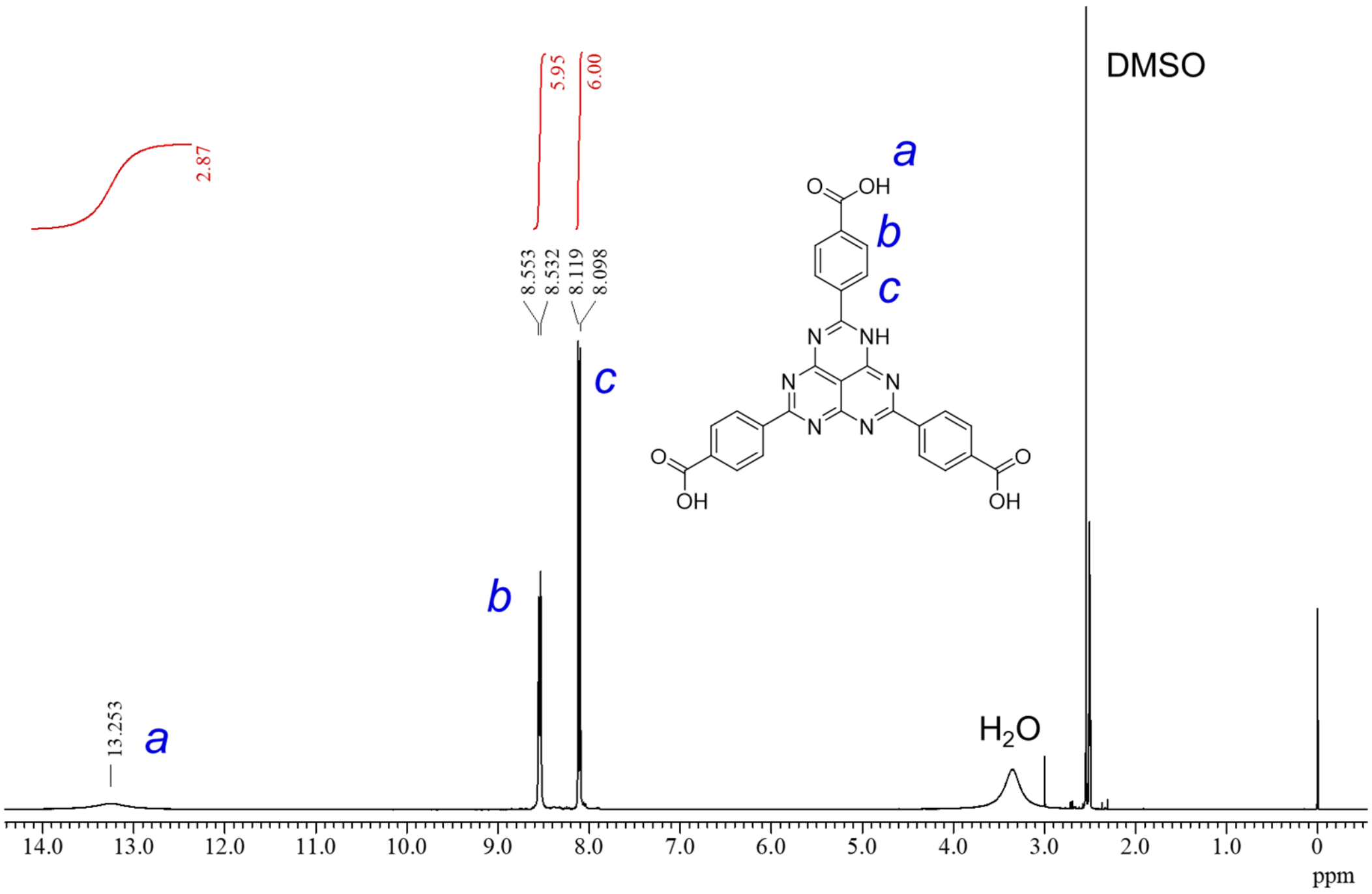

Fig. S5. ^{1}H NMR spectrum of H_4TCHAP (DMSO-d_6, 400 MHz).

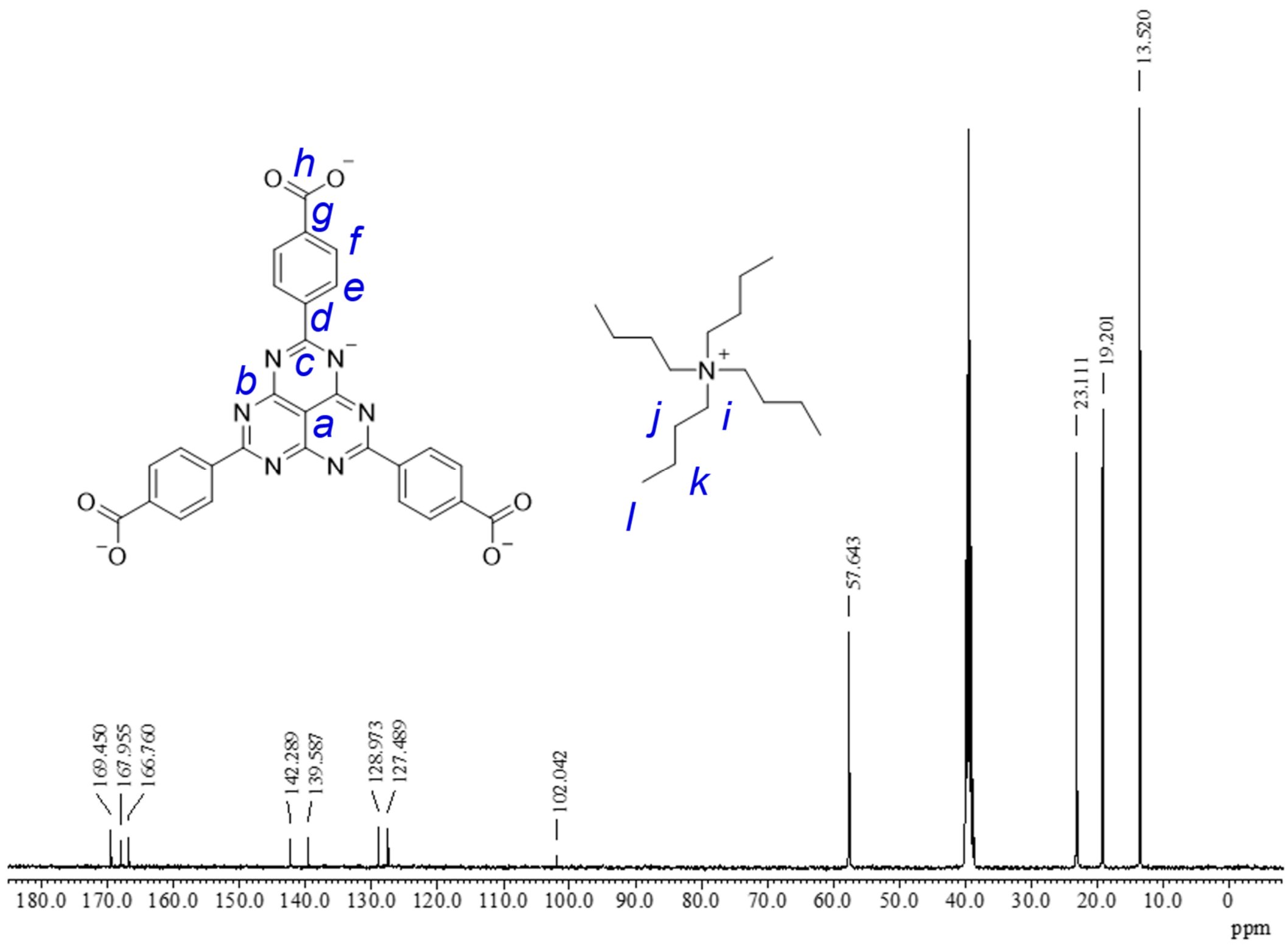

Fig. S6. $^{13}C\{^{1}H\}$ NMR spectrum of H_4TCHAP (DMSO-d_6 + TBAOH aqueous solution, 101 MHz).

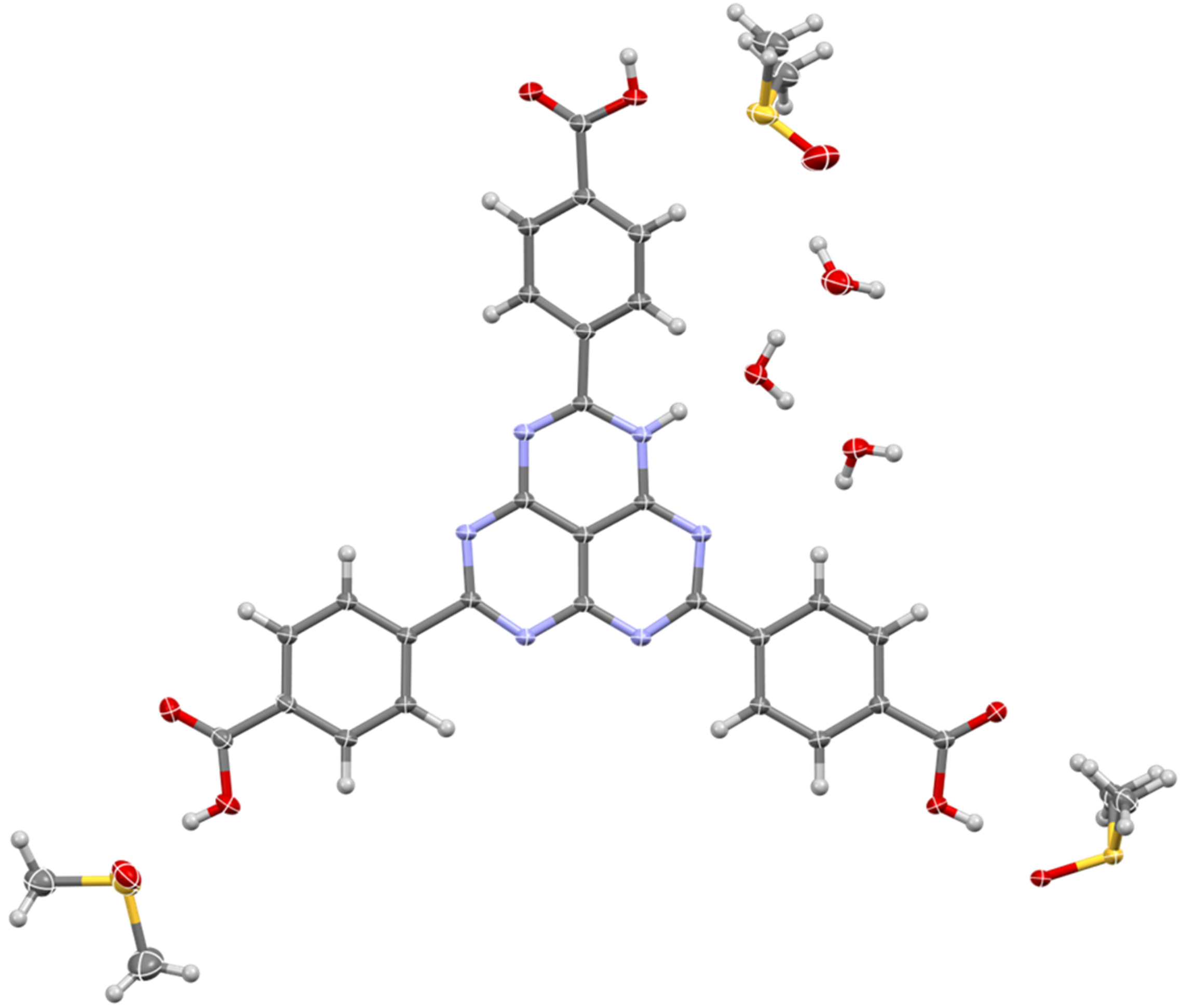

Fig. S7. The single crystal X-ray structure of H_4TCHAP. C – grey, H – white, O – red, N – blue and S – yellow.

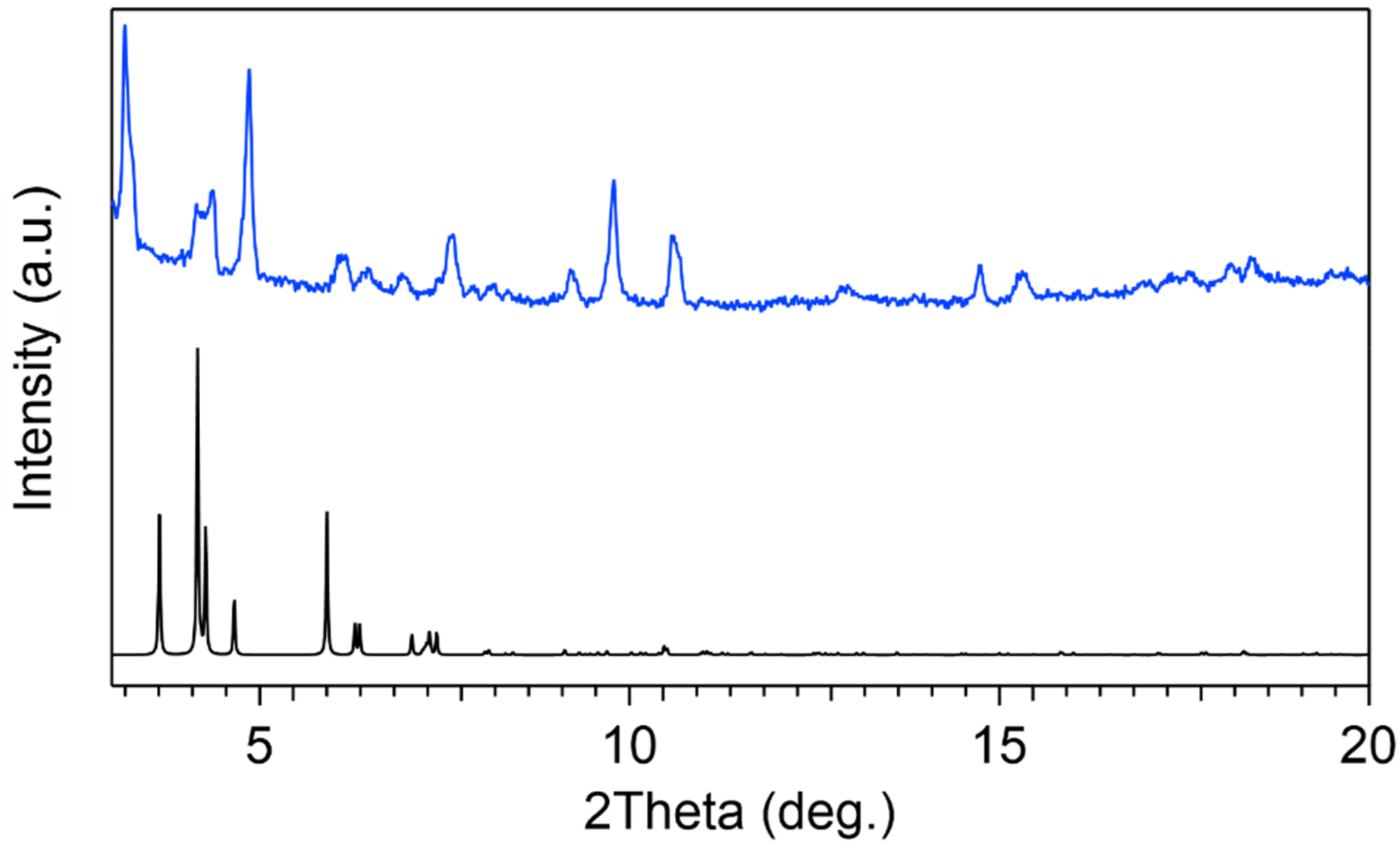

Fig. S8. Powder X-ray diffraction pattern of APF-40 measured in DMF (*blue*) compared to the simulated pattern from the single crystal structure (*black*).

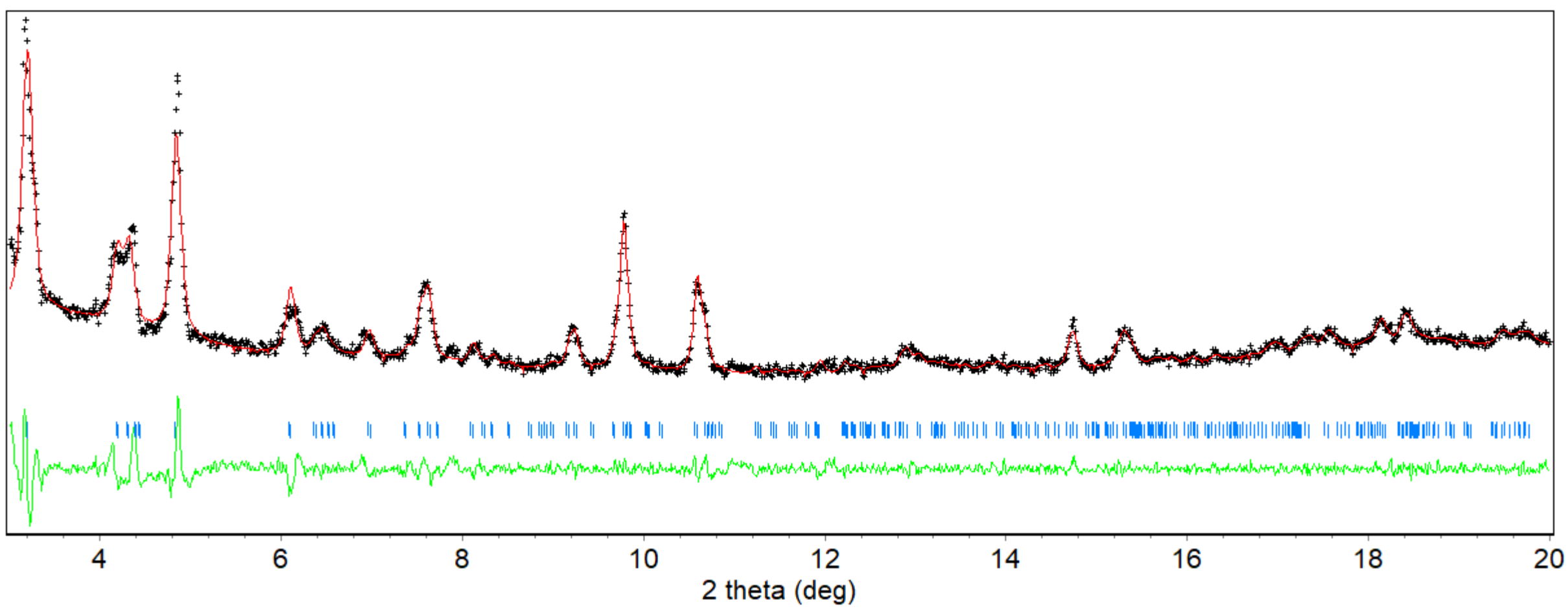

Fig. S9. Le Bail refinement of the APF-40 powder pattern measured in DMF. The measured pattern (*black crosses*), Le Bail fit (*red solid line*), residual (*green solid line*) and allowed reflections for the *C*2/*m* space group at Cu Kα_1 and Kα_2 radiation wavelengths (*blue tick marks*). Fitted unit cell parameters: a = 45.10(1) Å, b = 35.72(1) Å, c = 23.17(1) Å, β = 117.83(2)°.

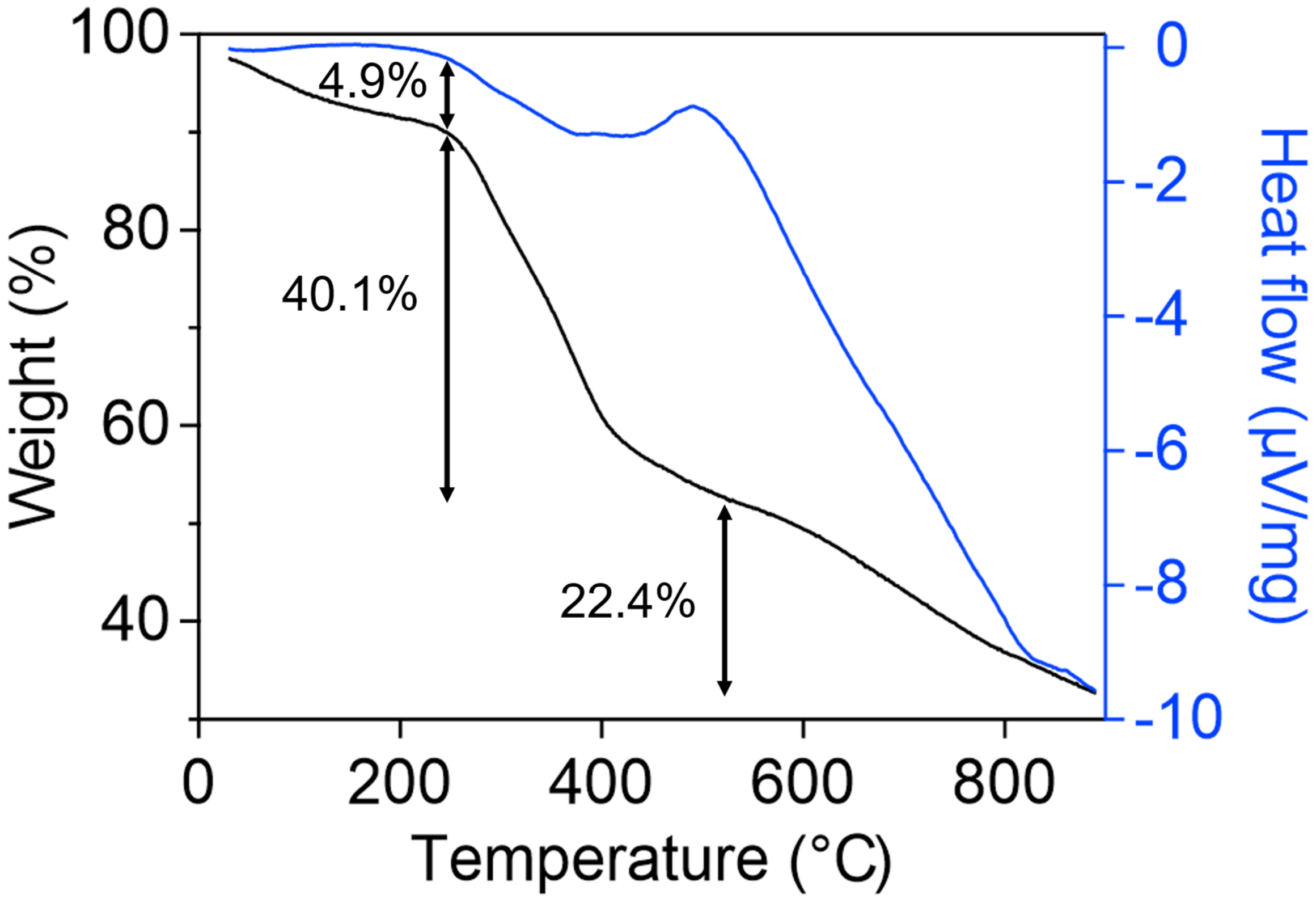

Fig. S10. TGA-DSC data of APF-40.

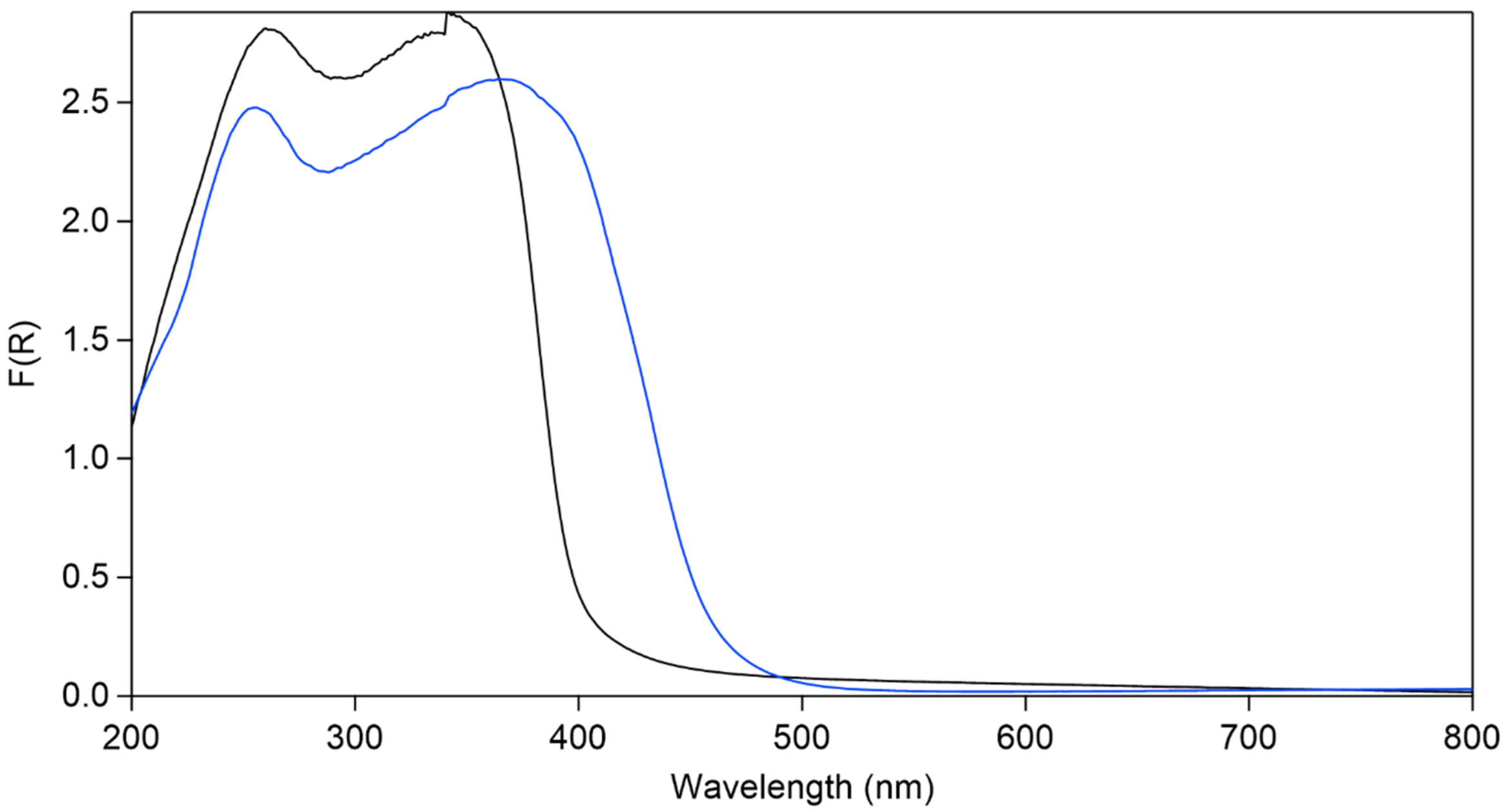

Fig. S11. Diffuse reflectance UV-vis spectra of H_4TCHAP (*blue*) and APF-40 (*black*).

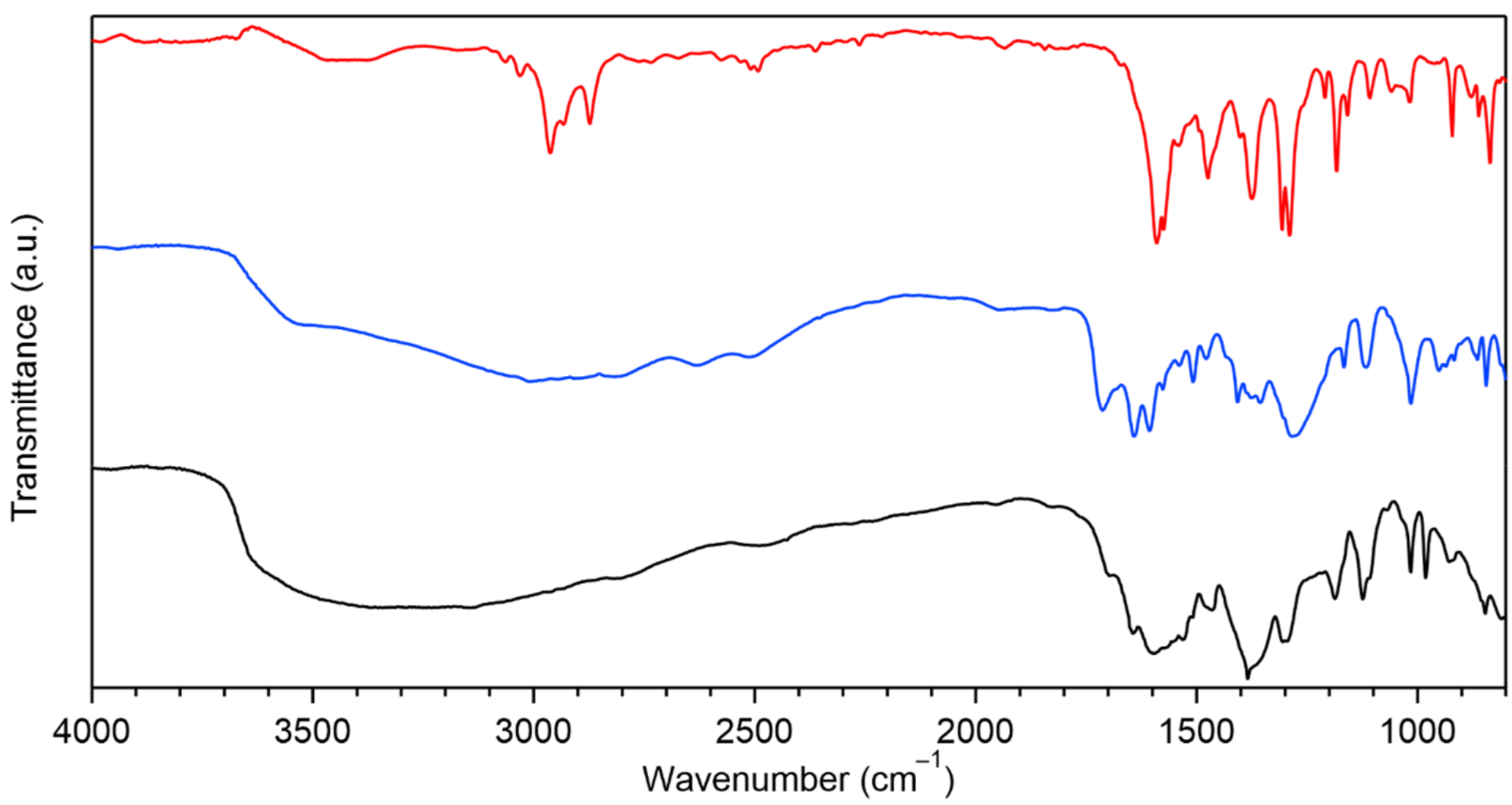

Fig. S12. IR spectra of TBA(TTHAP) (*red*), H_4TCHAP (*blue*) and APF-40 (*black*).

4. Structural determination of guest molecules by encapsulation within APF-40.

4.1. Representative crystalline sponge materials.

Table S1. Representative crystalline sponge materials. The pore sizes were calculated using pore analyzer function in CCDC Mercury. Materials are ordered by the number of non-hydrogen atoms of the largest analyzed guest. Guest detail, including the amounts used, was taken from the supporting information of the respective manuscripts.

Material	Pore size (Å^2)	Largest guest molecule name	Number of non-hydrogen atoms	MW (Da)	Sample amount	Reference
APF-40	17.4 × 12.5	Doramectin	64	899.13	4.0 mg	This work
EtP5-MOF-2	8.49 × 3.90	Oleic acid-substituted amine	48	678.10	5 μL	5
APF-1	10.5 × 5.87	Betamethasone 17,21-dipropionate	36	504.30	2.0 mg	6
ZnI_2-TPT	9.07 × 5.78	Orlistat	35	495.74	1 μg	7
MOF-520	10.3 × 7.75	Entospletinib	31	411.47	3 mg	8
BTB-MOF-24	6.62 × 4.46	Squalene	30	410.73	36 μg	9
APF-80	8.34 × 4.36	Voriconazole	25	349.32	1.2 mg	10
MMFs	10.9 × 10.2	*N*-(benzyloxycarbonyl)-L-seryl-L-proline methyl ester	25	350.37	Not available	11
Pd_6TPT_4 cage	12.9 × 10.8	Methylandrostenediol	22	304.47	20 μg	12
Ag-based MOF	8.95 × 6.03	Perfluorohexane	20	338.04	20 μL	13
CaCu-based MOF	8.64 × 6.92	β-Estradiol	20	272.39	Not available	14
CMOF	11.0 × 8.84	Benzyl 2-hydroxybenzoate	17	228.25	200 μL	15
RUM-2	6.48 × 4.13	Benzyl benzoate	16	212.25	1 mL	16
$[CuBr(btt)]_n$	9.74 × 9.20	1-acetonaphthone	13	170.21	50 μL	17
PUM168	9.10 × 5.15	Eugenol	12	164.20	0.5 mL	18
Cd-based MOF	8.84 × 5.47	Propiophenone	10	134.18	100 μL	19
PCN-41	5.97 × 4.42	Benzaldehyde	8	106.12	Not available	20
LIFM-100	6.83 × 5.33	Pentanol	6	72.15	5 mL	21

4.2. General procedure for guest encapsulation into APF-40.

APF-40 single crystals immersed in DMF were transferred to an empty glass vial. Fresh acetone was added and kept for 1 min before the liquid was decanted, leaving the crystals behind. The process was repeated three times. Then, the crystals were further kept in acetone at 40 °C for 4 h to ensure a complete solvent exchange. For spironolactone, dexamethasone, betamethasone, fulvestrant, stearyl glycyrrhetinate, commercial ivermectin B_{1a} and doramectin, the guest powders were placed into 2 mL vials, and 1 mL of encapsulation solvent (*n*-heptane or a mixed solvent of *n*-heptane/acetone = 9:1, v/v) was added. After that, a drop of acetone containing a few APF-40 crystals was transferred into each guest solution vial. The vials were tightly capped and incubated at 40 °C. After several days, the guest-loaded APF-40 crystals were directly subjected to single-crystal X-ray diffraction analysis without any additional treatment. For samples recovered from HPLC separation: purified ivermectin B_{1a}, ivermectin B_{1b}, 3′-*O*-demethylivermectin B_{1a}, avermectin B_{1a}, 3,4-dihydroivermectin B_{1a} and ethyl-ivermectin, a small-volume (150 µL) Shim-vial™ H glass was used. The encapsulation experiments were performed using the abovementioned procedure but the solvent volumes were decreased to 25–100 µL. The encapsulation conditions for each guest are described in detail in Table S2.

Table S2. Experimental conditions of guest encapsulation into APF-40.

Guest No.	Name	Weight	Solvent	Temp.	Time
1	Spironolactone	1.7 mg	*n*-Heptane 1.0 mL	40 °C	7 days
2	Dexamethasone	1.8 mg	*n*-Heptane 1.0 mL	40 °C	7 days
3	Betamethasone	1.2 mg	*n*-Heptane 1.0 mL	40 °C	3 days
4	Stearyl glycyrrhetinate	4.5 mg	*n*-Heptane 0.9 mL<br>Acetone 0.1 mL	40 °C	5 days
5	Fulvestrant	2.4 mg	*n*-Heptane 0.9 mL<br>Acetone 0.1 mL	40 °C	3 days
6	Doramectin	4.0 mg	*n*-Heptane 0.9 mL<br>Acetone 0.1 mL	40 °C	7 days
7	Commercial ivermectin B_{1a}	1.8 mg	*n*-Heptane 1.0 mL<br>Acetone 0.1 mL	40 °C	3 days
8	Ivermectin B_{1a}<br>(Fraction A)	2.8 mg	*n*-Heptane 1.0 mL	40 °C	3 days
9	Ivermectin B_{1b}<br>(Fraction B)	90 µg	*n*-Heptane 90 µL<br>Acetone 10 µL	40 °C	3 days
10	3′-*O*-Demethylivermectin B_{1a}<br>(Fraction C)	25 µg	*n*-Heptane 22.5 µL<br>Acetone 2.5 µL	40 °C	3 days
11	Avermectin B_{1a}<br>(Fraction D)	100 µg	*n*-Heptane 90 µL<br>Acetone 10 µL	40 °C	3 days
12	3,4-Dihydroivermectin B_{1a}<br>(Fraction E)	100 µg	*n*-Heptane 90 µL<br>Acetone 10 µL	40 °C	3 days
13	Ethyl-ivermectin<br>(Fraction F)	< 50 µg	*n*-Heptane 45 µL<br>Acetone 5 µL	40 °C	3 days

4.3. Single crystal X-ray analysis of small molecules in APF-40.

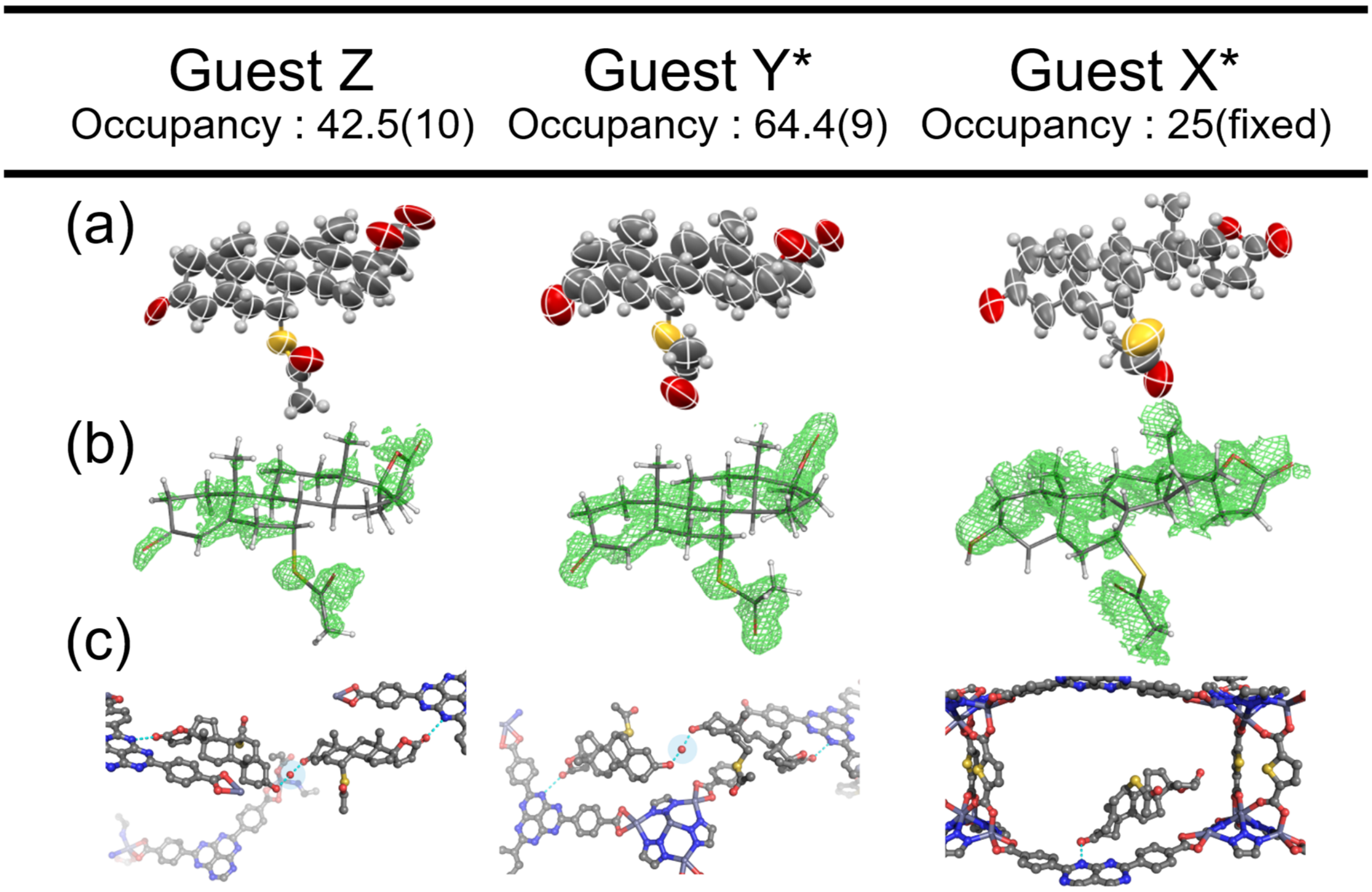

Fig. S13. Spironolactone encapsulated structure summary. a) Thermal ellipsoid models of spironolactone with 50% probability, b) 2Fo–Fc electron density maps (map thresholds for guests Z, Y and X are 0.70, 1.00 and 0.70 e/Å^3, respectively), c) interactions between HTCHAP^{3-}, water and spironolactone. Cyan dash lines represent hydrogen bond contacts. Atom coloring scheme: Zn – purple, C – grey, H – white, O – red, N – blue and S – yellow. The guests denoted by (*) occupied the same position in APF-40.

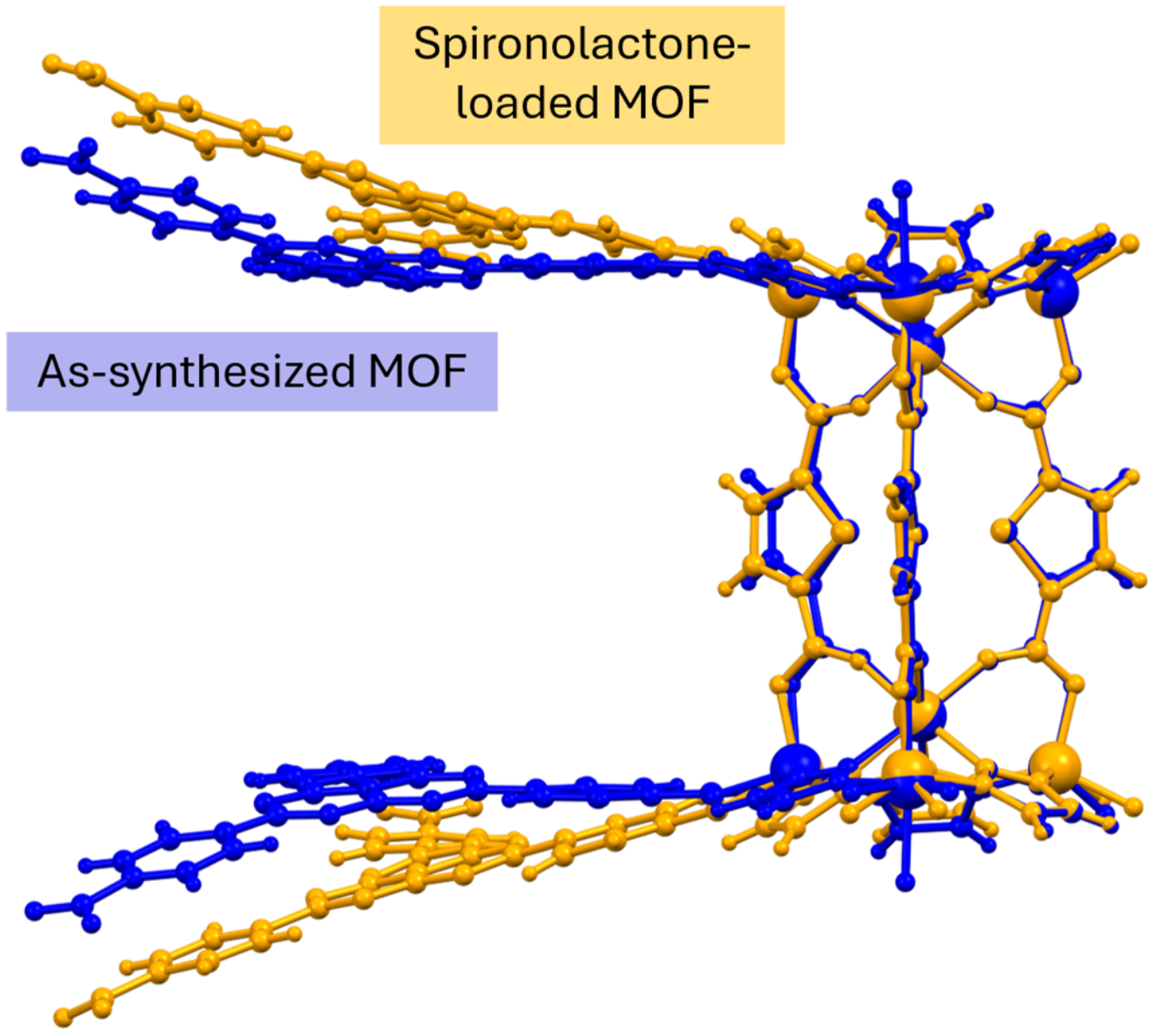

Fig. S14. Deformation of APF-40 after encapsulation of spironolactone. Overlay of crystal structures of the as-synthesized APF-40 (*blue*) and spironolactone-loaded APF-40 (*orange*).

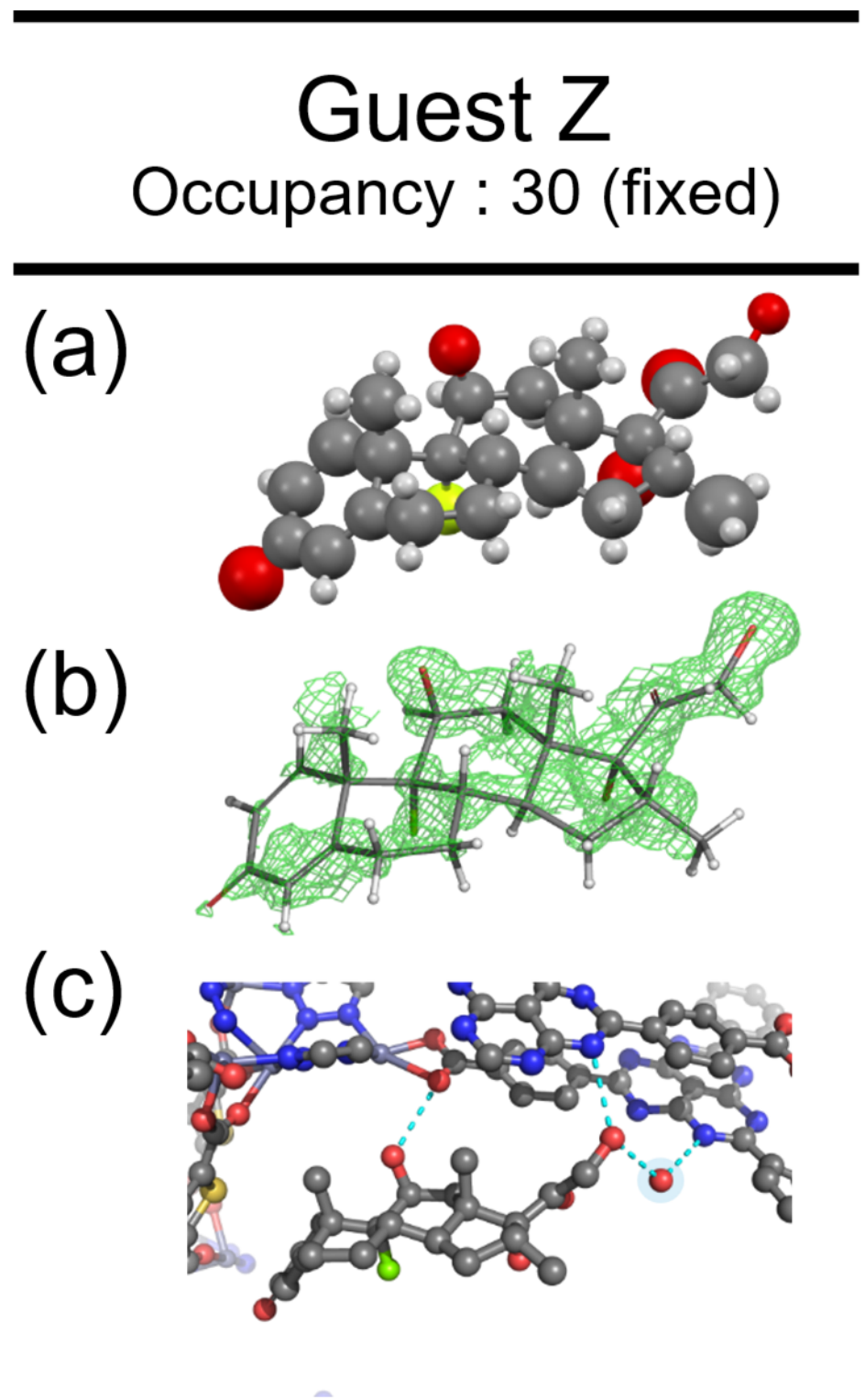

Fig. S15. Dexamethasone encapsulated structure summary. a) Isotropic model of dexamethasone with 50% probability, b) 2Fo–Fc electron density map (map threshold: 0.55 e/Å^3), c) interactions between HTCHAP^{3-}, water and dexamethasone. Cyan dash lines represent hydrogen bond contacts. Atom coloring scheme: Zn – purple, C – grey, H – white, O – red, N – blue, S – yellow and F – light green.

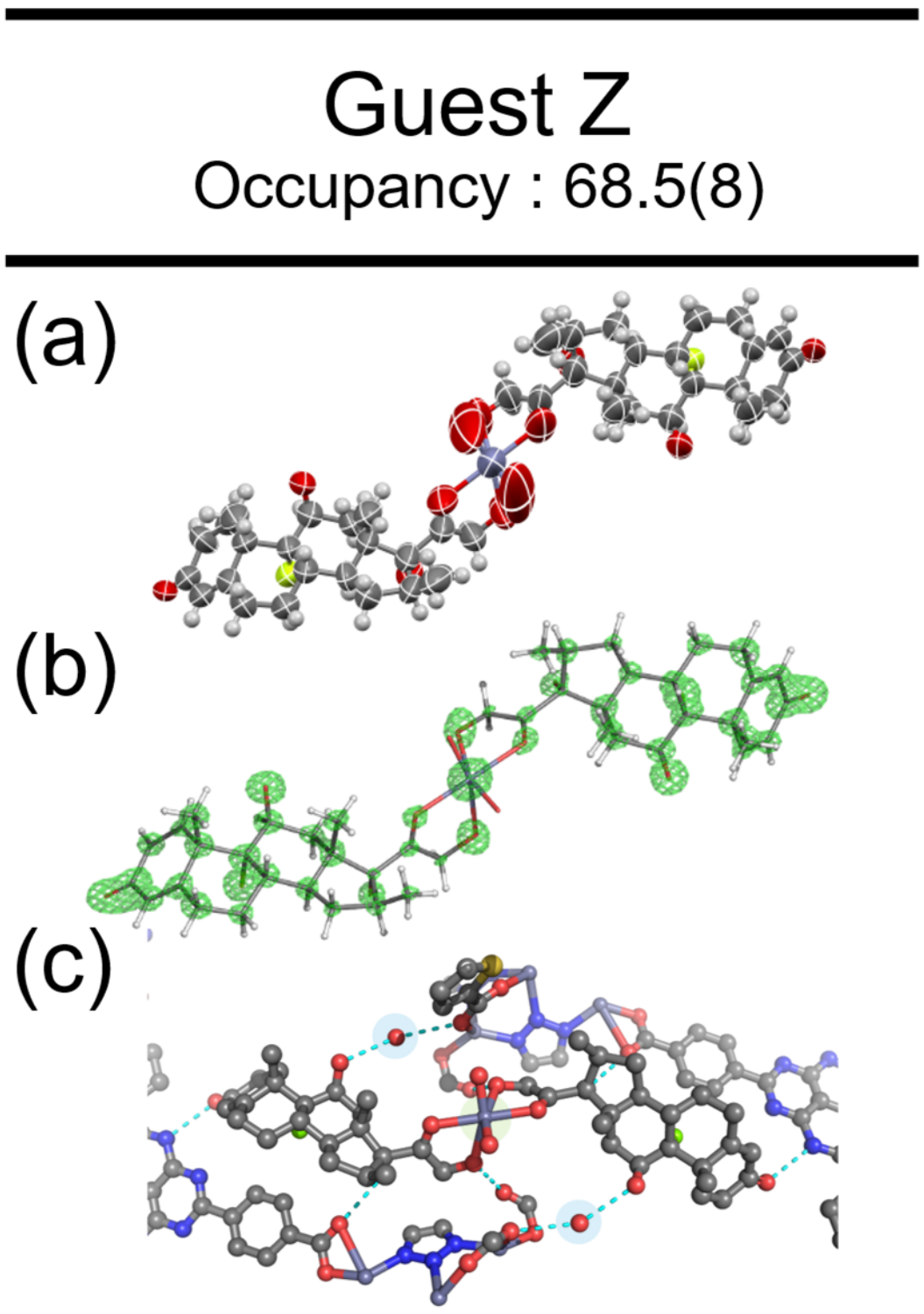

Fig. S16. Betamethasone encapsulated structure summary. a) Thermal ellipsoid model of $[Zn(betamethasone)_2(H_2O)_2]^{2+}$ with 50% probability, b) 2Fo–Fc electron density map (map threshold: 1.70 e/Å^3), c) interactions between Zn^{2+} ions, $HTCHAP^{3-}$, TDC^{2-}, water and betamethasone. Cyan dash lines represent hydrogen bond contacts. Atom coloring scheme: Zn – purple, C – grey, H – white, O – red, N – blue, S – yellow and F – light green.

4.4. Single crystal X-ray analysis of compounds containing flexible moieties in APF-40.

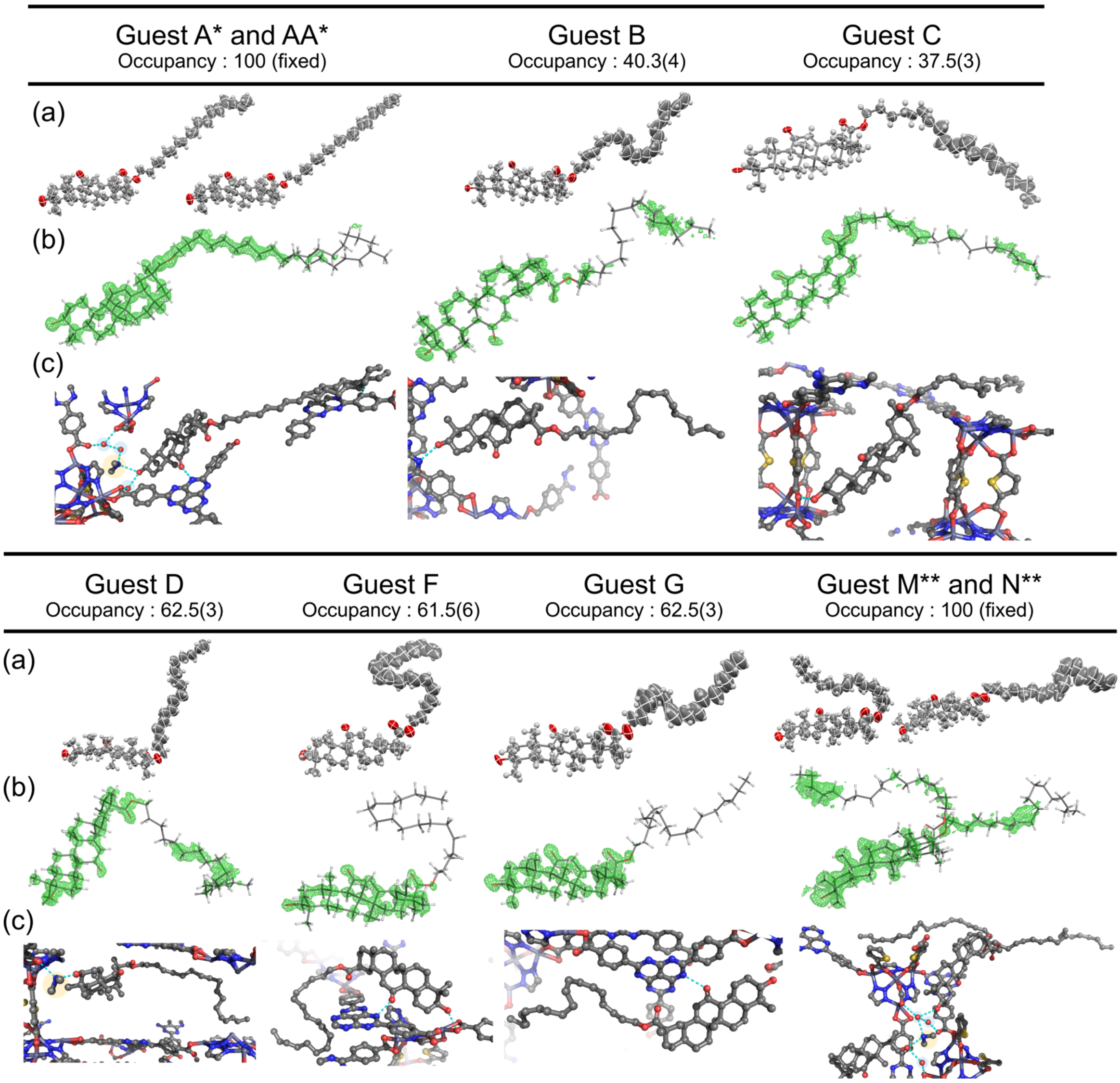

Fig. S17. Stearyl glycyrrhetinate encapsulated structure summary. a) Thermal ellipsoid models of stearyl glycyrrhetinate with 50% probability, b) 2Fo–Fc electron density maps (map thresholds for guests A, B, C, D, F, G and M are 1.20, 0.90, 1.00, 0.85, 1.35, 1.40 and 0.80 e/Å^3, respectively), c) interactions between $HTCHAP^{3-}$, water, $NH_2Me_2^+$ ions and stearyl glycyrrhetinate. Cyan dash lines represent hydrogen bond contacts. Atom coloring scheme: Zn – purple, C – grey, H – white, O – red, N – blue and S – yellow. The guests denoted by (*) and (**) occupied the same positions in APF-40.

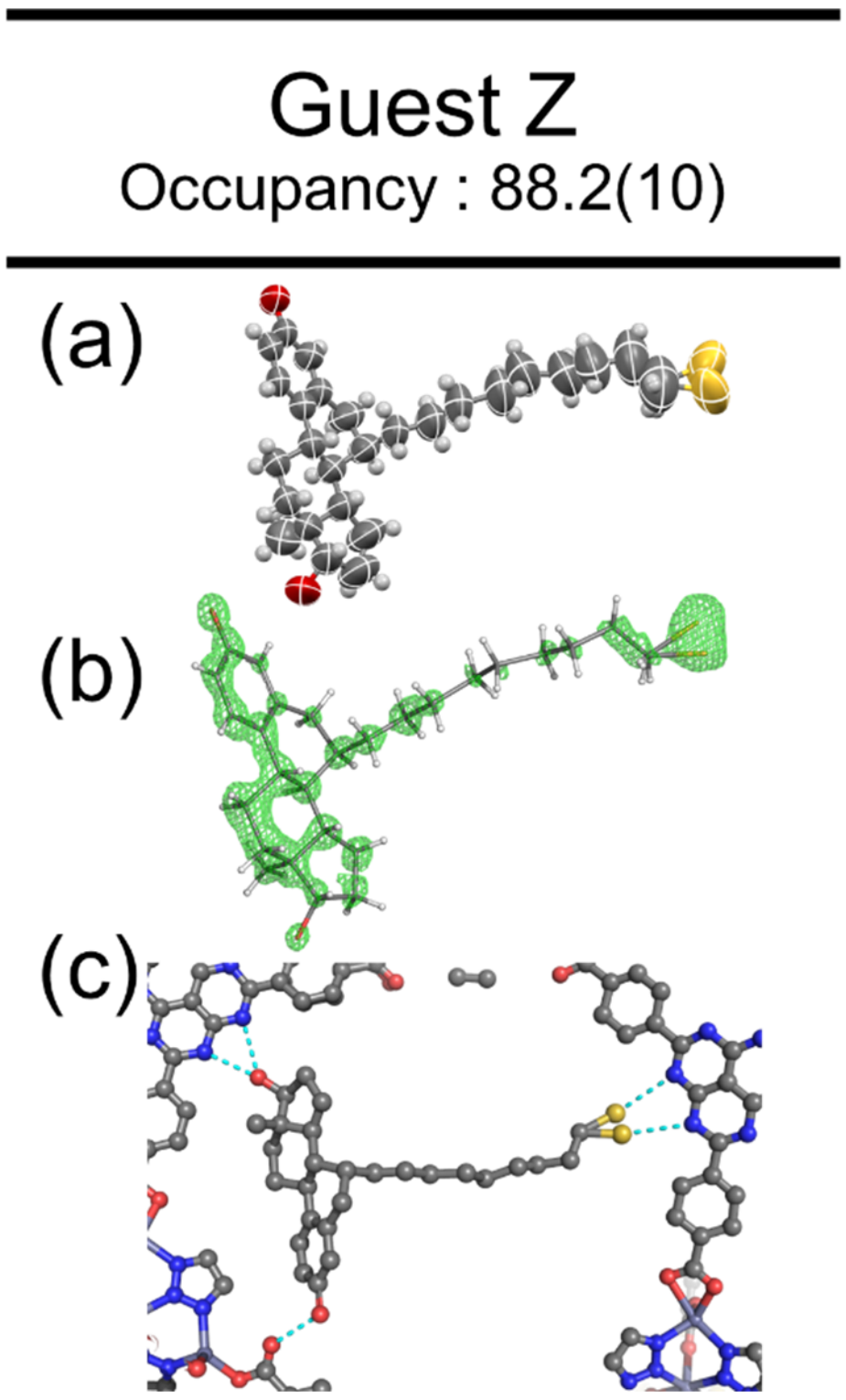

Fig. S18. Fulvestrant encapsulated structure summary. a) Thermal ellipsoid model of fulvestrant with 50% probability, b) 2Fo–Fc electron density map (map threshold: 1.45 e/Å^3), c) interactions between HTCHAP^{3-} and fulvestrant. Cyan dash lines represent hydrogen bond contacts. Atom coloring scheme: Zn – purple, C – grey, H – white, O – red, N – blue and S – yellow.

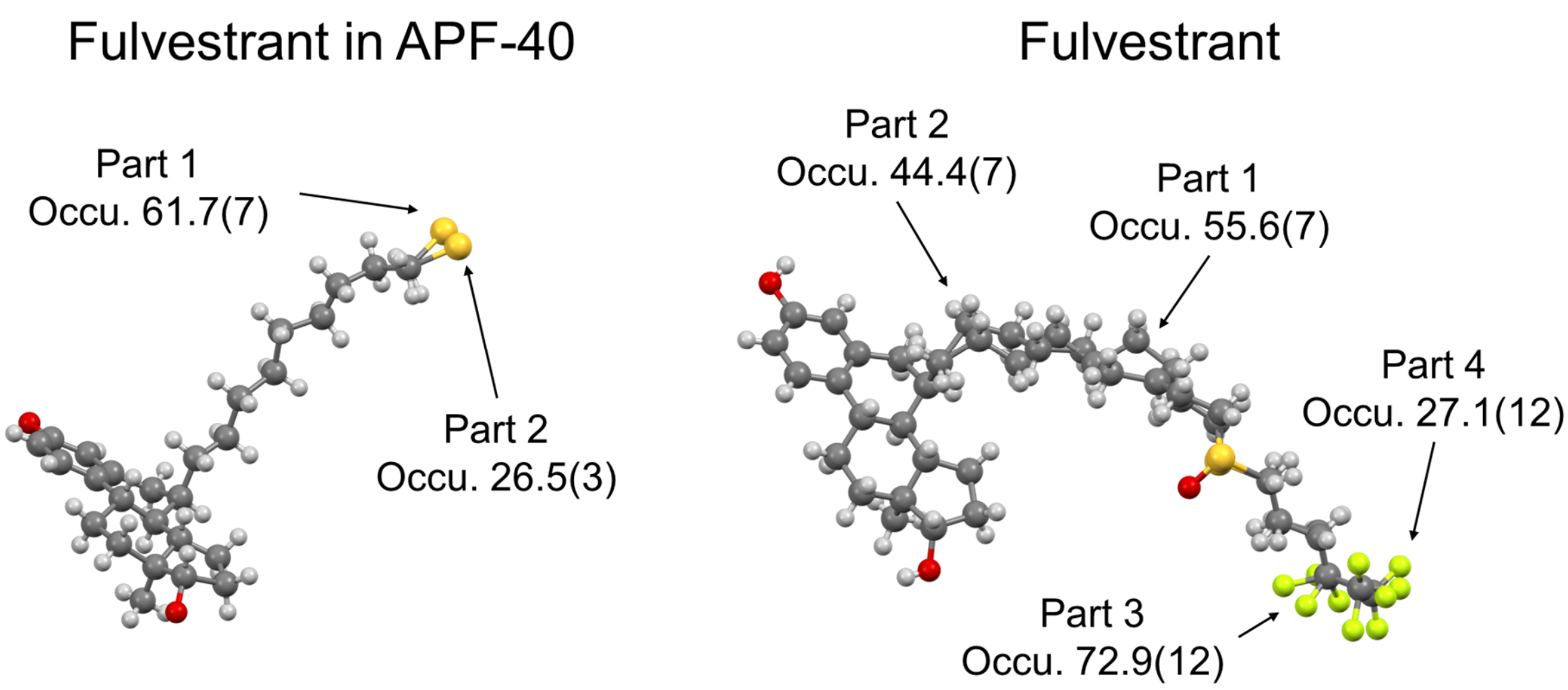

Fig. S19. Structural comparison between fulvestrant inside APF-40 and crystallized pure sample. The disorder model of fulvestrant shown as ball and stick model. Atom coloring scheme: C – grey, H – white, O – red, S – yellow and F – light green.

4.5. Characterization of fulvestrant extracted from the guest-loaded APF-40 crystals.

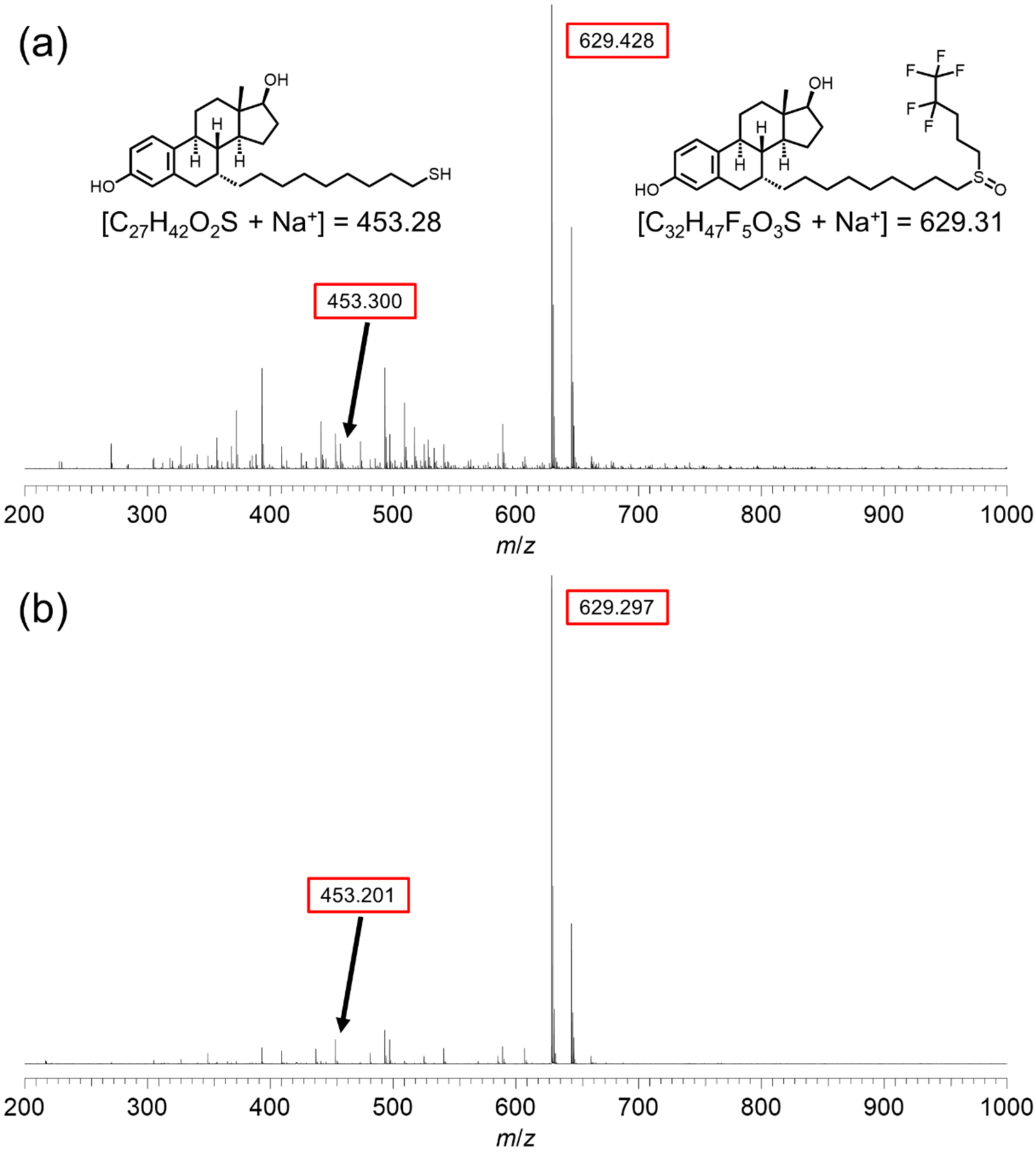

Fig. S20. Mass spectra of fulvestrant in methanol. (a) The sample extracted from the guest-loaded APF-40 crystals, which were prepared using the same encapsulation method as the crystalline sponge analysis. (b) HPLC-purified fulvestrant.

4.6. Single crystal X-ray analysis of bRo5 compounds in APF-40.

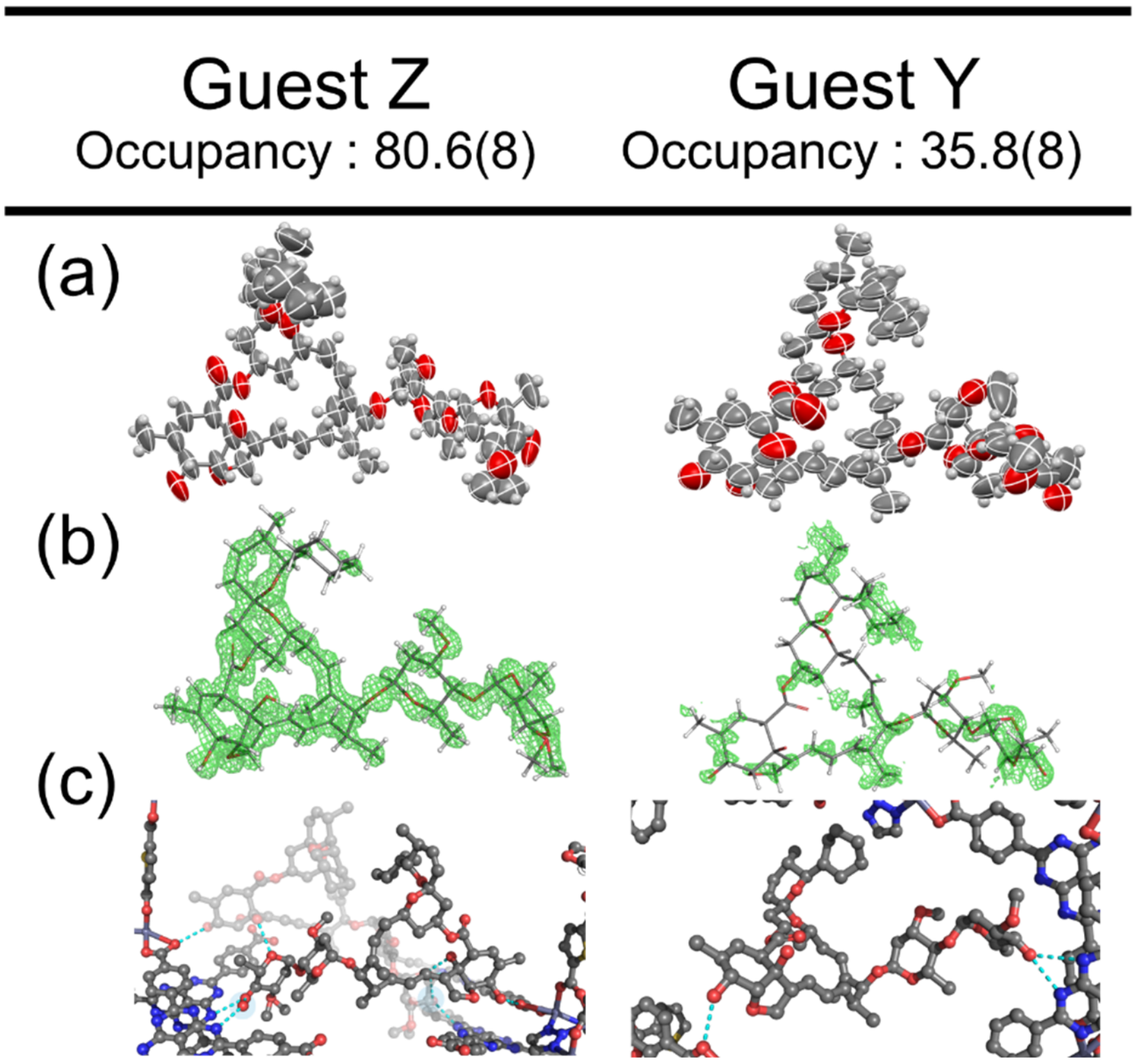

Fig. S21. Doramectin encapsulated structure summary. a) Thermal ellipsoid models of doramectin with 50% probability, b) 2Fo–Fc electron density maps (map threshold for guests Z and Y are 0.85 and 0.65 e/Å^3, respectively), c) interactions between HTCHAP^{3-}, water and doramectin. Cyan dash lines represent hydrogen bond contacts. Atom coloring scheme: Zn – purple, C – grey, H – white, O – red, N – blue and S – yellow.

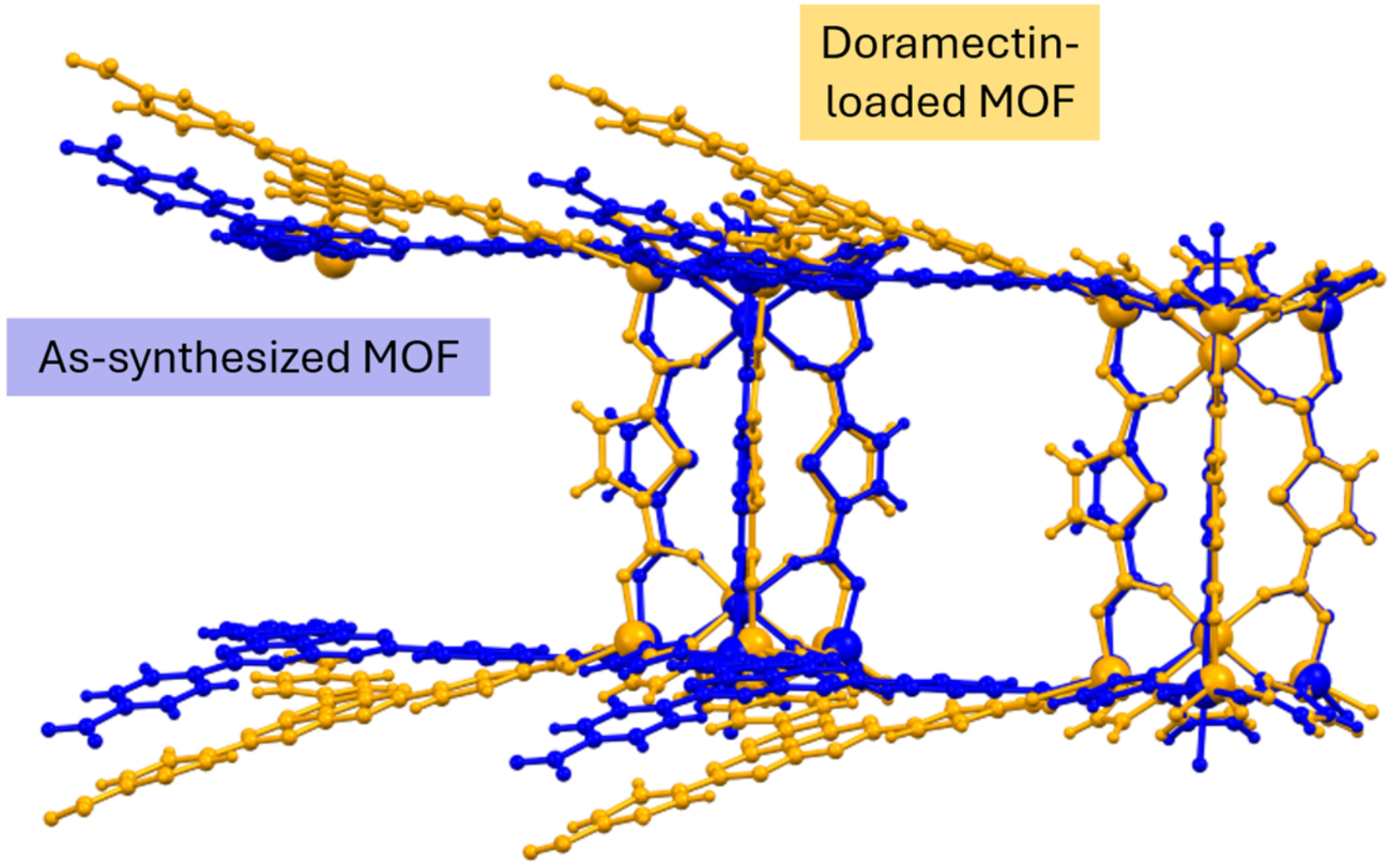

Fig. S22. Deformation of APF-40 after encapsulation of doramectin. Overlay of crystal structures of the as-synthesized APF-40 (*blue*) and doramectin-loaded APF-40 (*orange*).

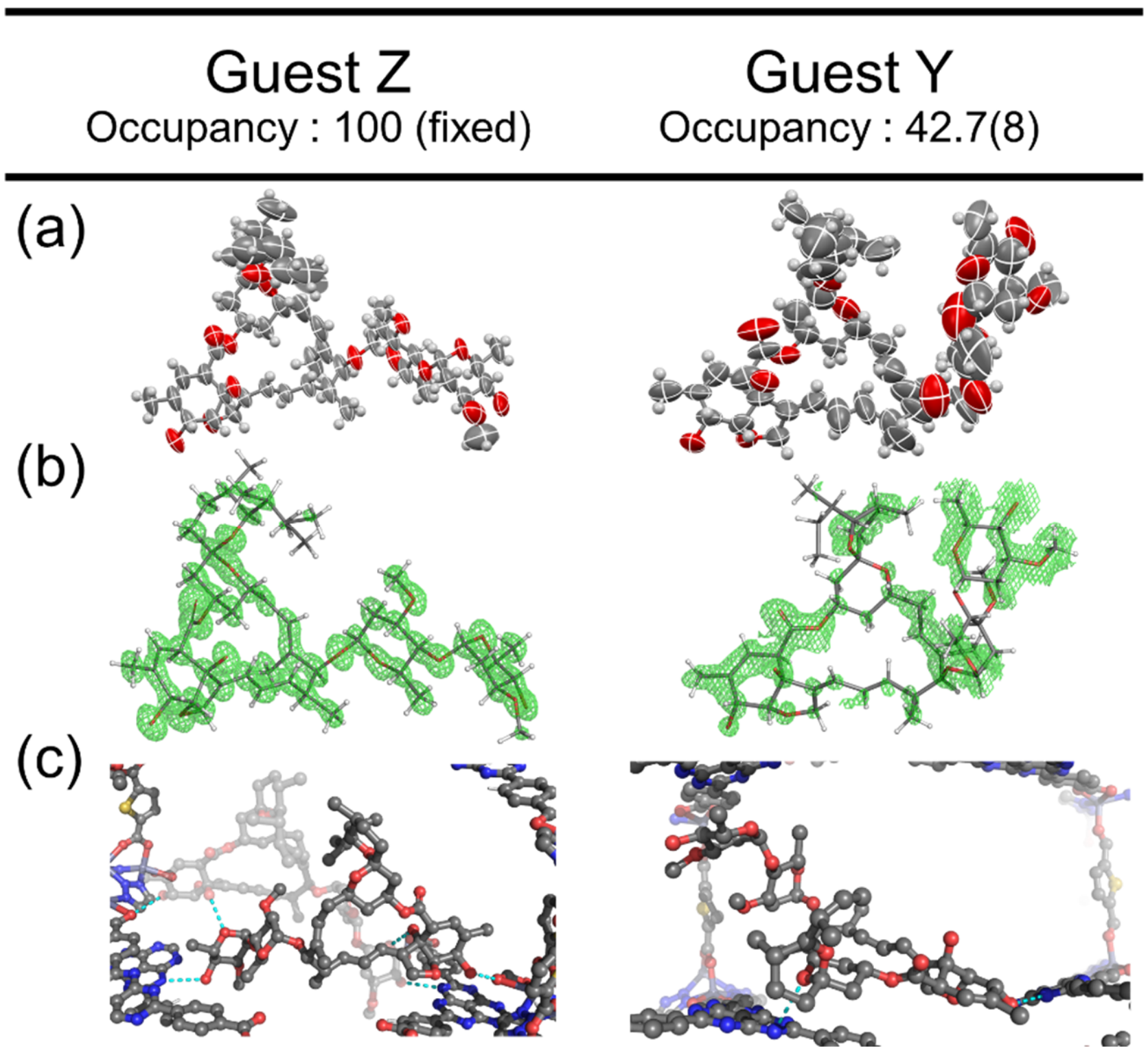

Fig. S23. Ivermectin B_{1a} encapsulated structure summary. a) Thermal ellipsoid models of ivermectin B_{1a} with 50% probability, b) 2Fo–Fc electron density maps (map threshold for guests Z and Y are 1.40 and 0.62 e/Å^3, respectively), c) interactions between $HTCHAP^{3-}$ and ivermectin B_{1a}. Cyan dash lines represent hydrogen bond contacts. Atom coloring scheme: Zn – purple, C – grey, H – white, O – red, N – blue and S – yellow.

5. HPLC separation and characterization of six ivermectin derivatives from the commercial reagent.

5.1. Experimental scheme of HPLC separation.

Ivermectin B_{1a} was dissolved in methanol/H_2O (90/10, v/v) and separated with HPLC [COSMOSIL Cholester (ϕ 20 mm × 250 mm) using 90% aqueous methanol as eluent; flow rate 5 mL/min; detection UV 215 nm] in 4 batches (28.7, 29.7, 28.4 and 28.9 mg, in total 115.7 mg) to yield ivermectin B_{1a} (> 100 mg in total, $39.5 < t_R < 40.5$ min) and ivermectin B_{1b} (0.7 mg in total, $t_R = 36.3$ min), as well as mixed fractions 1 and 3 ($t_R = 29.6$, 54.5 min) (Fig. S24). Then, mixed fraction 1 was further separated by HPLC [COSMOSIL Cholester (ϕ 20 mm × 250 mm) using acetonitrile/H_2O (75/25, v/v); flow rate 5 mL/min; detection UV 254 nm] to yield 3′-*O*-demethylivermectin B_{1a} (< 0.1 mg, $t_R = 49.3$ min) and mixed fraction 2 ($t_R = 53.5$ min) (Fig. S25). Mixed fraction 2 was further separated by HPLC [COSMOSIL 5C_{18}-MS-II (ϕ 20 mm × 250 mm) using acetonitrile/H_2O (80/20, v/v); flow rate 5 mL/min; detection UV 254 nm] to yield avermectin B_{1a} (0.4 mg, $t_R = 52.6$ min) (Fig. S26). Mixed fraction 3 was further separated by HPLC [COSMOSIL Cholester (ϕ 20 mm × 250 mm) using acetonitrile/H_2O (85/15, v/v); flow rate 5 mL/min; detection UV 254 nm] to yield 3,4-dihydroivermectin B_{1a} (< 0.1 mg, $t_R = 73.1$ min) and mixed fraction 4 ($t_R = 78.3$ min) (Fig. S27). Mixed fraction 4 was further separated by HPLC [COSMOSIL 5C_{18}-MS-II (ϕ 20 mm × 250 mm) using methanol/H_2O (90/10, v/v); flow rate 5 mL/min; detection UV 254 nm] to yield mixed fraction 5 ($t_R = 81.1$ min) (Fig. S28). Finally, mixed fraction 5 was further separated by HPLC [COSMOSIL 5C_{18}-MS-II (ϕ 20 mm × 250 mm) using acetonitrile/H_2O (93/7, v/v); flow rate 5 mL/min; detection UV 254 nm] to yield ethyl-ivermectin (< 0.1 mg, $t_R = 66.1$ min) (Fig. S29). The main HPLC peaks isolated after separations were analyzed by ^1H NMR (Fig. S31, Fig. S33, Fig. S36, Fig. S39, Fig. S42, Fig. S45 and Fig. S47 to Fig. S50) to check fraction composition with the exception of mixed fraction 5. If the ^1H NMR analysis indicated the presence of two or more compounds, HPLC separations were continued until only one pure component remained.

5.2. HPLC charts.

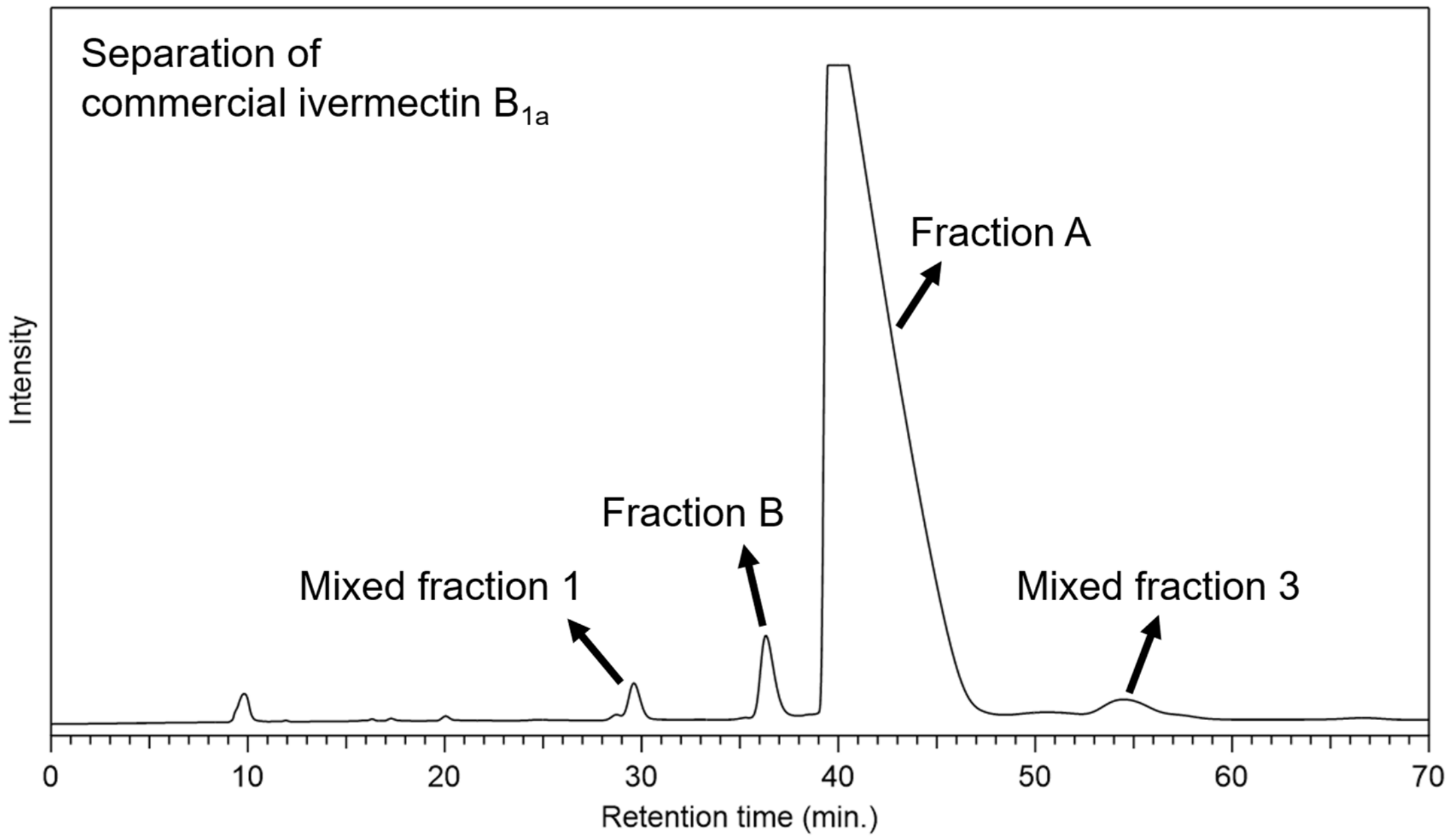

Fig. S24. HPLC chromatogram of commercial ivermectin B_{1a} sample. COSMOSIL Cholester (ϕ 20 mm × 250 mm) and methanol/water (90/10, v/v) were used for column and solvent, respectively.

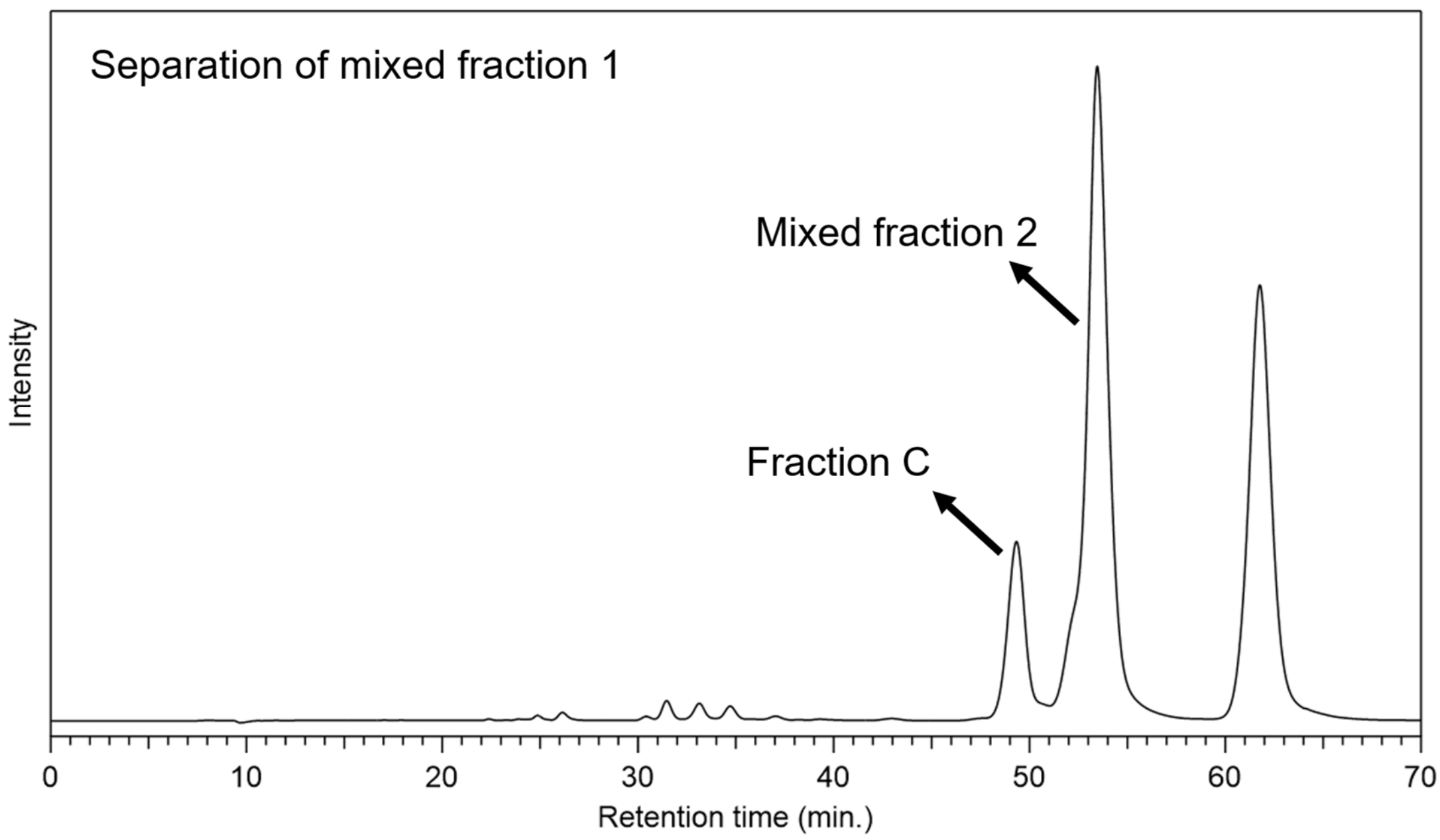

Fig. S25. HPLC chromatogram of mixed fraction 1. COSMOSIL Cholester (ϕ 20 mm × 250 mm) and acetonitrile/water (75/25, v/v) were used for column and solvent, respectively.

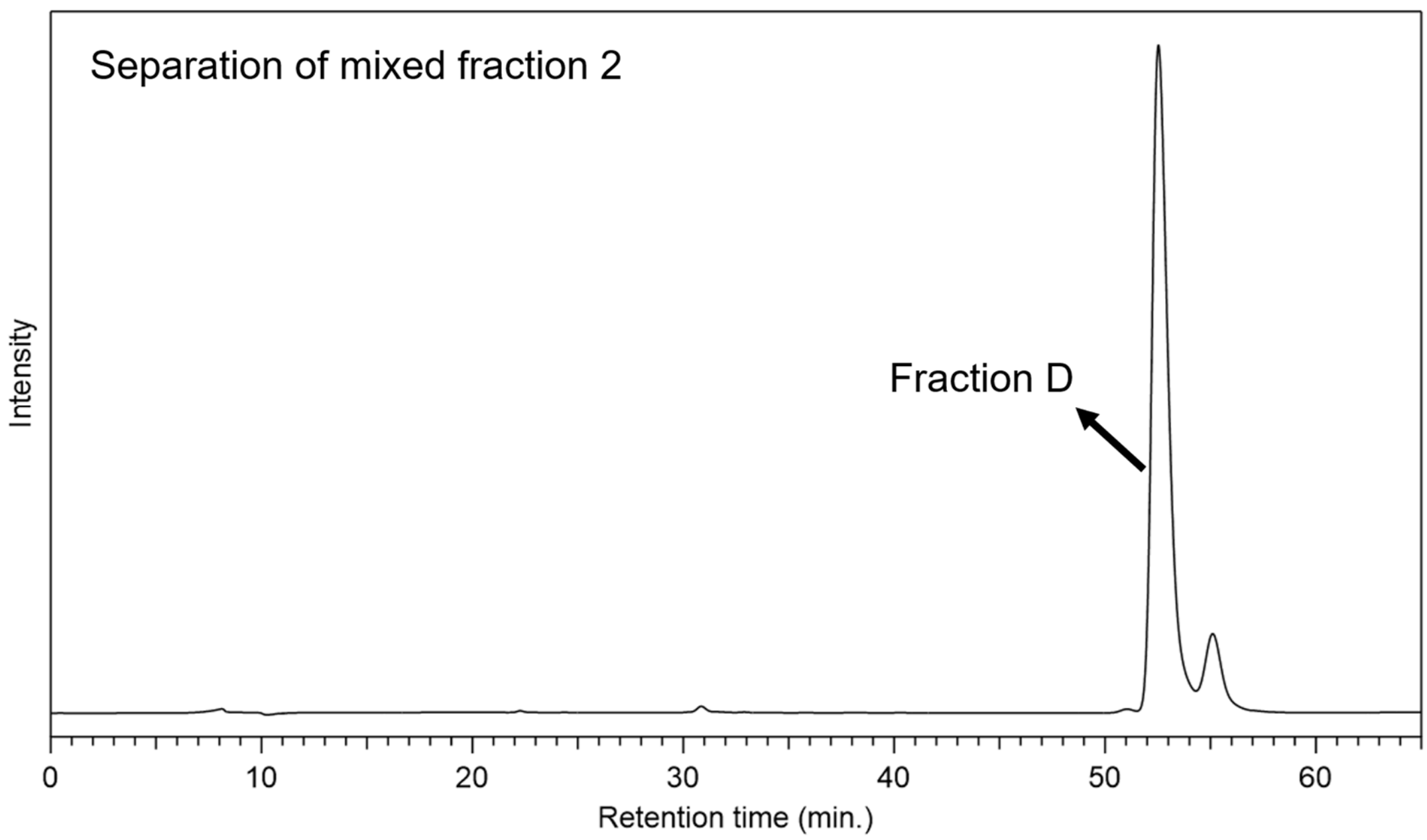

Fig. S26. HPLC chromatogram of mixed fraction 2. COSMOSIL $5C_{18}$-MS-II (ϕ 20 mm × 250 mm) and acetonitrile/water (80/20, v/v) were used for column and solvent, respectively.

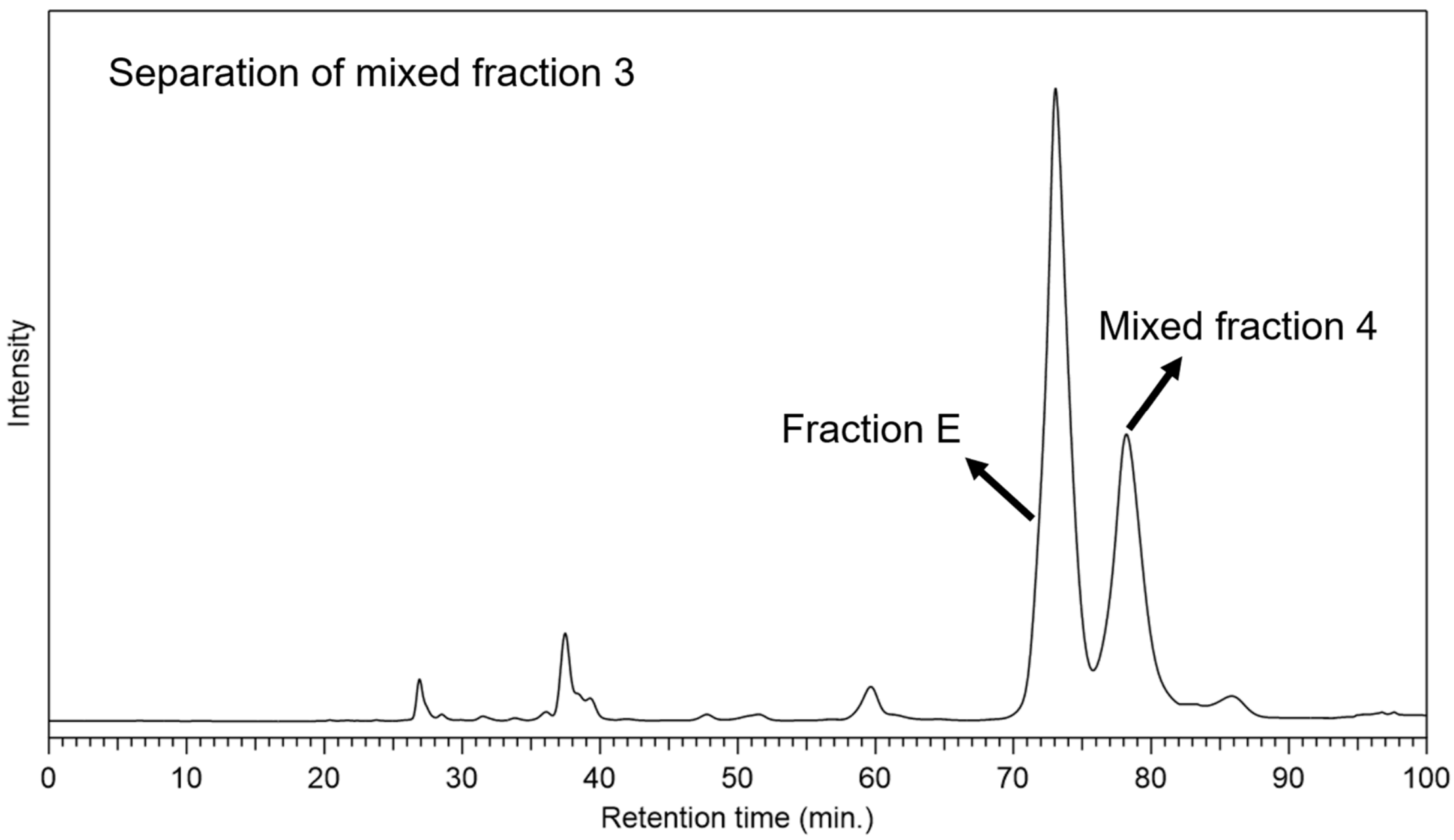

Fig. S27. HPLC chromatogram of mixed fraction 3. COSMOSIL Cholester (ϕ 20 mm × 250 mm) and acetonitrile/water (85/15, v/v) were used for column and solvent, respectively.

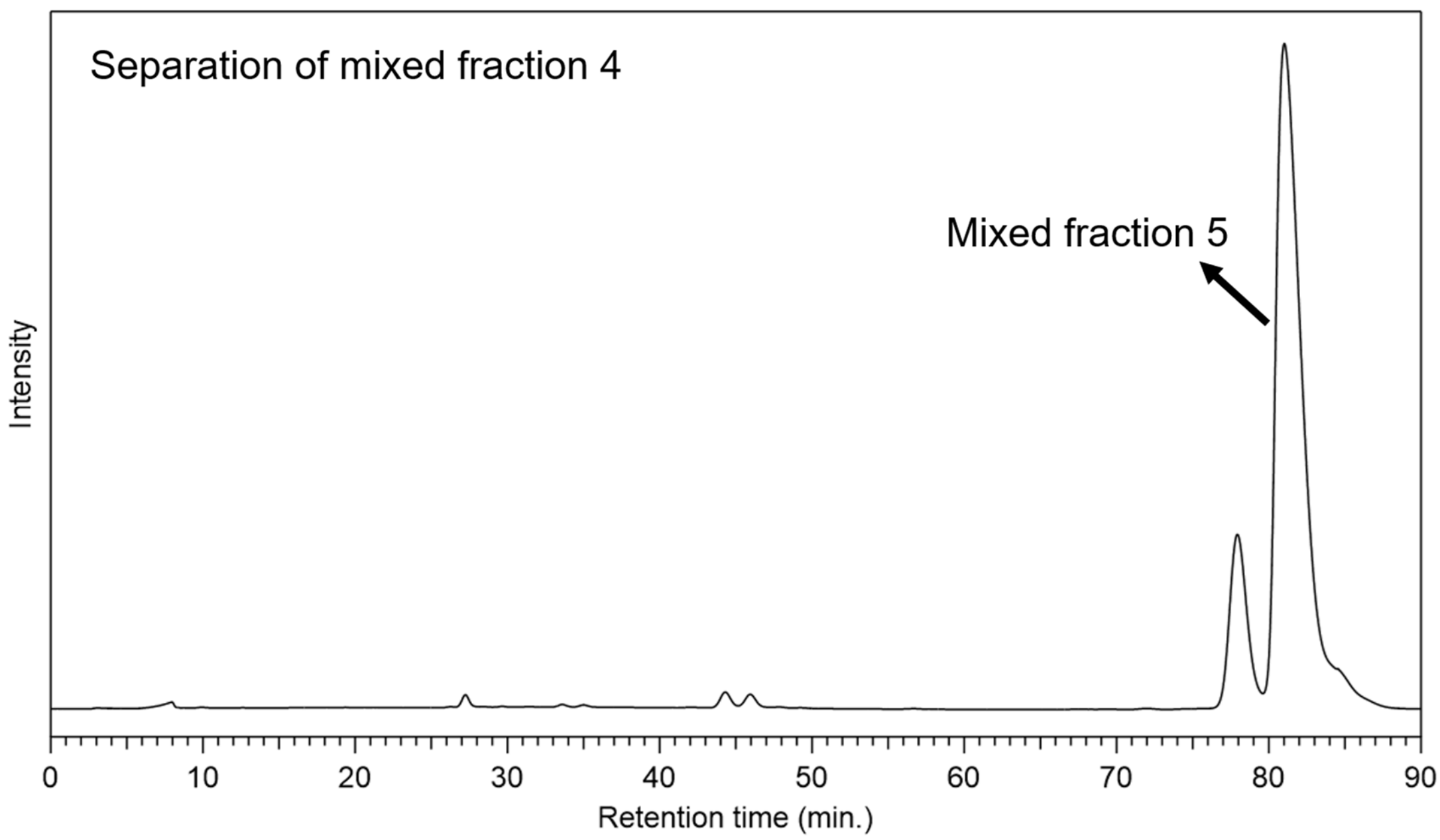

Fig. S28. HPLC chromatogram of mixed fraction 4. COSMOSIL 5C18-MS-II (ϕ 20 mm × 250 mm) and methanol/water (90/10, v/v) were used for column and solvent, respectively.

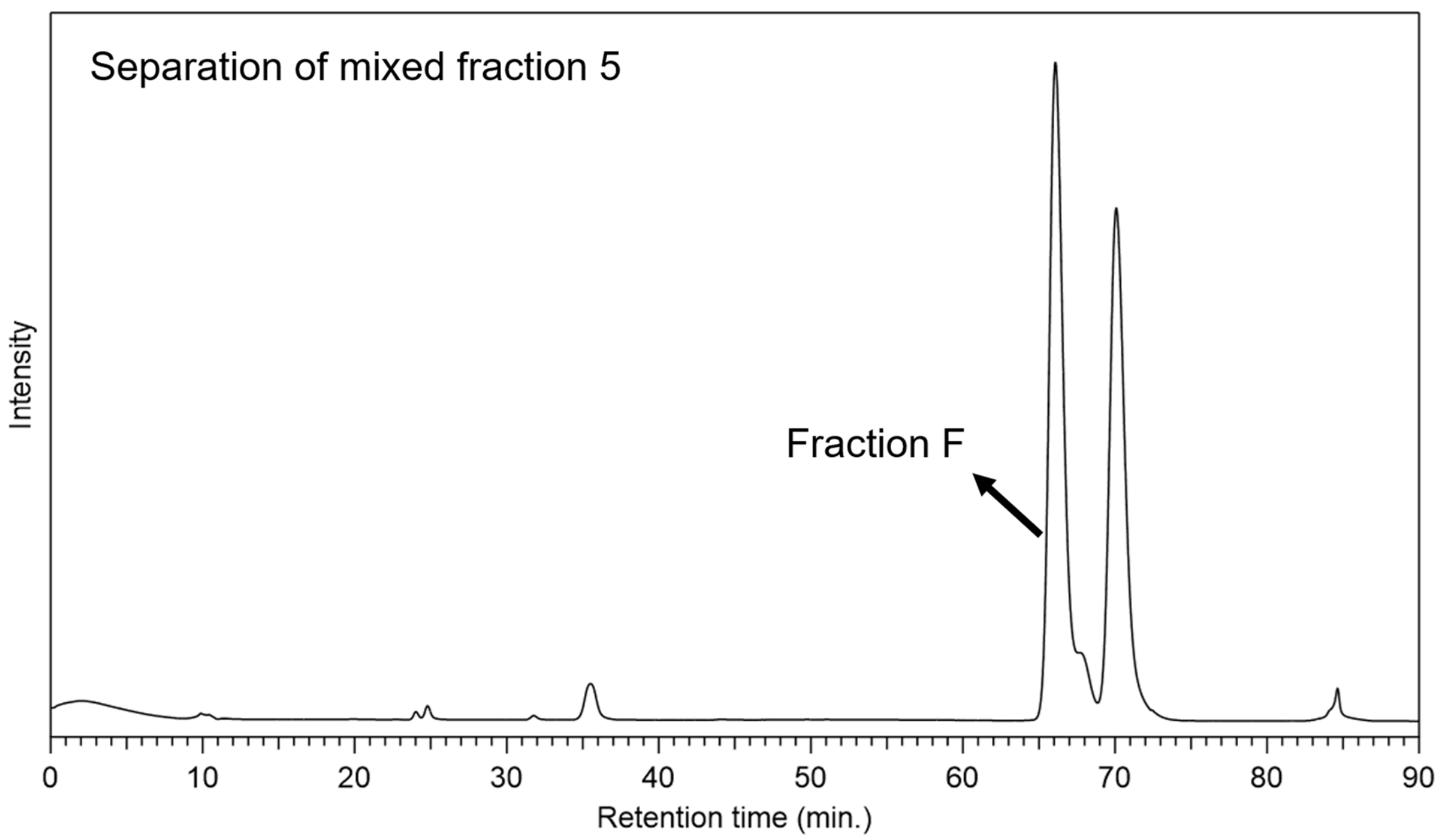

Fig. S29. HPLC chromatogram of mixed fraction 5. COSMOSIL 5C18-MS-II (ϕ 20 mm × 250 mm) and acetonitrile/water (93/7, v/v) were used for column and solvent, respectively.

5.3. Characterization of HPLC-separated ivermectin derivatives by MS and ^{1}H NMR.

Table S3. Molecular structures of ivermectin derivatives from fractions A, B, C, D, E and F.

Fraction	Compound name	Weight / mg	Chemical formula	Structure difference relative to ivermectin B_{1a}	Chemical structure
**A**	Ivermectin B_{1a}	> 100	$C_{48}H_{74}O_{14}$	-	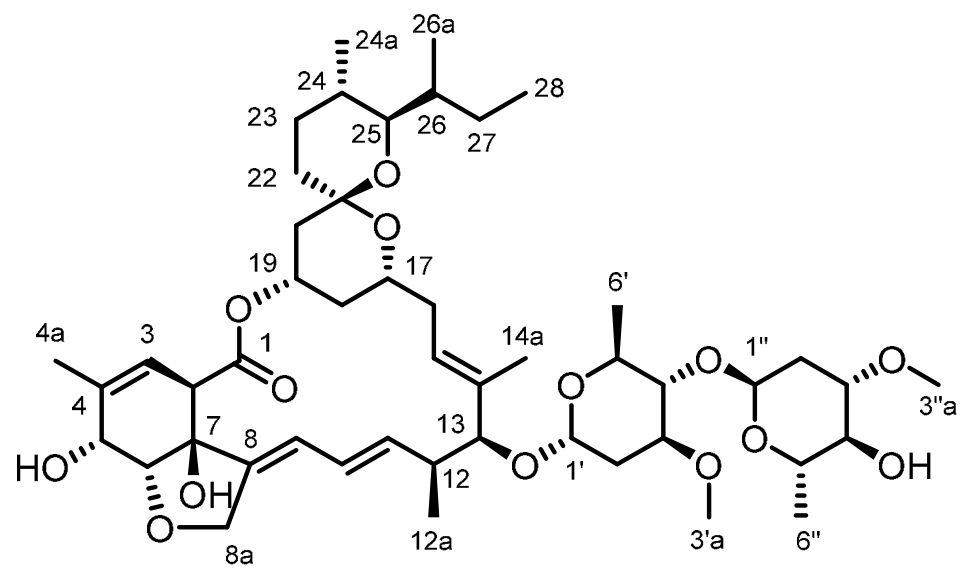
**B**	Ivermectin B_{1b}	0.7	$C_{47}H_{72}O_{14}$	Substituent on C25	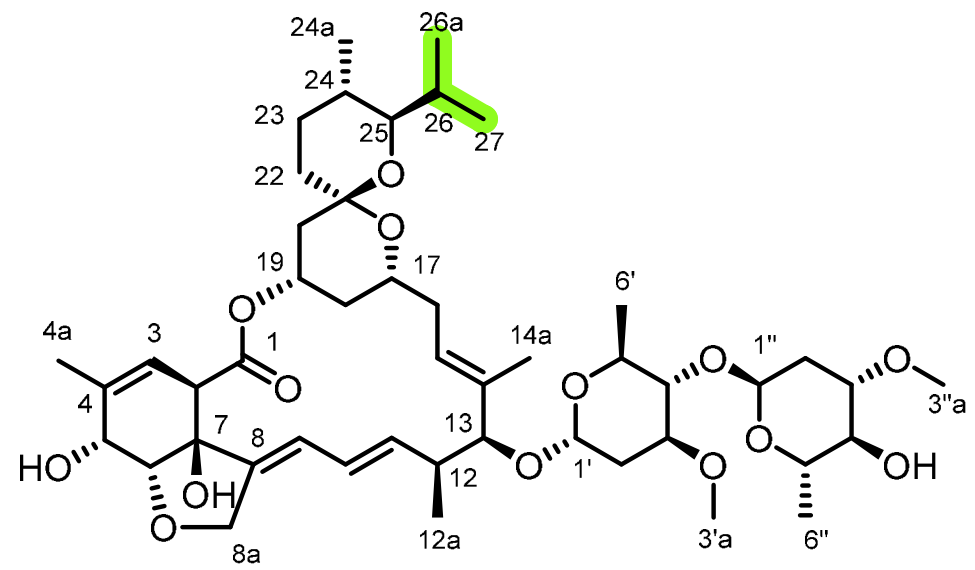
**C**	3′-*O*-Demethyl ivermectin B_{1a}	< 0.1	$C_{47}H_{72}O_{14}$	Demethylation of methoxy group on C3′	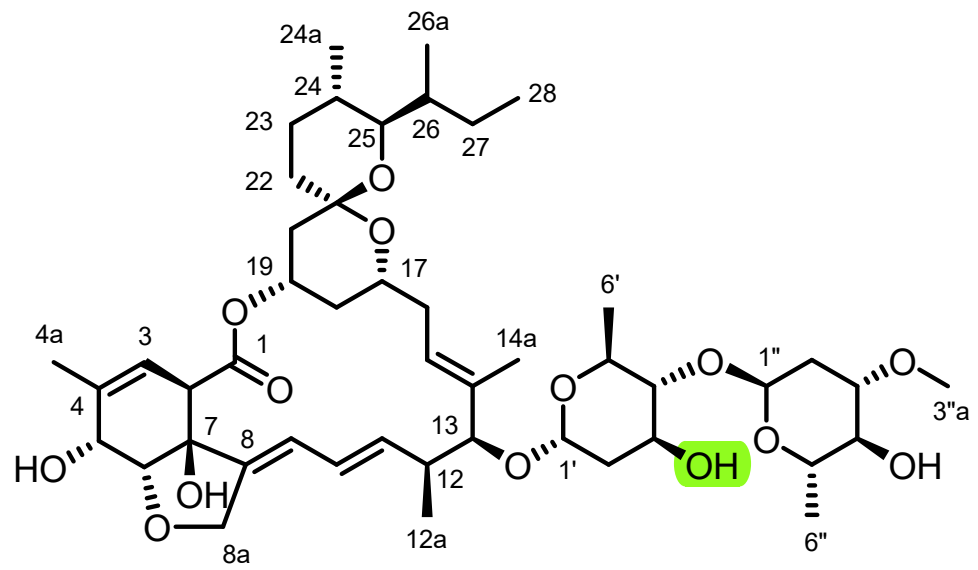

Fraction	Compound name	Weight / mg	Chemical formula	Structure difference relative to ivermectin B_{1a}	Chemical structure
**D**	22,23-Dedihydro ivermectin B_{1a} (Avermectin B_{1a})	0.4	$C_{48}H_{72}O_{14}$	Dehydrogenation of C22-C23	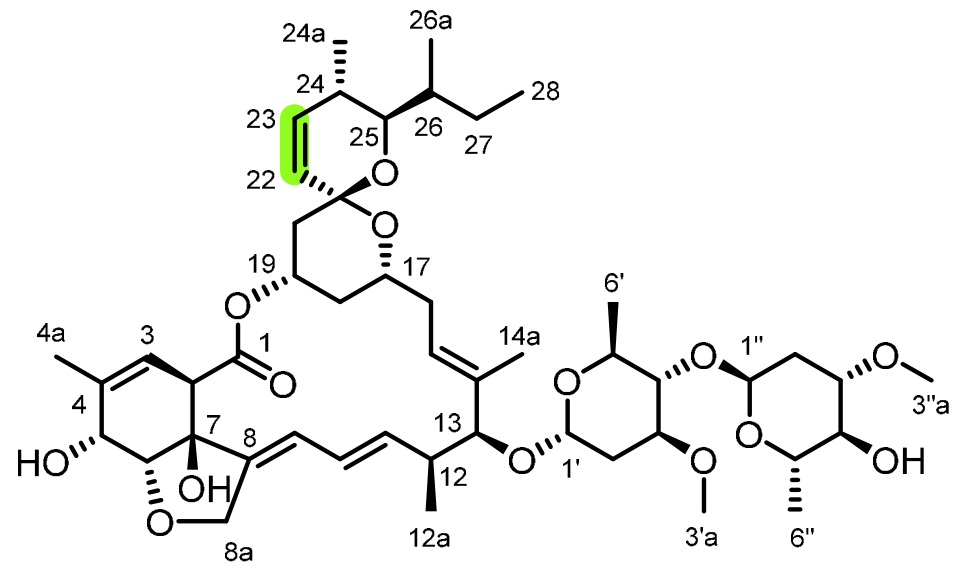
**E**	3,4-Dihydroivermectin B_{1a}	< 0.1	$C_{48}H_{76}O_{14}$	Hydrogenation of C3-C4	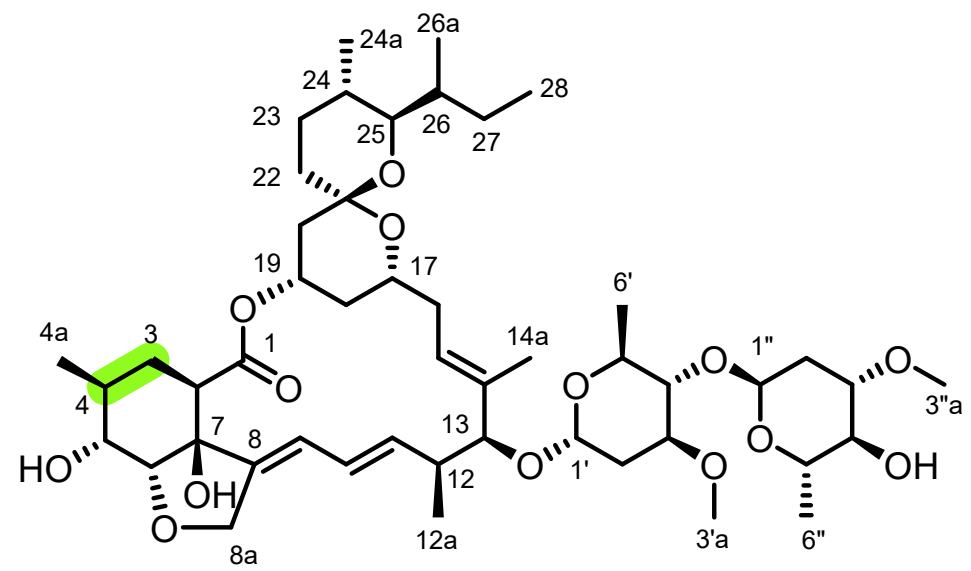
**F**	Ethyl-ivermectin	< 0.1	$C_{49}H_{76}O_{14}$	Ethyl group on C24	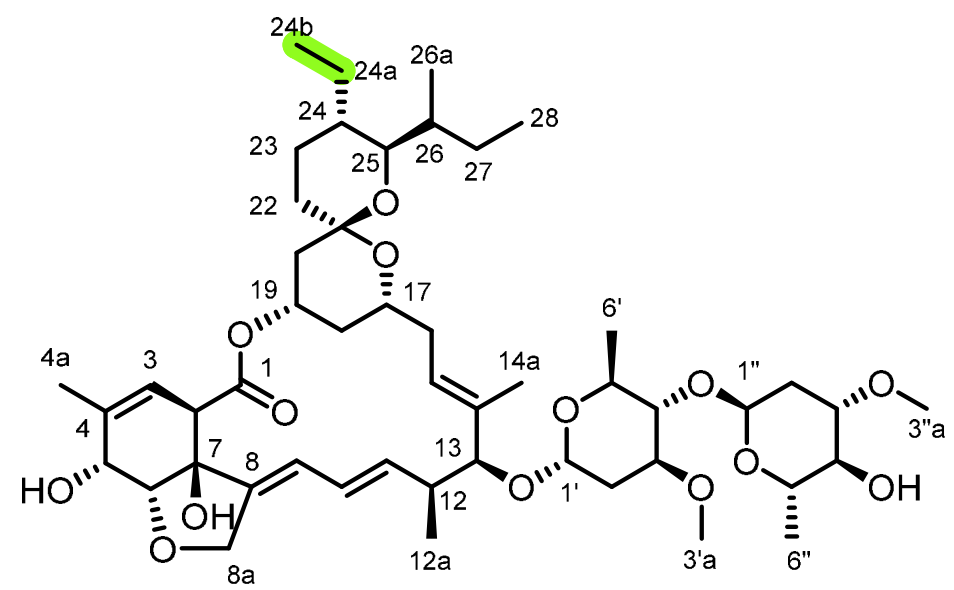

5.4. ^{1}H NMR signals of fraction A, B, C, D, E and F.

Table S4. ^{1}H NMR signals of fractions A, B, C, D, E and F in acetonitrile-d_3. The signals were assigned by comparison with the literature[22]. *Green* indicate signals with ambiguous assignment.

Position	Ivermectin B_{1a} (Fraction A)	Ivermectin B_{1b} (Fraction B)	3′-*O*-Demethyl ivermectin B_{1a} (Fraction C)	Avermectin B_{1a} (Fraction D)	3,4-Dihydro ivermectin B_{1a} (Fraction E)	Ethyl-ivermectin (Fraction F)
1	-	-	-	-	-	-
2	3.14, m	3.14, m	3.14, m	3.14, m	overlapped	3.14, m
3	5.38, brs	5.38, brs	5.38, brs	5.38, brs	overlapped	5.38, brs
4					overlapped	
4a	1.78, brs	1.78, brs	1.78, brs	1.77, brs	0.99, d (6.5)	1.78, brs
5	4.16, brd (5.4)	4.17, brs	4.16, brd (5.4)	4.16, m	overlapped	4.16, m
6	3.74, d (5.7)	3.75, d (5.7)	3.74, d (5.7)	3.74, d (5.7)	3.59, d (3.7)	3.74, d (5.7)
7	-	-	-	-	-	-
8	-	-	-	-	-	-
8a	4.57, brd (1.7)	4.57, brs	4.56, brd (1.7)	4.57, brd (1.7)	4.50, m	4.56, brd (1.7)
9	5.81, brd (11.1)	5.79, brd (11.1)	5.80, brd (11.1)	5.82, brd (11.1)	5.70, dd (11.1, 2.0)	5.80, brd (11.1)
10	5.87, dd (14.5, 11.1)	5.86, dd (14.5, 11.1)	5.86, dd (14.5, 11.1)	5.87, dd (14.5, 11.1)	5.82, dd (14.5, 11.1)	5.87, dd (14.5, 11.1)
11	5.77, dd (14.5, 9.4)	5.74, dd (14.5, 9.7)	5.73, dd (14.5, 9.4)	5.78, dd (14.5, 9.4)	5.71, dd (14.5, 9.4)	5.76, dd (14.5, 9.4)
12	2.60, dq (9.4, 6.8)	2.60, dq (9.7, 6.8)	2.60, m	2.60, dq (9.4, 6.8)	2.57, dq (9.4, 6.8)	2.61, dq (9.4, 6.8)
12a	1.12, d (6.8)	1.12, d (6.8)	1.12, d (6.8)	1.12, d (6.8)	1.11, d (6.8)	1.12, d (6.8)
13	3.92, brs	3.92, brs	3.92, brs	3.92, brs	3.91, brs	3.92, brs
14	-	-	-	-	-	-
14a	1.50, brs	1.49, brs	1.49, brs	1.49, brs	1.49, brs	1.49, brs
15	5.18, brd (9.9)	5.18, brd (9.9)	5.15, brd (9.9)	5.19, brt (7.7)	5.14, m	5.17, brd (9.9)
16	2.23, m	2.23, m	2.23, m	2.23, m	overlapped	overlapped
	2.19, m	2.19, m	2.19, m	2.19, m	overlapped	overlapped
17	3.70, m	3.70, m	3.70, m	3.89, m	overlapped	3.70, m
18	1.87, m	1.87, m	1.87, m	1.89, m	1.78, m	1.87, m
	0.75, m	0.75, m	0.75, m	0.78, ddd (11.9, 11.9, 11.9)	0.75, m	0.75, m
19	5.00, m	5.03, m	4.98, m	5.03, m	5.15, m	4.98, m
20	2.07, ddd (12.2, 5.1, 1.4)	2.07, ddd (12.2, 5.1, 1.4)	2.07, m	2.05, ddd (12.2, 5.1, 1.4)	overlapped	2.07, ddd (12.2, 5.1, 1.4)
	1.22, m	1.22, m	1.22, m	1.22, m	overlapped	1.22, m

Position	Ivermectin B_{1a} (Fraction A)	Ivermectin B_{1b} (Fraction B)	3′-*O*-Demethyl ivermectin B_{1a} (Fraction C)	Avermectin B_{1a} (Fraction D)	3,4-Dihydro ivermectin B_{1a} (Fraction E)	Ethyl-ivermectin (Fraction F)
21	-	-	-	-	-	-
22	1.57, m	1.57, m	1.57, m	5.72, dd (9.7, 1.7)	1.57, m	overlapped
	1.48, m	1.48, m	1.48, m		1.48, m	overlapped
23	1.48, m	1.48, m	1.48, m	5.52, dd (9.7, 2.6)	1.48, m	overlapped
24	1.45, m	1.45, m	1.45, m	2.24, m	1.45, m	overlapped
24a	0.78, d (5.4)	0.79, d (5.4)	0.78, d (5.4)	0.89, d (7.1)	0.78, d (5.4)	overlapped
24b	-	-	-	-	-	0.85, t (6.5)
25	3.25-3.35, m	3.14, m	3.25-3.35, m	3.46, d (9.9)	3.25-3.35, m	3.25-3.35, m
26	1.32-1.61, m	1.90, m	1.32-1.61, m	1.33-1.65, m	1.32-1.61, m	1.32-1.61, m
26a	0.85, d (6.8)	0.85, d (6.8)	0.85, d (6.8)	0.90, d (7.1)	0.84, d (6.8)	0.84, d (6.8)
27	1.32-1.61, m	1.03, d (6.8)	1.32-1.61, m	1.33-1.65, m	1.32-1.61, m	1.32-1.61, m
28	0.94, t (7.1)	-	0.96, t (7.1)	0.94, t (7.1)	0.94, t (7.1)	0.93, t (7.1)
1′	4.75, brd (2.8)	4.75, brd (2.8)	4.72, brd (2.8)	4.74, brd (2.8)	4.74, brd (2.8)	4.75, brd (2.8)
2′	2.29, m	2.29, m	2.29, m	2.29, m	2.29, m	2.29, m
	1.48, m	1.48, m	1.48, m	1.48, m	1.48, m	1.48, m
3′	3.58, m	3.58, m	overlapped	3.59, m	3.58, m	3.58, m
3′a	3.34, s	3.33, s	-	3.34, s	3.35, s	3.34, s
4′	3.15, m	3.15, m	3.15, m	3.15, m	3.15, t (9.1)	3.15, m
5′	3.80, m	3.79, m	overlapped	3.80, m	3.80, m	3.80, m
6′	1.20, d (9.1)	1.20, d (9.1)	1.20, d (9.1)	1.20, d (9.1)	1.20, d (9.1)	1.20, d (9.1)
1″	5.31, d (3.1)	5.31, d (3.1)	5.37, m	5.31, d (3.1)	5.31, d (3.1)	5.31, d (3.1)
2″	2.23, m	2.23, m	2.23, m	2.23, m	2.23, m	2.23, m
	1.42, m	1.42, m	1.42, m	1.42, m	1.42, m	1.42, m
3″	3.31, m	3.31, m	overlapped	3.31, m	3.31, m	3.31, m
3″a	3.34, s	3.34, s	3.34, s	3.35, s	3.35, s	3.34, s
4″	2.98, t (9.1)	2.98, t (9.1)	3.08, t (9.1)	2.98, dt (3.7, 9.1)	2.98, dt (3.7, 9.4)	2.98, m
5″	3.63, m	3.63, m	3.65, m	3.63, m	3.63, m	3.63, m
6″	1.17, d (6.2)	1.17, d (6.2)	1.17, d (6.2)	1.17, d (6.2)	1.17, d (6.2)	1.16, d (6.2)

5.5. Characterization of fraction A by MS and ^{1}H NMR.

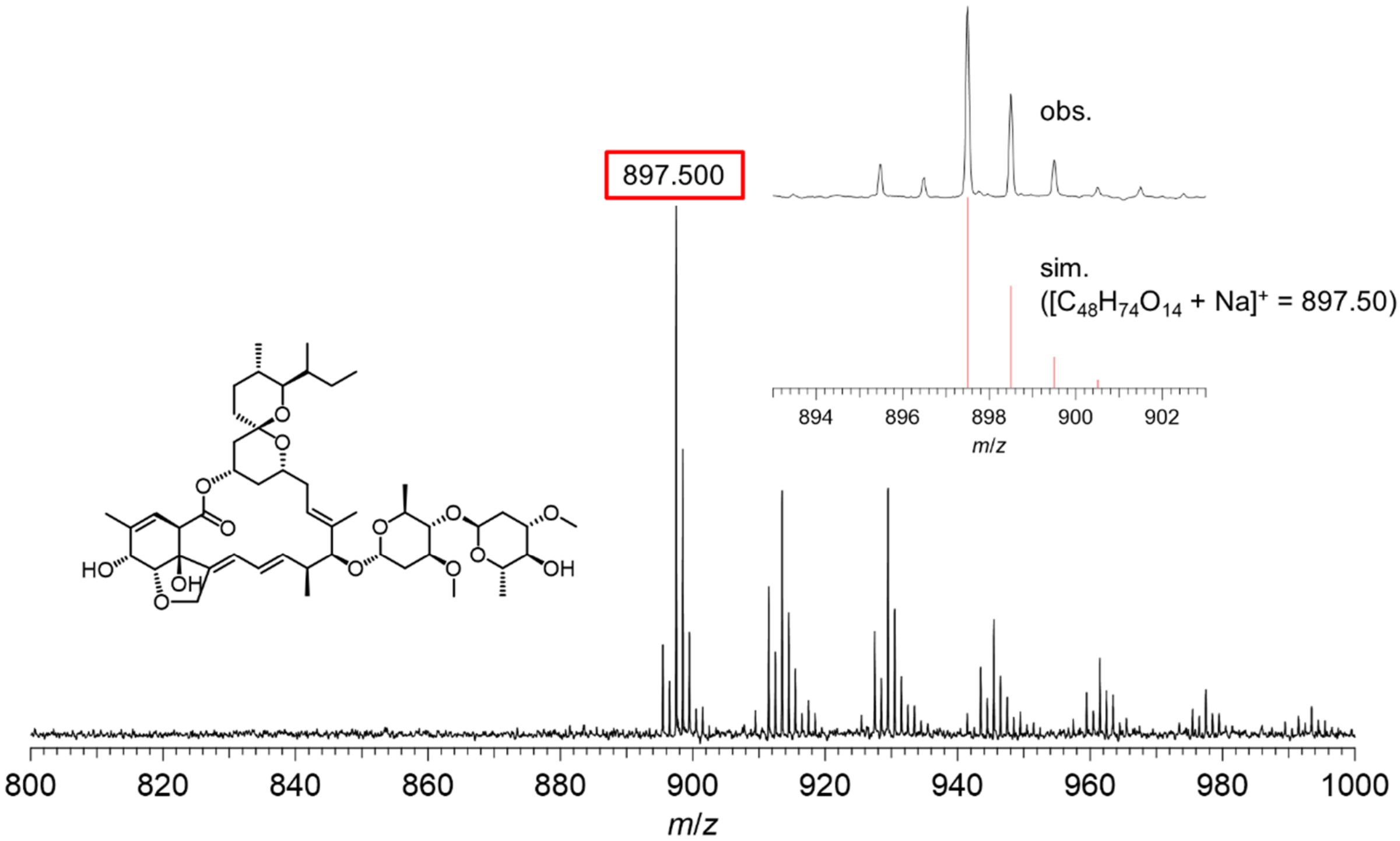

Fig. S30. Mass spectrum of fraction A in methanol. Calcd. for $[C_{48}H_{74}O_{14} + Na]^+$: 897.50, found: 897.50.

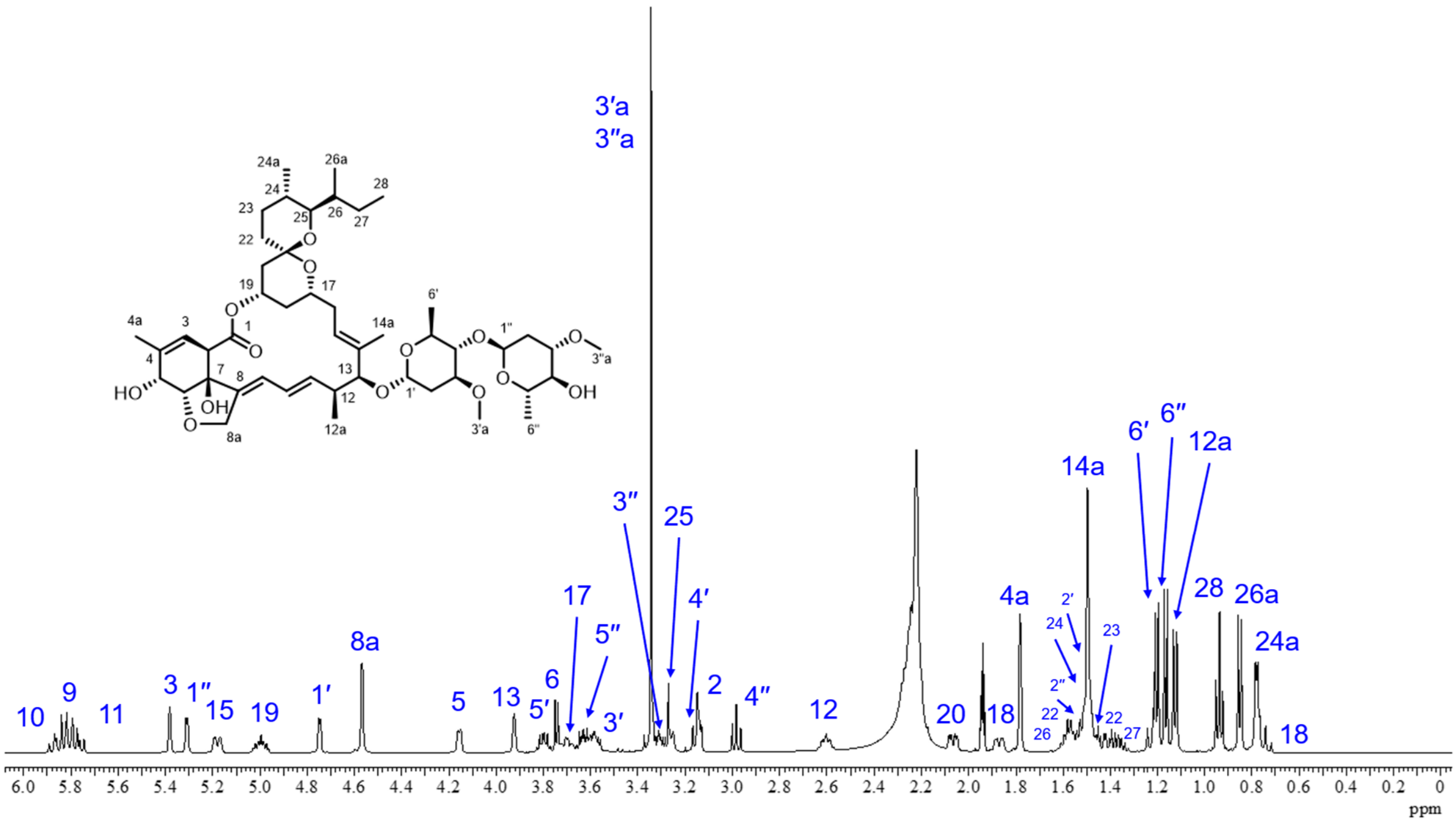

Fig. S31. ^{1}H NMR spectrum of fraction A (acetonitrile-d_3, 500 MHz).

5.6. Structural characterization of fraction B by MS and ^{1}H NMR.

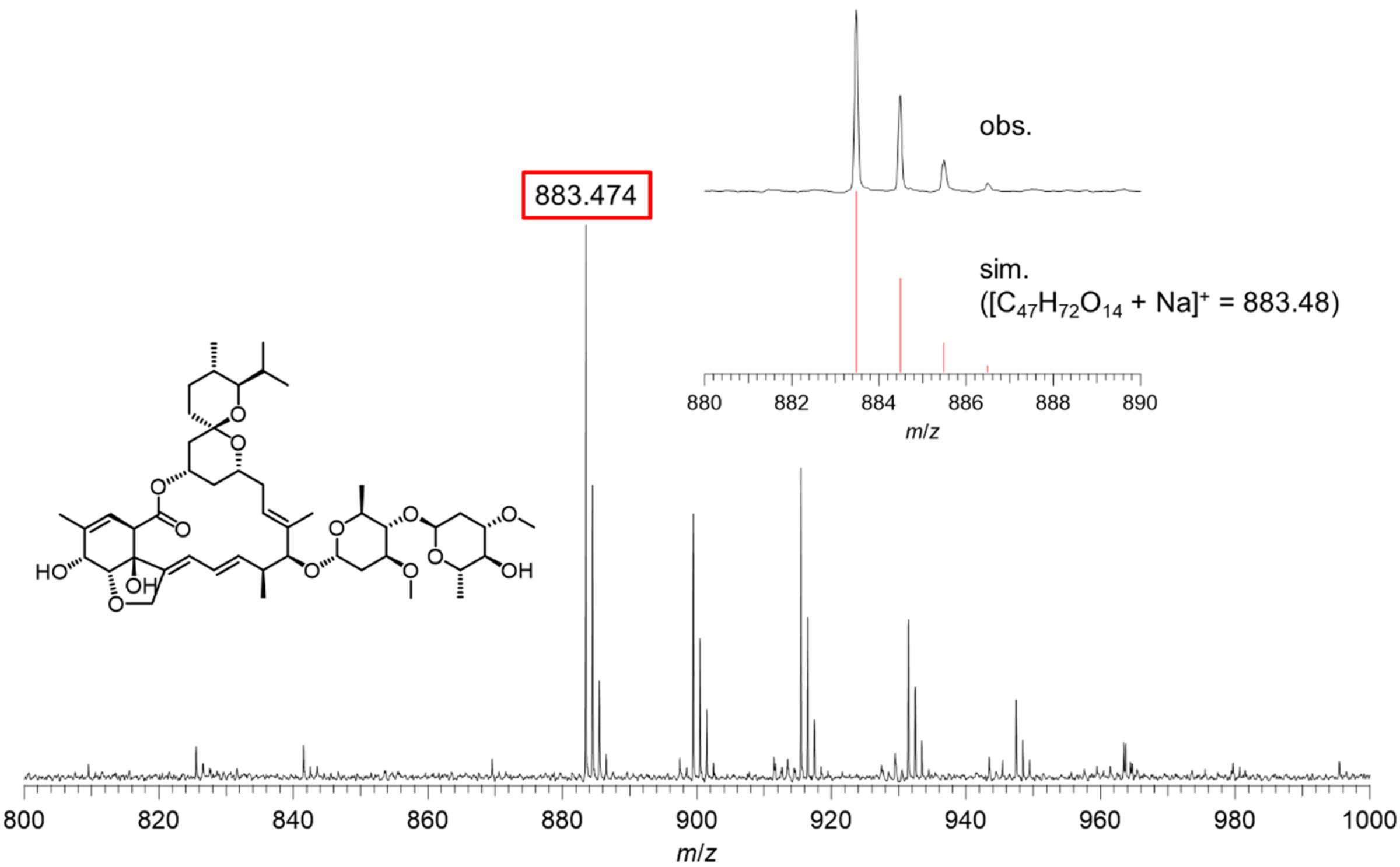

Fig. S32. Mass spectrum of fraction B in methanol. Calcd. for $[C_{47}H_{72}O_{14} + Na]^{+}$: 883.48, found: 883.474.

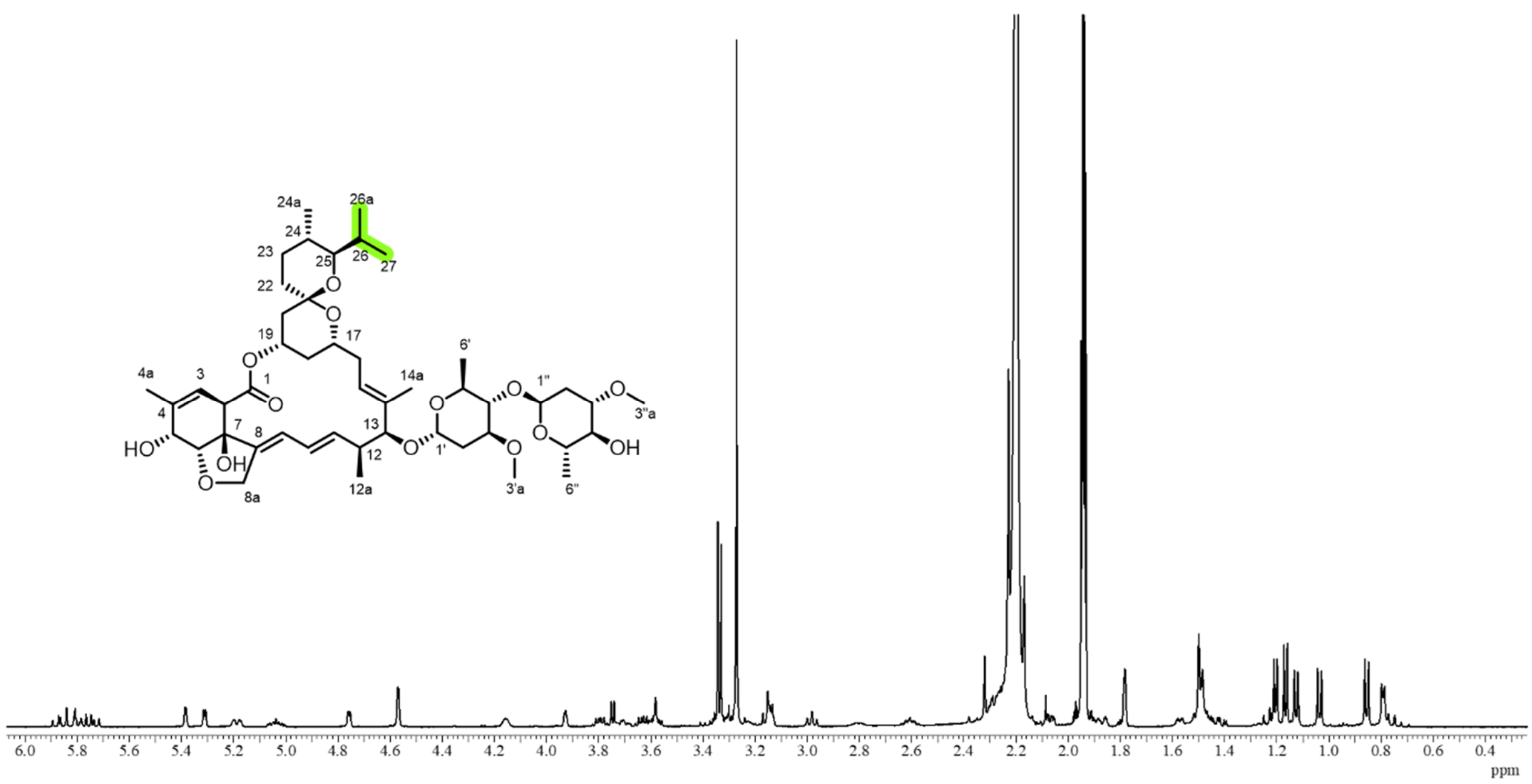

Fig. S33. ^{1}H NMR spectrum of fraction B (acetonitrile-d_3, 500 MHz).

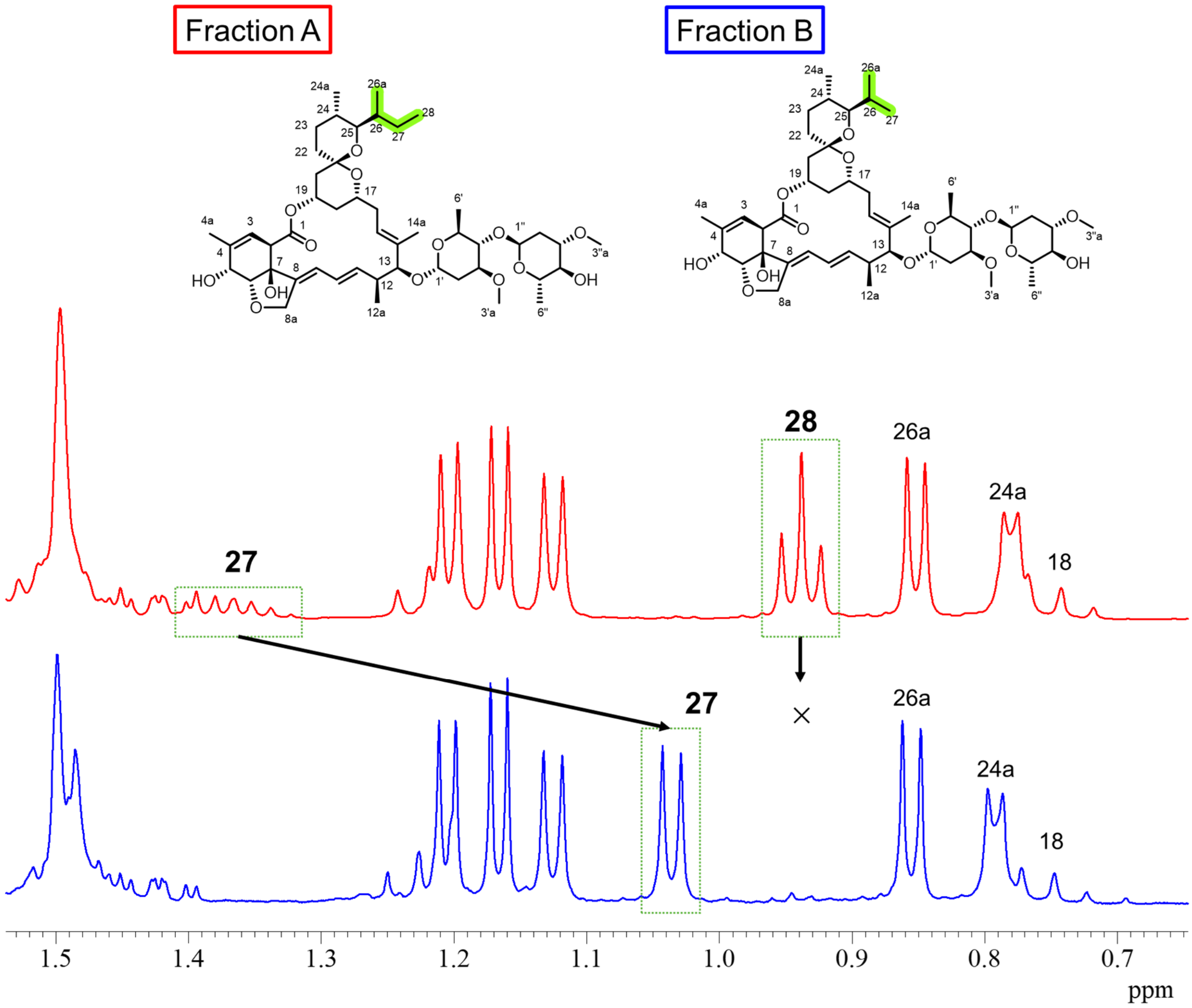

	Position	Multiplicity	δH (*J* in Hz)	Integral value
Fraction A	27	m	1.32–1.61	2H
	28	t	0.94 (7.1)	3H
Fraction B	27	d	1.03 (6.8)	3H
	28	-	-	-

Fig. S34. Comparison of ^{1}H NMR spectra of fractions A (*red*) and B (*blue*).

5.7. Structural characterization of fraction C by MS and ^{1}H NMR.

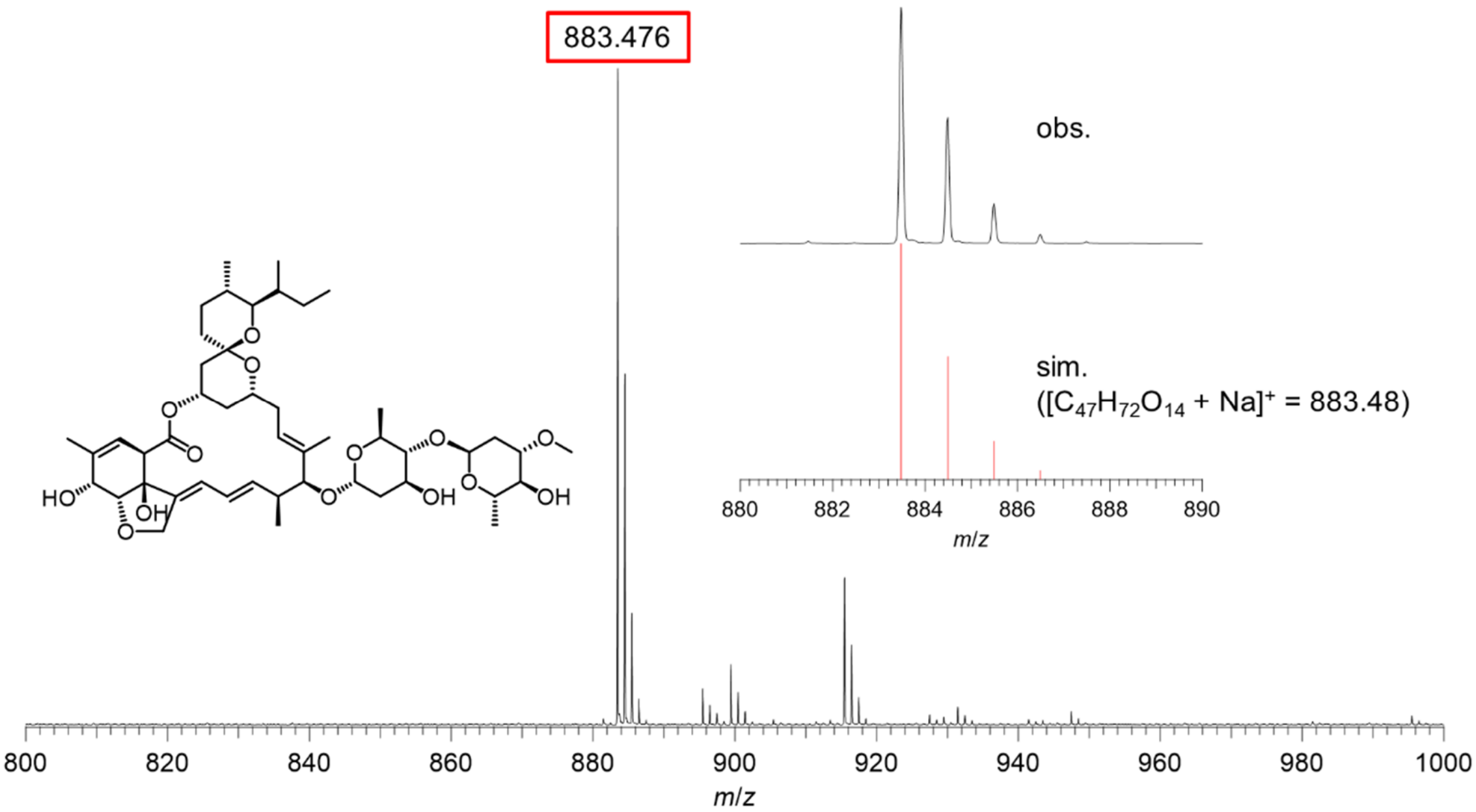

Fig. S35. Mass spectrum of fraction C in methanol. Calcd. for $[C_{47}H_{72}O_{14} + Na]^{+}$: 883.48, found: 883.476.

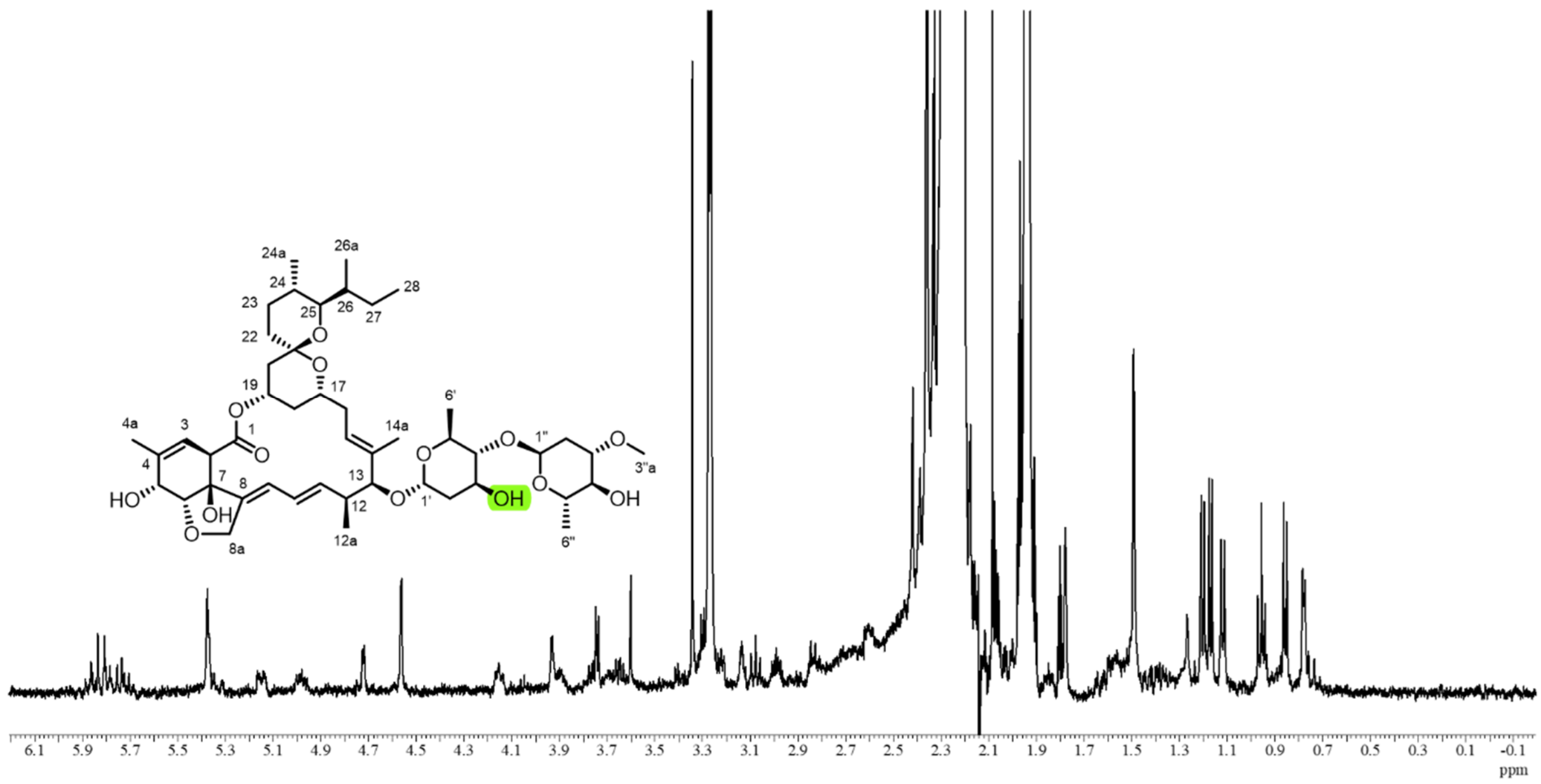

Fig. S36. ^{1}H NMR spectrum of fraction C (acetonitrile-d_3, 500 MHz).

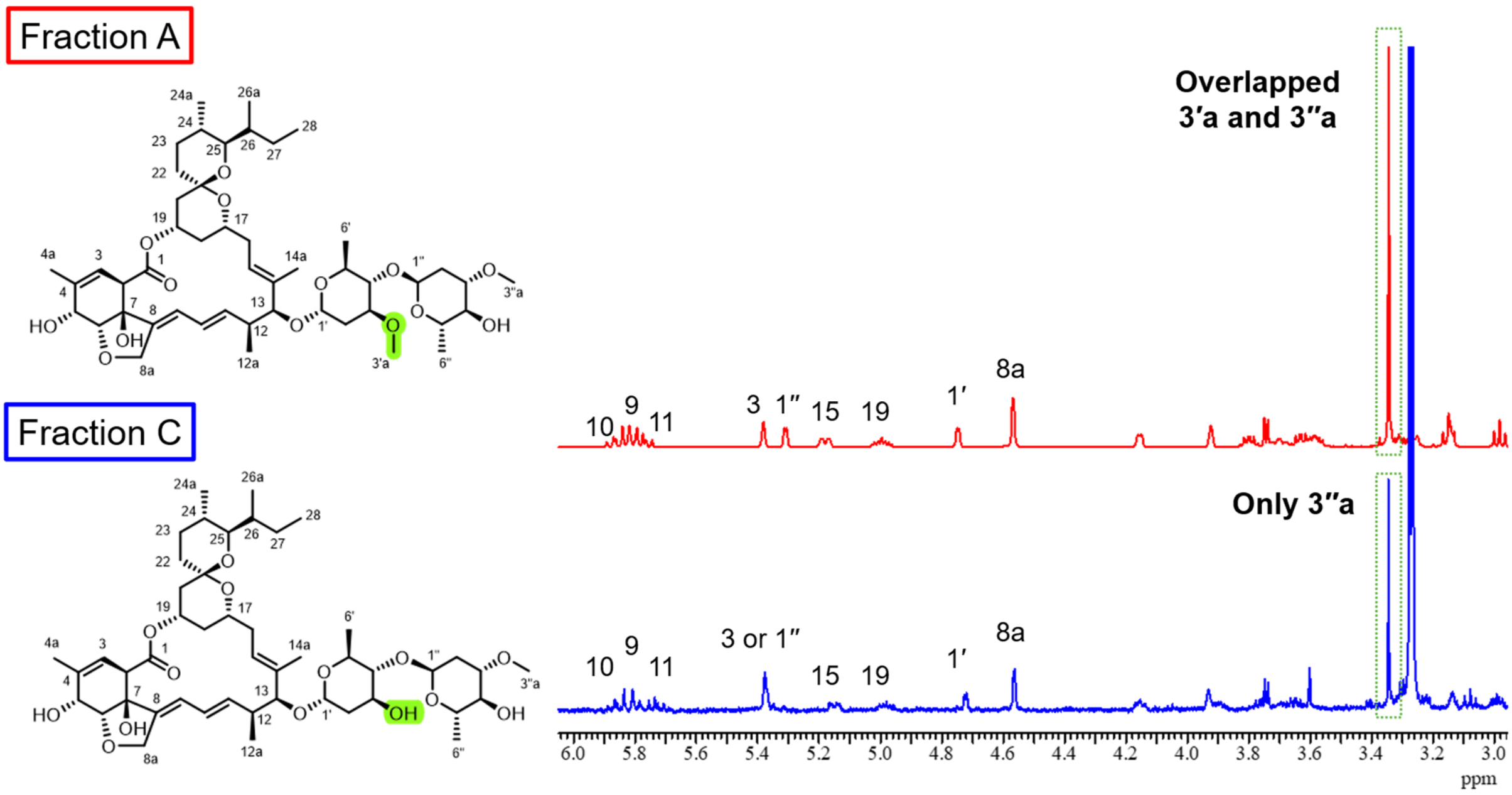

	Position	Multiplicity	δ_H	Integral value
Fraction A	3′a*	s	3.34	3H
	3″a*	s	3.34	3H
Fraction C	3′a	-	-	-
	3″a	s	3.34	3H

Fig. S37. Comparison of ^{1}H NMR spectra of fractions A (*red*) and C (*blue*). The signals denoted by (*) overlapped at 3.35 ppm.

5.8. Structural characterization of fraction D by MS and ^{1}H NMR.

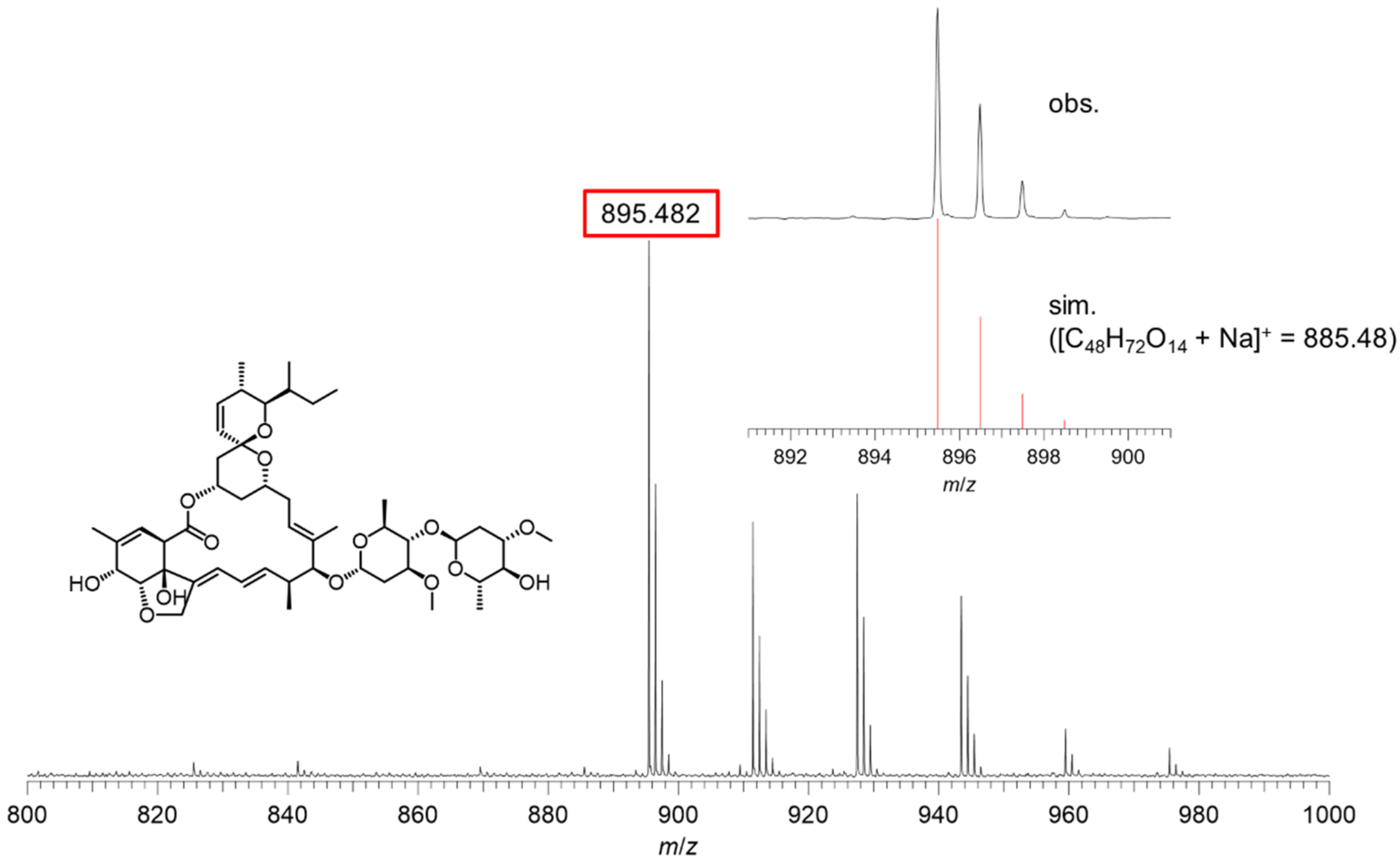

Fig. S38. Mass spectrum of fraction D in methanol. Calcd. for [$C_{48}H_{72}O_{14}$ + Na]$^+$: 895.48, found: 895.482.

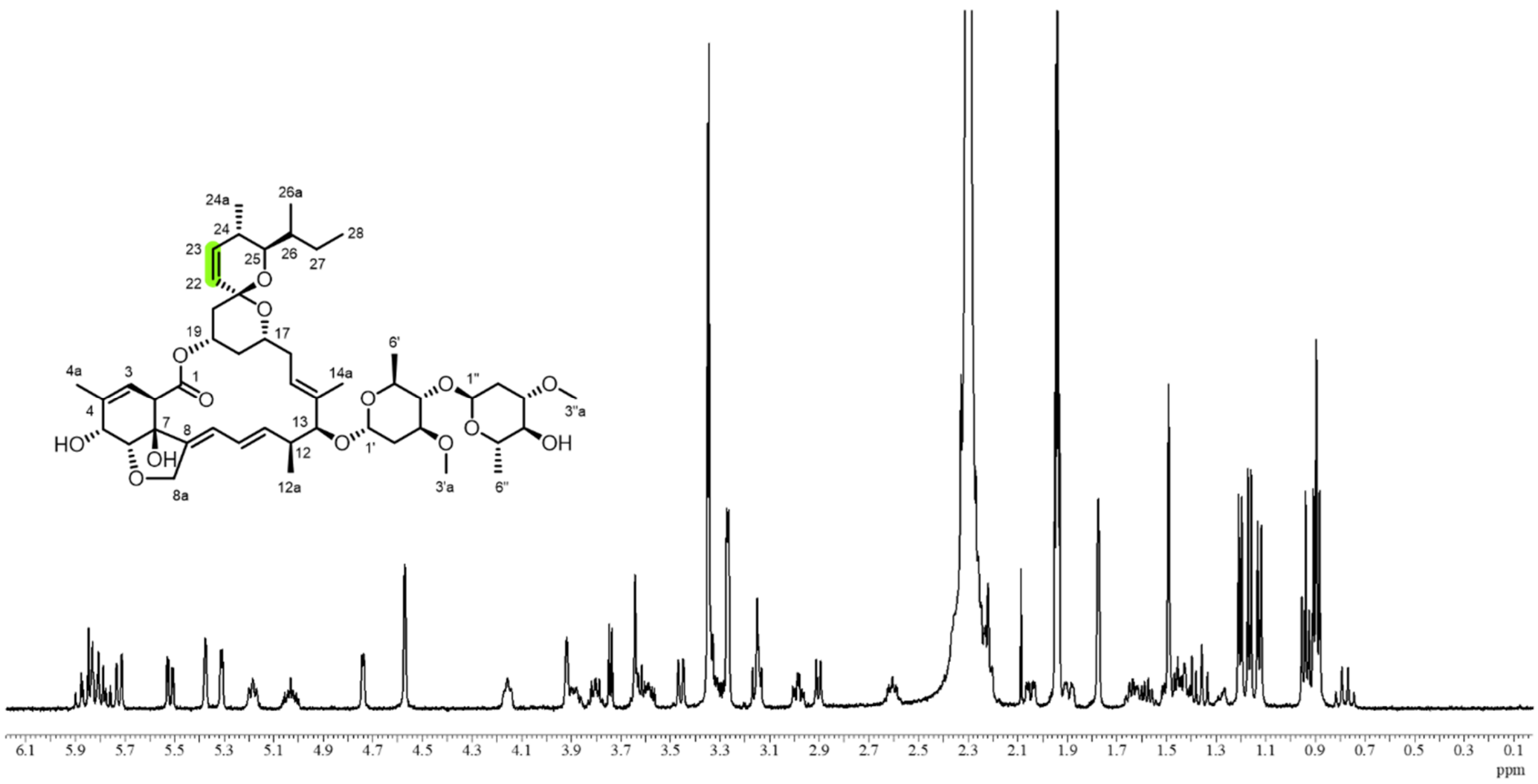

Fig. S39. ^{1}H NMR spectrum of fraction D (acetonitrile-d_3, 500 MHz).

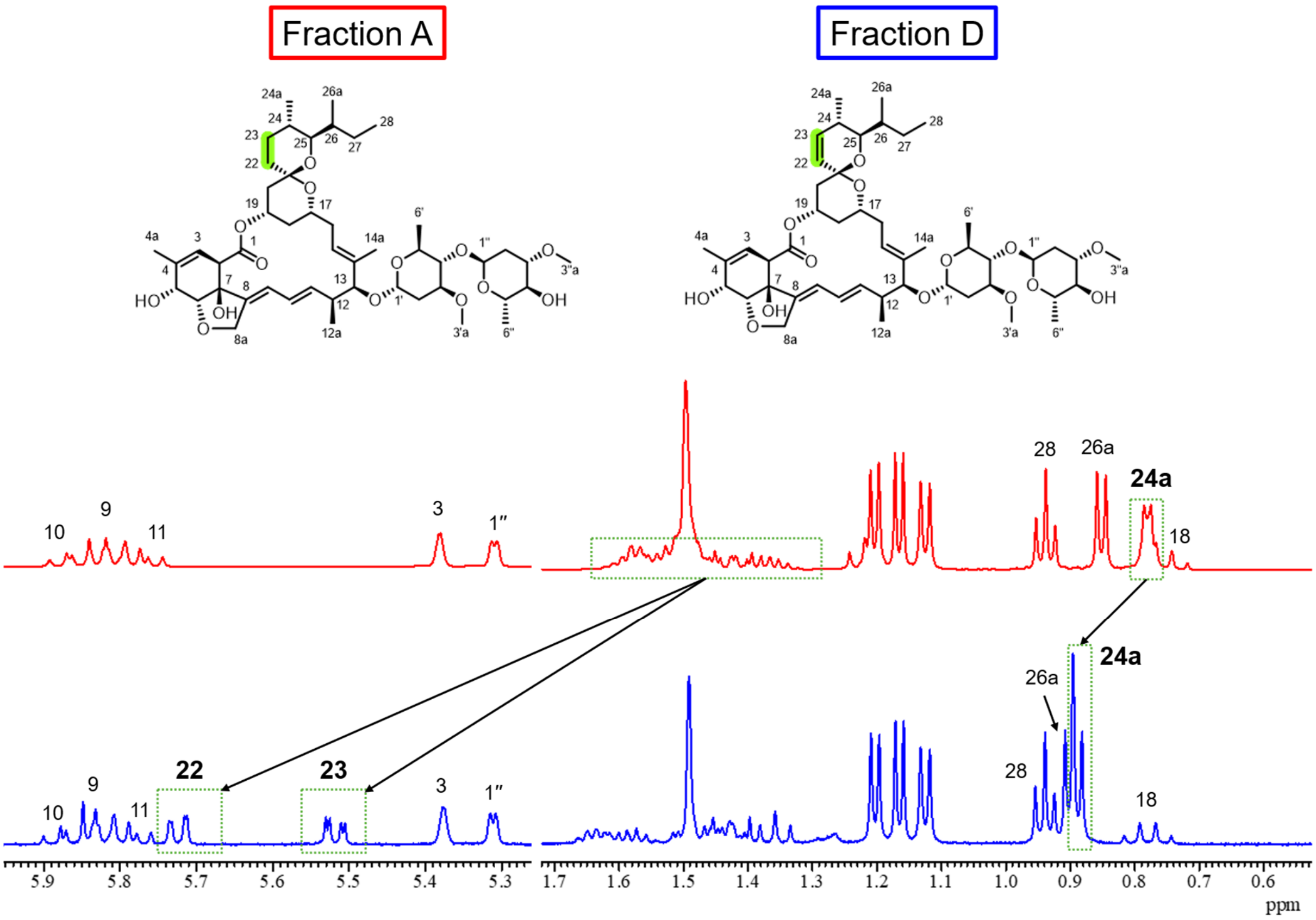

	Position	Multiplicity	δH (*J* in Hz)	Integral value
Fraction A	22	m	1.48, 1.57	2H
	23	m	1.48	2H
	24a	d	0.78 (5.4)	3H
Fraction D	22	dd	5.72 (9.7, 1.7)	1H
	23	dd	5.52 (9.7, 2.6)	1H
	24a	d	0.89 (7.1)	3H

Fig. S40. Comparison of ^{1}H NMR spectra of fractions A (*red*) and D (*blue*).

5.9. Structural characterization of fraction E by MS and ^{1}H NMR.

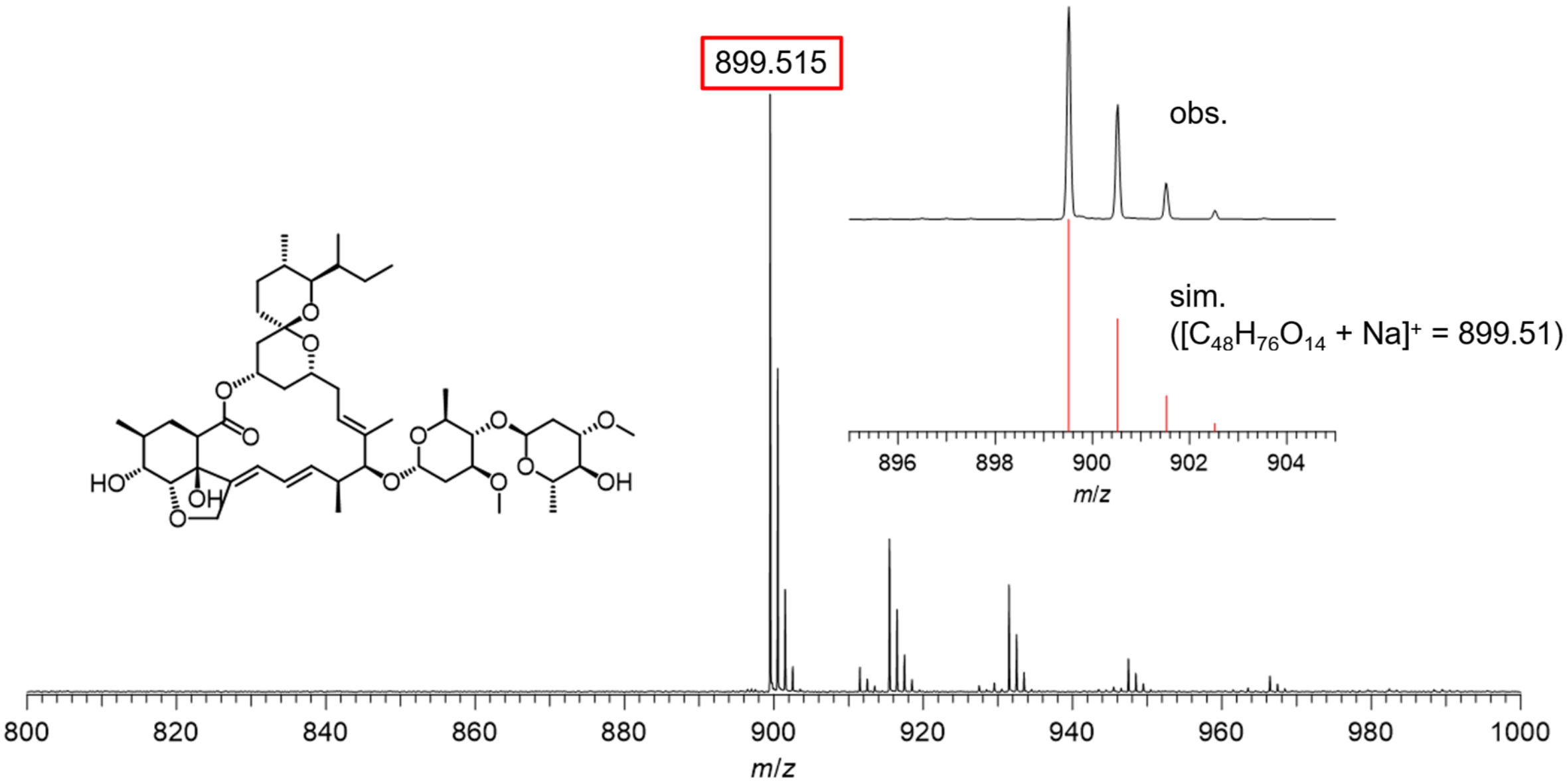

Fig. S41. Mass spectrum of fraction E in methanol. Calcd. for [$C_{48}H_{76}O_{14}$ + Na]$^+$: 899.51, found: 899.515.

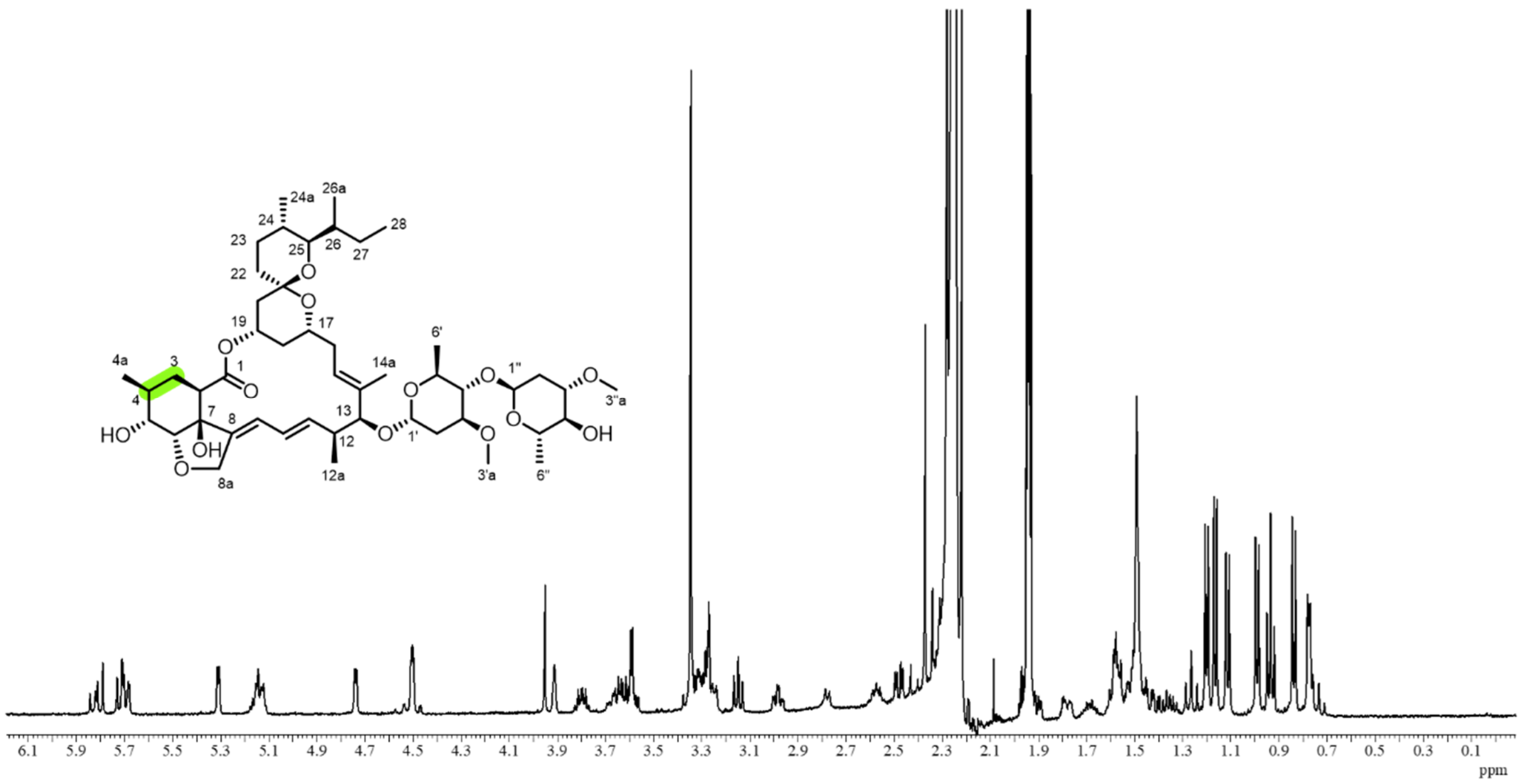

Fig. S42. ^{1}H NMR spectrum of fraction E (acetonitrile-d_3, 500 MHz).

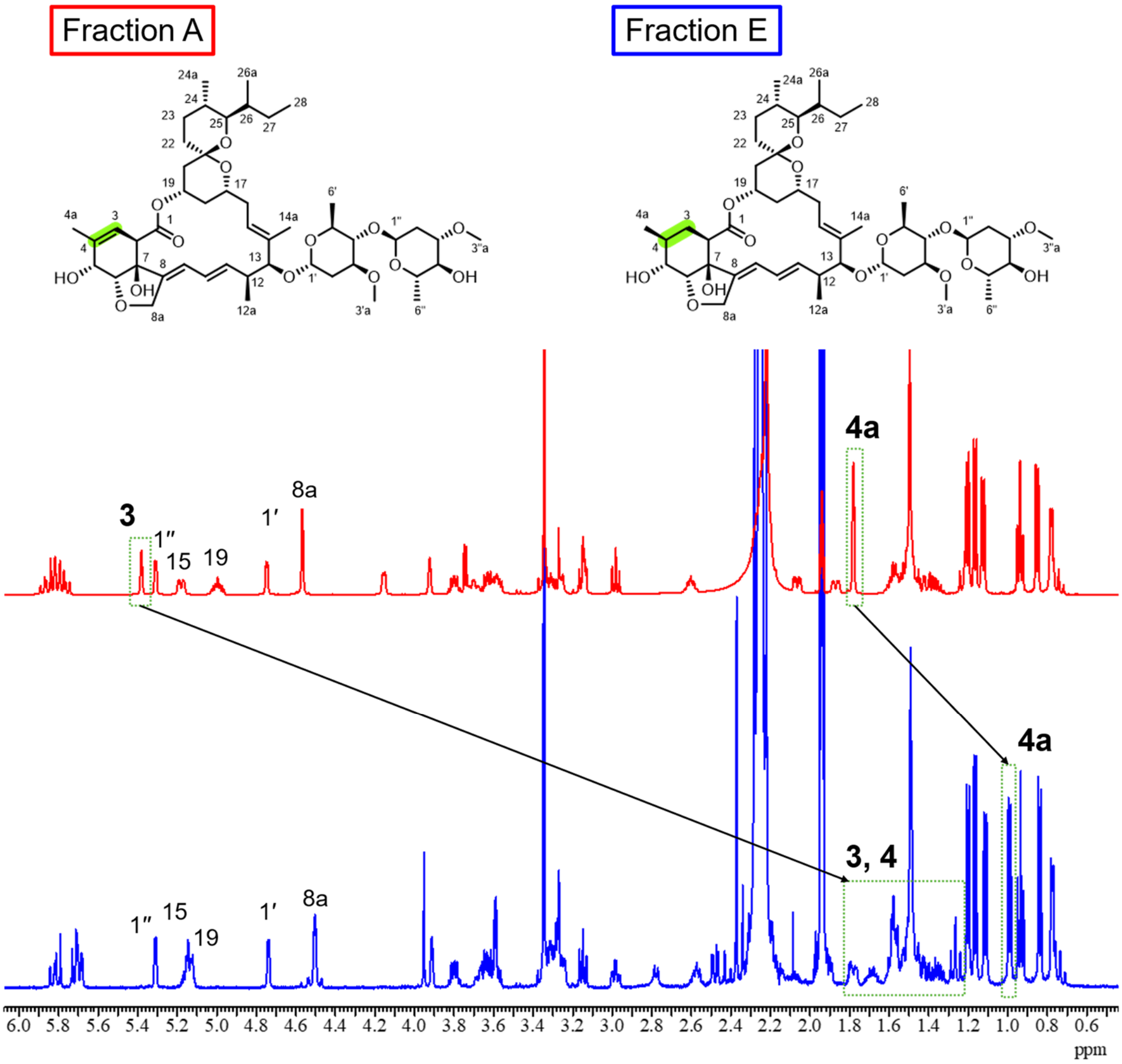

	Position	Multiplicity	δ_H (J in Hz)	Integral value
Fraction A	3	s	5.38	1H
	4	-	-	-
	4a	brs	1.78	3H
Fraction E	3	m	1.24–1.82	2H
	4	m	1.24–1.82	1H
	4a	d	0.99 (6.5)	3H

Fig. S43. Comparison of ^{1}H NMR spectra of fractions A (*red*) and E (*blue*).

5.10. Structural characterization of fraction F by MS and ^{1}H NMR.

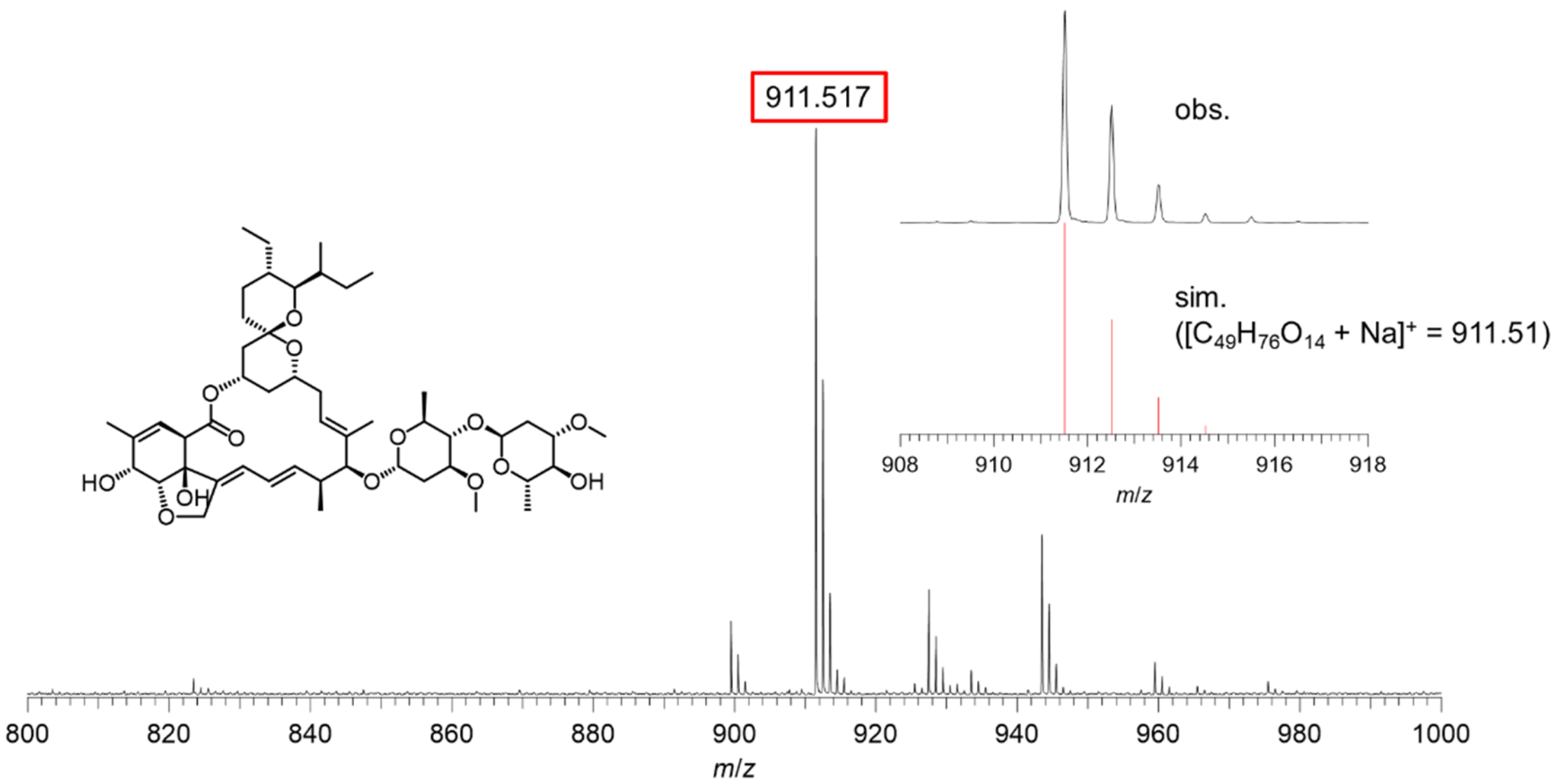

Fig. S44. Mass spectrum of fraction F in methanol. Calcd. for [$C_{49}H_{76}O_{14}$ + Na]$^{+}$: 911.51, found: 911.517.

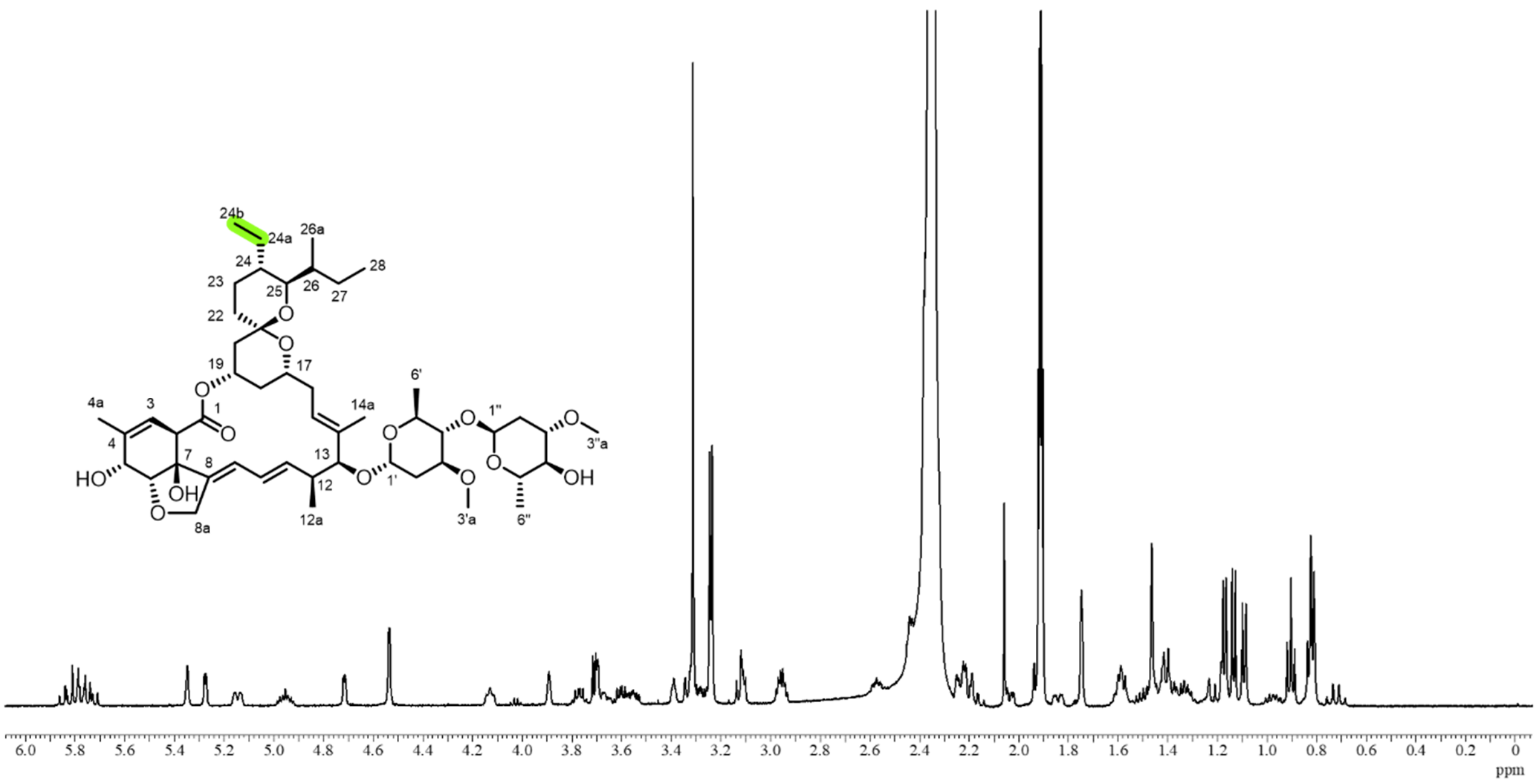

Fig. S45. ^{1}H NMR spectrum of fraction F (acetonitrile-d_3, 500 MHz).

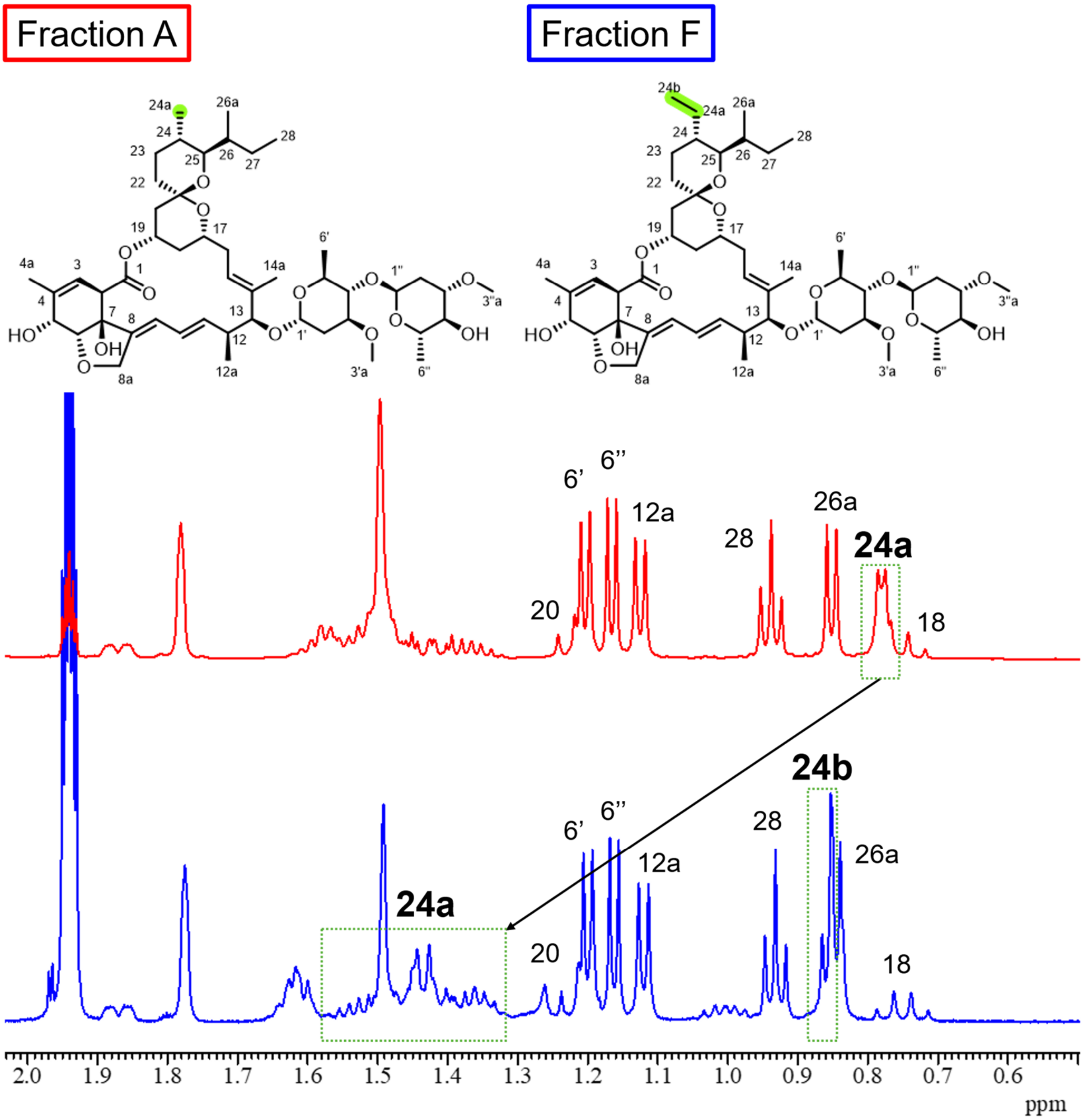

	Position	Multiplicity	δH (*J* in Hz)	Integral value
Fraction A	24a	d	0.78 (5.4)	3H
	24b	-	-	-
Fraction F	24a	m	1.33–1.55	2H
	24b	t	0.85 (6.5)	3H

Fig. S46. Comparison of ^{1}H NMR spectra of fractions A (*red*) and F (*blue*).

5.11. ^{1}H NMR spectra of mixed fraction 1, 2, 3 and 4.

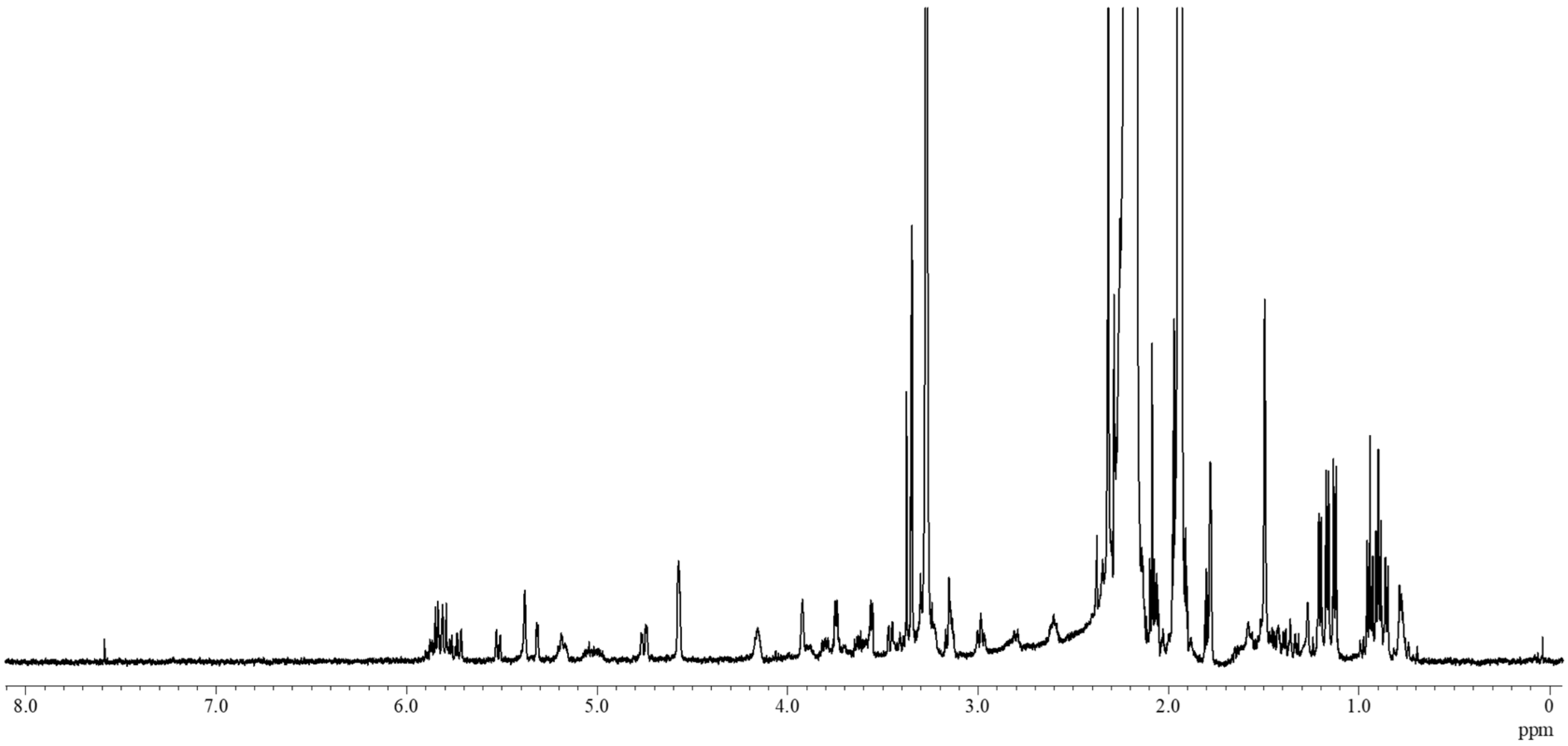

Fig. S47. ^{1}H NMR spectrum of mixed fraction 1 (acetonitrile-d_3, 500 MHz).

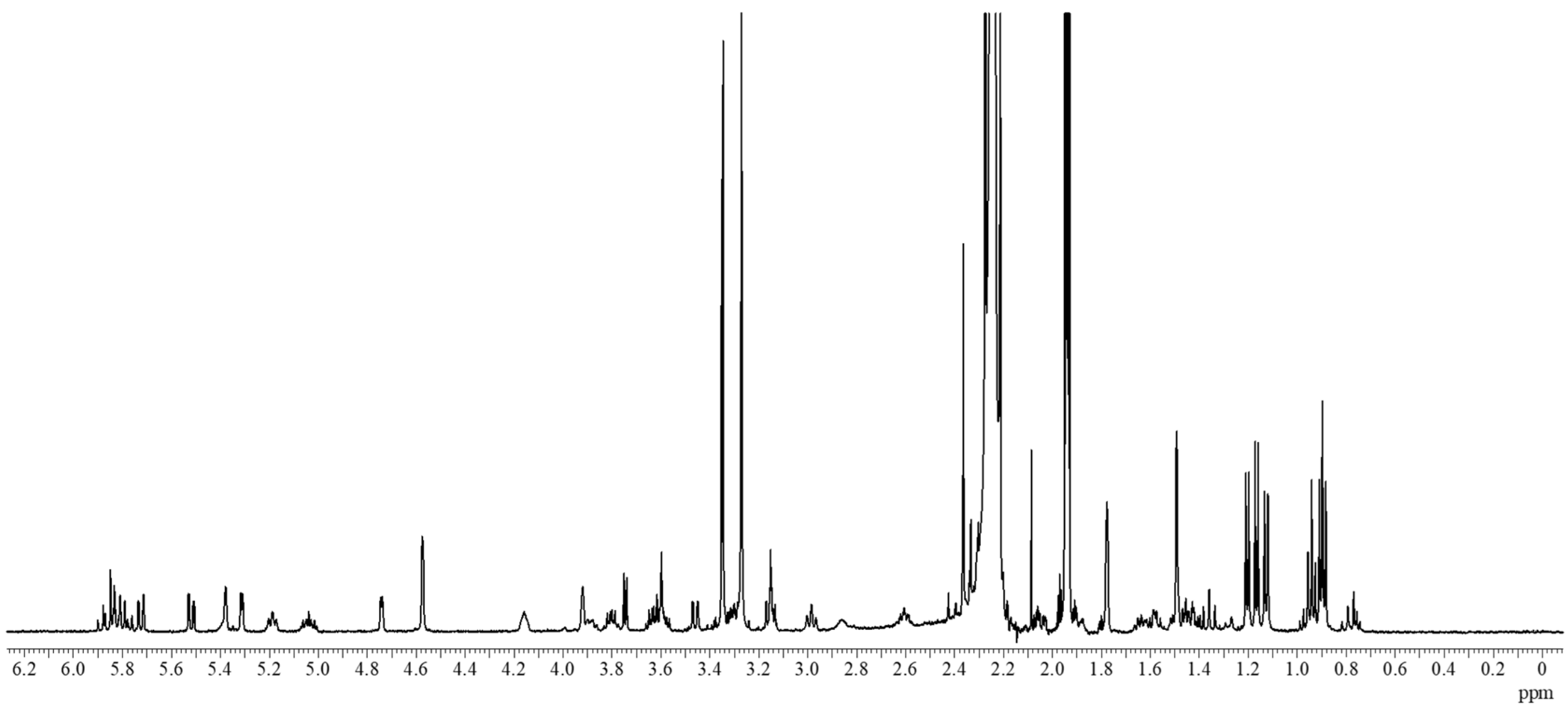

Fig. S48. ^{1}H NMR spectrum of mixed fraction 2 (acetonitrile-d_3, 500 MHz).

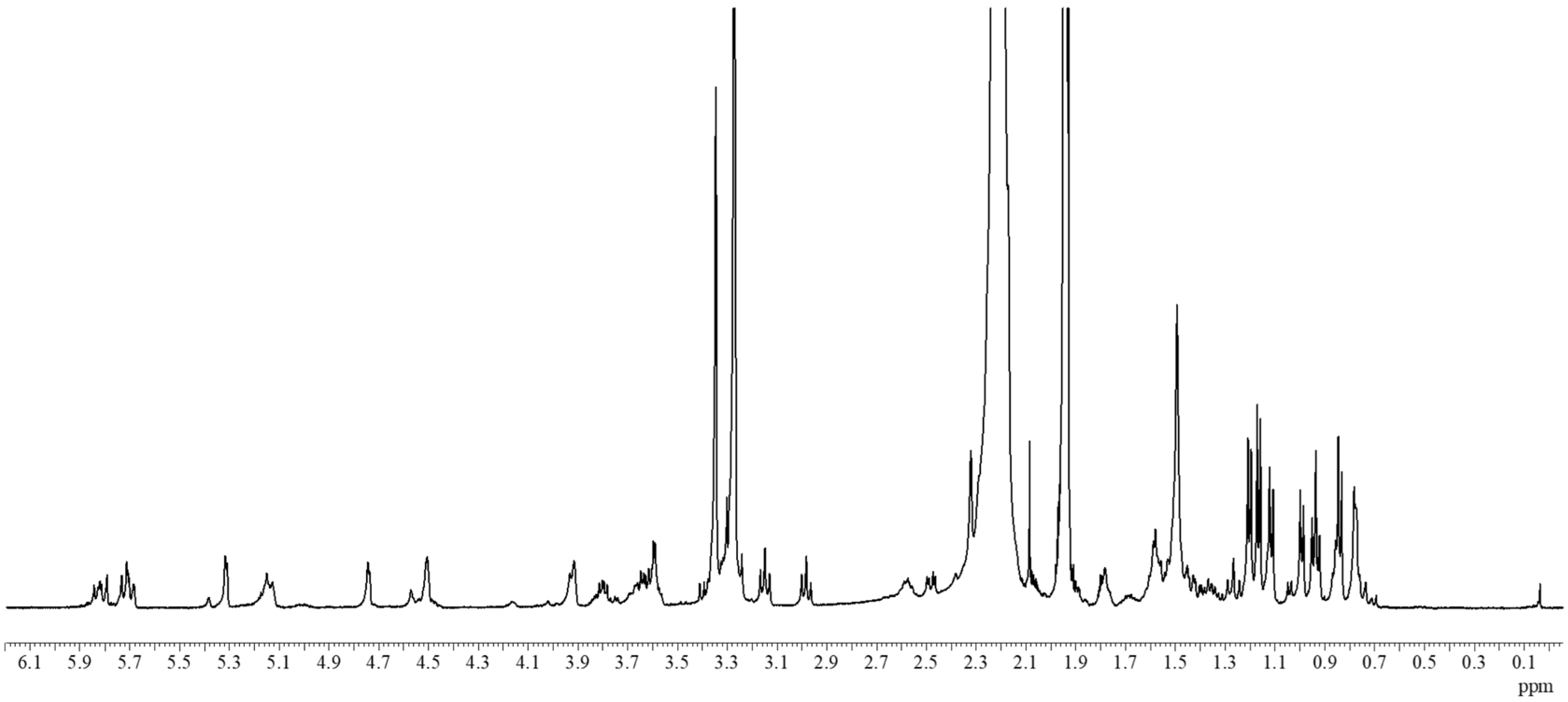

Fig. S49. ^{1}H NMR spectrum of mixed fraction 3 (acetonitrile-d_3, 500 MHz).

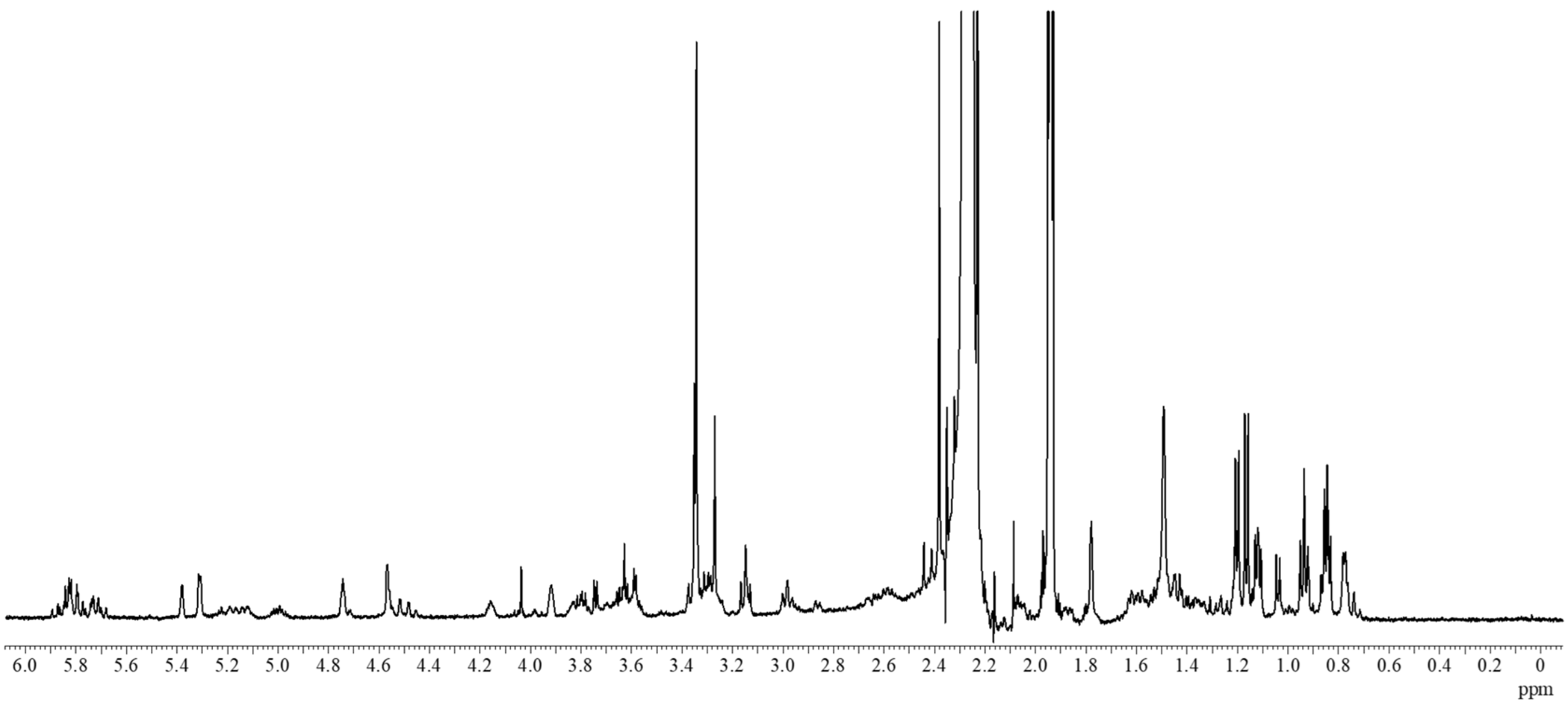

Fig. S50. ^{1}H NMR spectrum of mixed fraction 4 (acetonitrile-d_3, 500 MHz).

6. Structural determination of HPLC-separated ivermectin derivatives by encapsulation within APF-40.

6.1. Single crystal X-ray analysis of ivermectin derivatives.

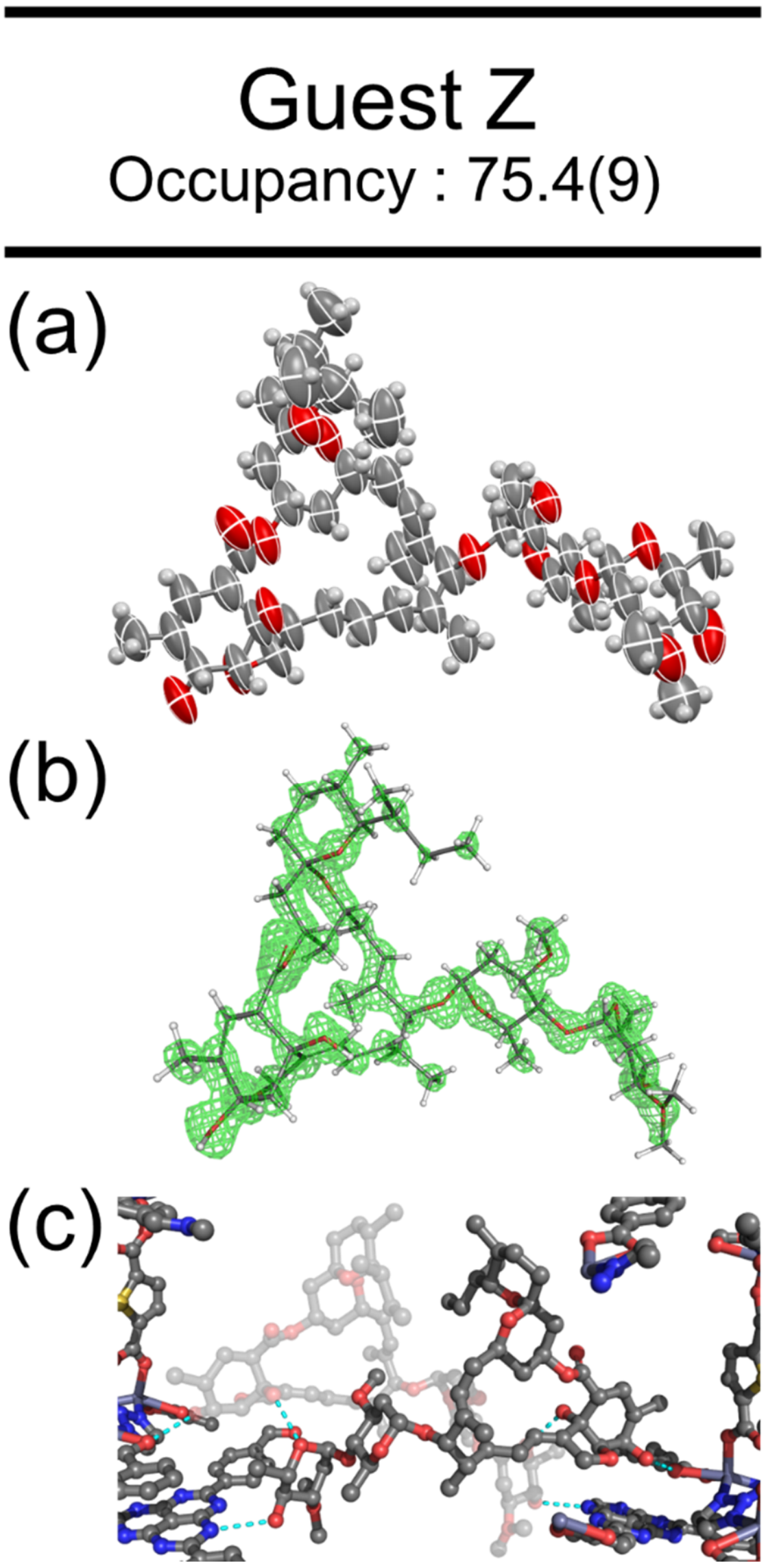

Fig. S51. Encapsulated structure summary of ivermectin B_{1a} purified by HPLC. a) Thermal ellipsoid model of ivermectin B_{1a} with 50% probability, b) 2Fo–Fc electron density map (map threshold: 0.75 e/Å^3), c) interactions between HTCHAP^{3-} and ivermectin B_{1a}. Cyan dash lines represent hydrogen bond contacts. Atom coloring scheme: Zn – purple, C – grey, H – white, O – red, N – blue and S – yellow.

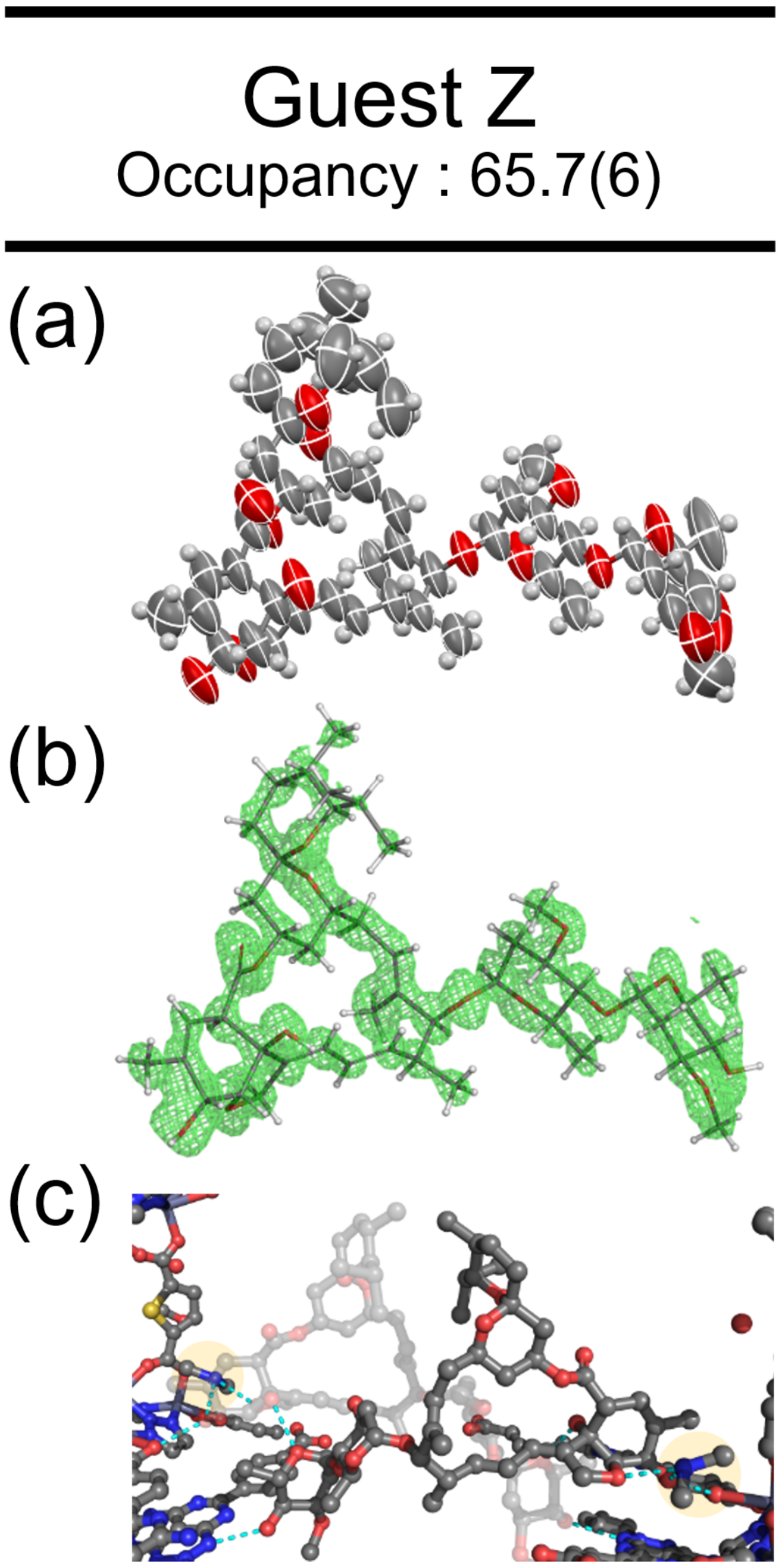

Fig. S52. Ivermectin B_{1b} encapsulated structure summary. a) Thermal ellipsoid model of ivermectin B_{1b} with 50% probability, b) 2Fo–Fc electron density map (map threshold: 0.95 e/Å^3), c) interactions between $HTCHAP^{3-}$, $NH_2Me_2^+$ ions and ivermectin B_{1b}. Cyan dash lines represent hydrogen bond contacts. Atom coloring scheme: Zn – purple, C – grey, H – white, O – red, N – blue and S – yellow.

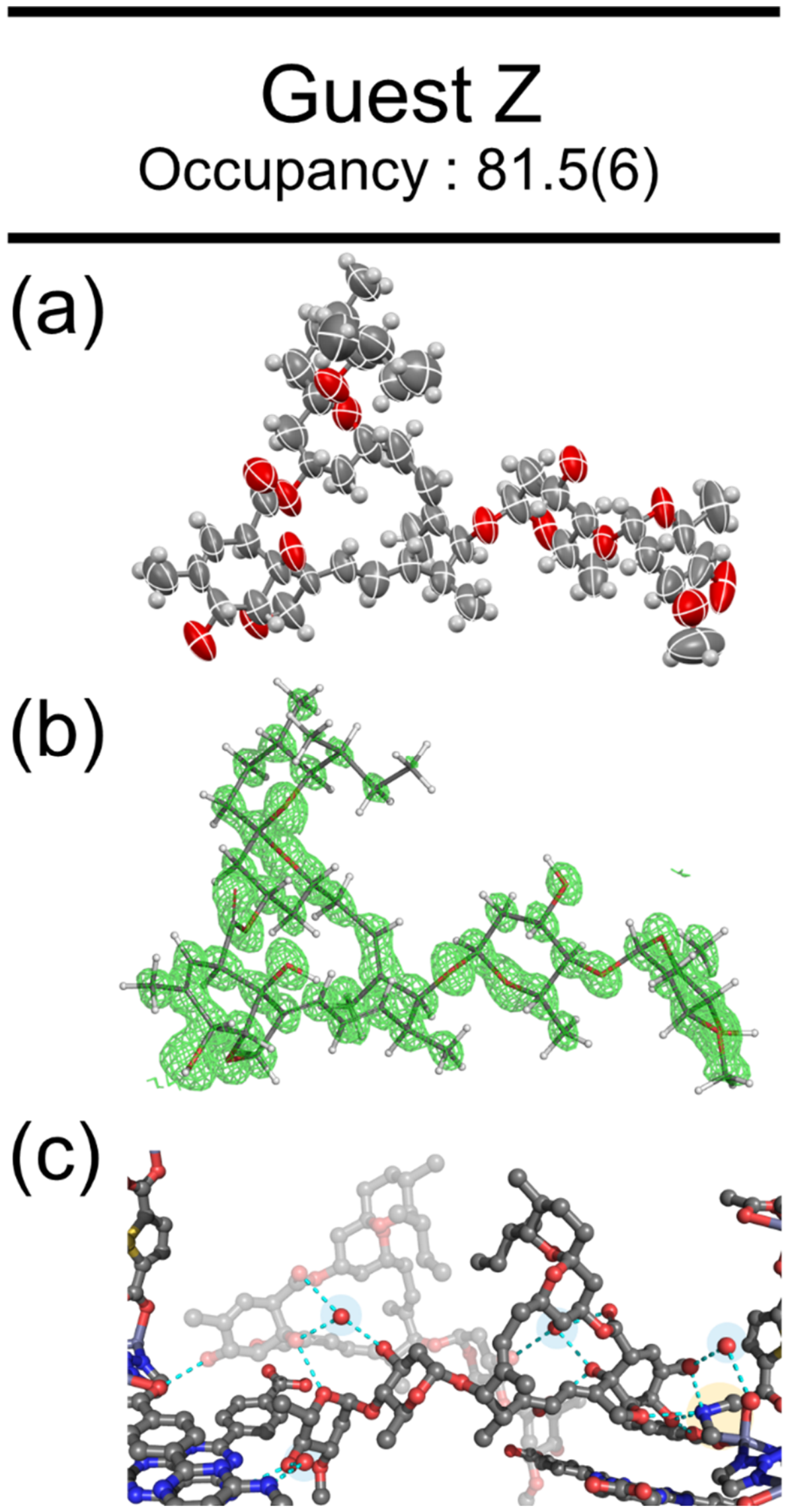

Fig. S53. 3′-*O*-Demethylivermectin B_{1a} encapsulated structure summary. a) Thermal ellipsoid model of 3′-*O*-demethylivermectin B_{1a} with 50% probability, b) 2Fo–Fc electron density map (map threshold: 1.02 e/Å^3), c) interactions between $HTCHAP^{3-}$, water, $NH_2Me_2^+$ ions and 3′-*O*-demethylivermectin B_{1a}. Cyan dash lines represent hydrogen bond contacts. Atom coloring scheme: Zn – purple, C – grey, H – white, O – red, N – blue and S – yellow.

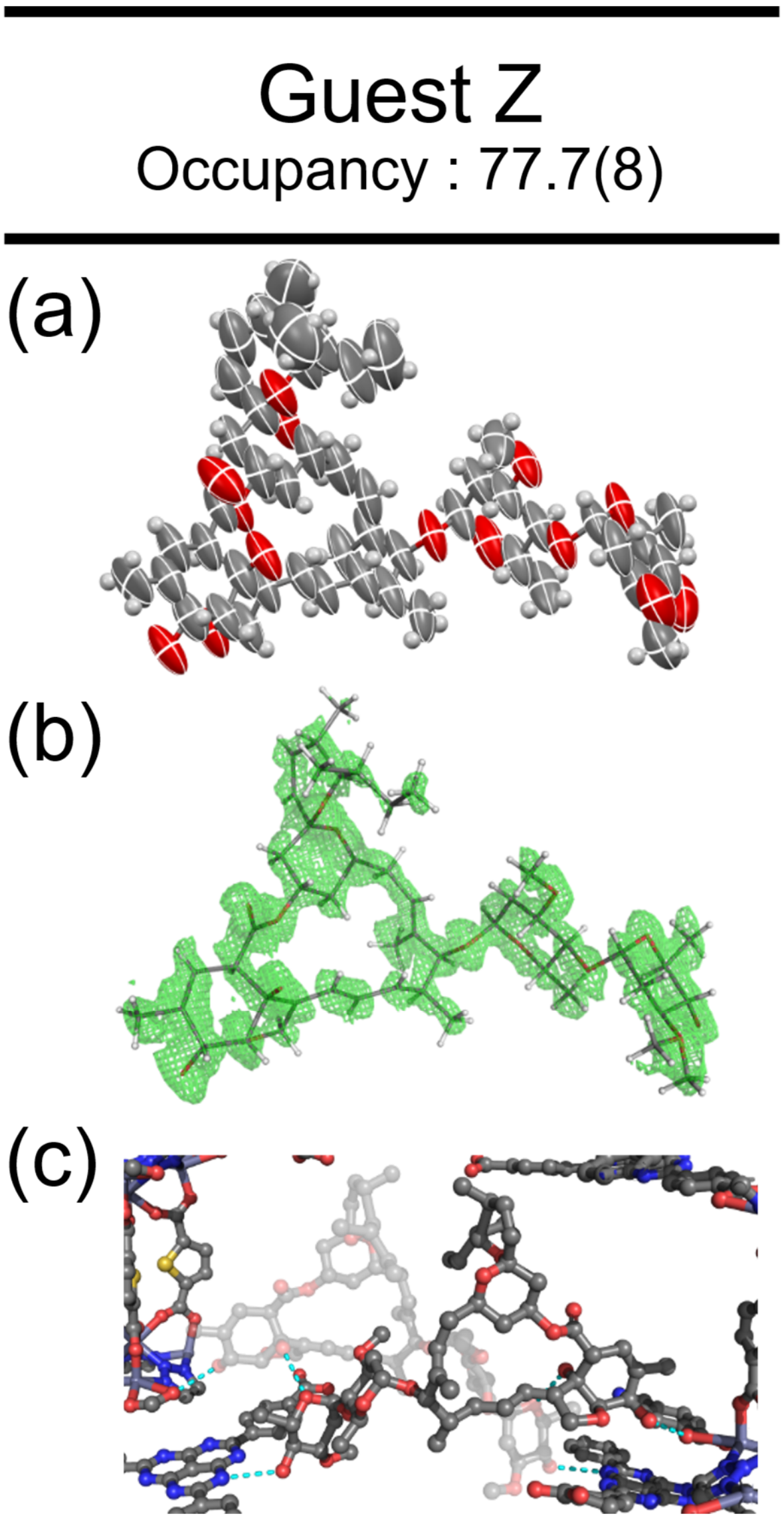

Fig. S54. Avermectin B_{1a} encapsulated structure summary. a) Thermal ellipsoid model of avermectin B_{1a} with 50% probability, b) 2Fo–Fc electron density map (map threshold: 1.02 e/Å^3), c) interactions between HTCHAP^{3-} and avermectin B_{1a}. Cyan dash lines represent hydrogen bond contacts. Atom coloring scheme: Zn – purple, C – grey, H – white, O – red, N – blue and S – yellow.

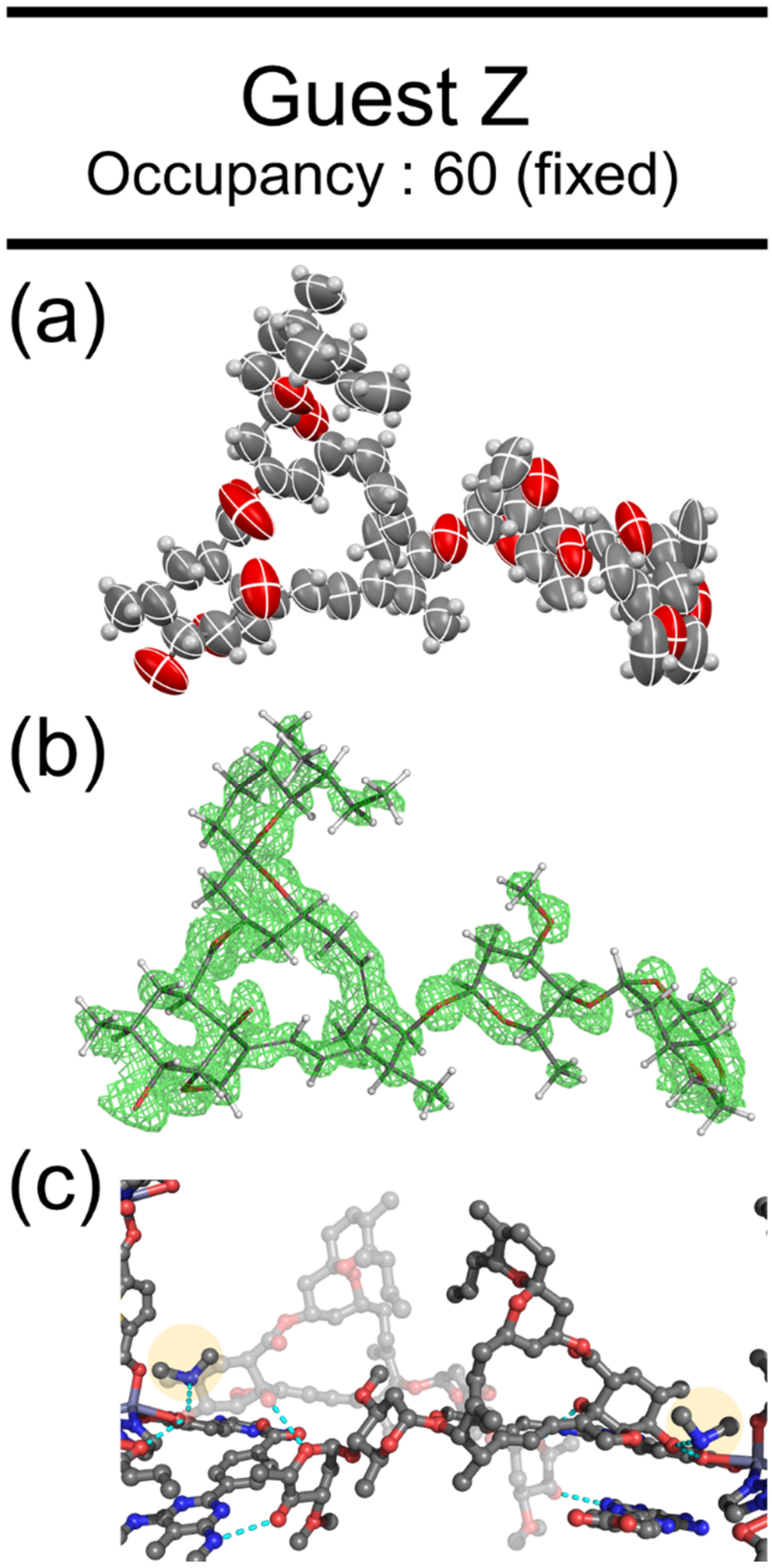

Fig. S55. 3,4-Dihydroivermectin B_{1a} encapsulated structure summary. a) Thermal ellipsoid model of 3,4-dihydroivermectin B_{1a} with 50% probability, b) 2Fo–Fc electron density map (map threshold: 0.90 e/Å^3), c) interactions between $HTCHAP^{3-}$, $NH_2Me_2^+$ ions and 3,4-dihydroivermectin B_{1a}. Cyan dash lines represent hydrogen bond contacts. Atom coloring scheme: Zn – purple, C – grey, H – white, O – red, N – blue and S – yellow.

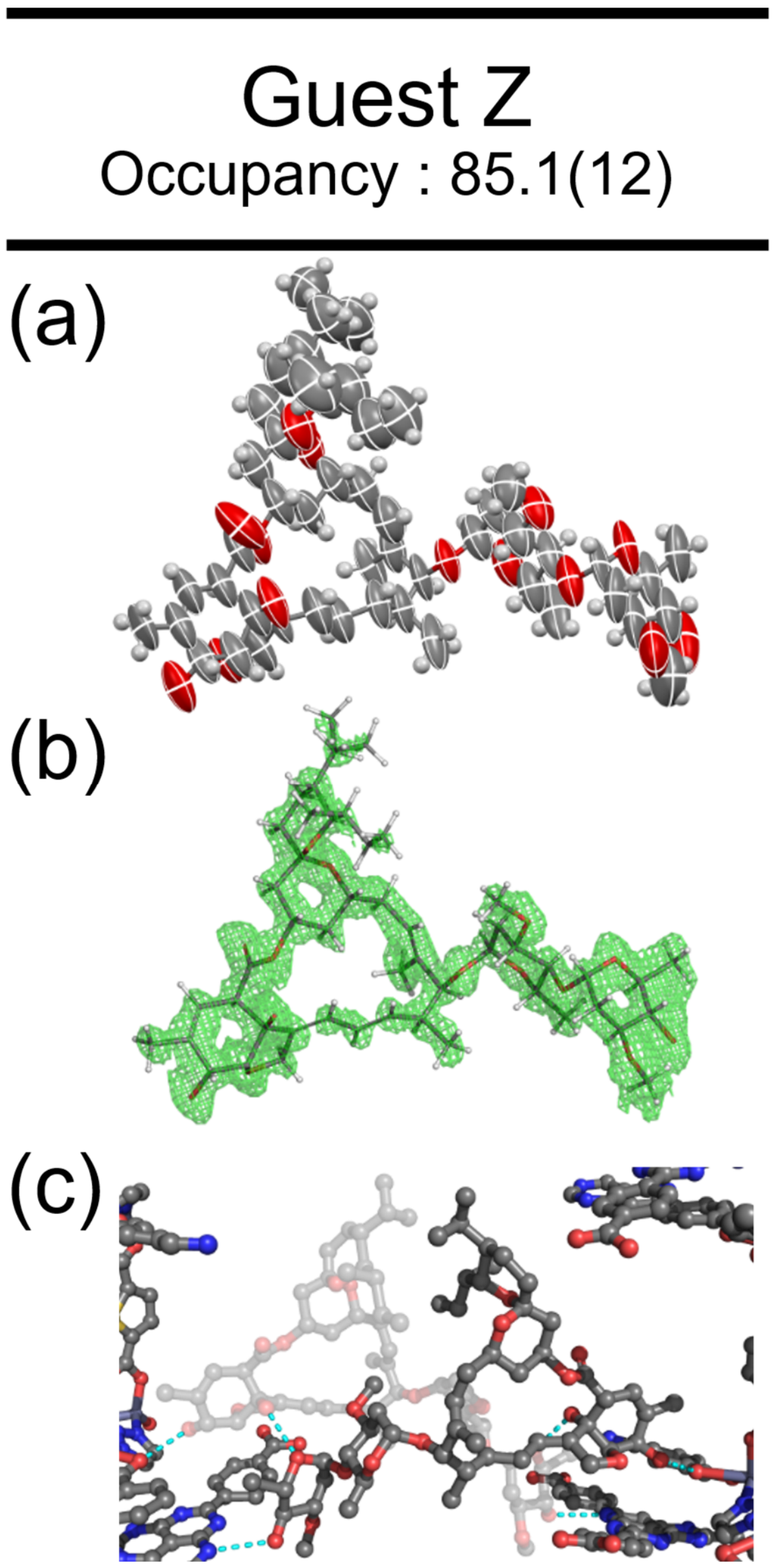

Fig. S56. Ethyl-ivermectin encapsulated structure summary. a) Thermal ellipsoid model of ethyl-ivermectin with 50% probability, b) 2Fo–Fc electron density map (map threshold: 0.75 e/Å^3), c) interactions between HTCHAP^{3-} and ethyl-ivermectin. Cyan dash lines represent hydrogen bond contacts. Atom coloring scheme: Zn – purple, C – grey, H – white, O – red, N – blue and S – yellow.

6.2. Proposed biosynthetic mechanism for the introduction of ethyl group into ethyl-ivermectin.

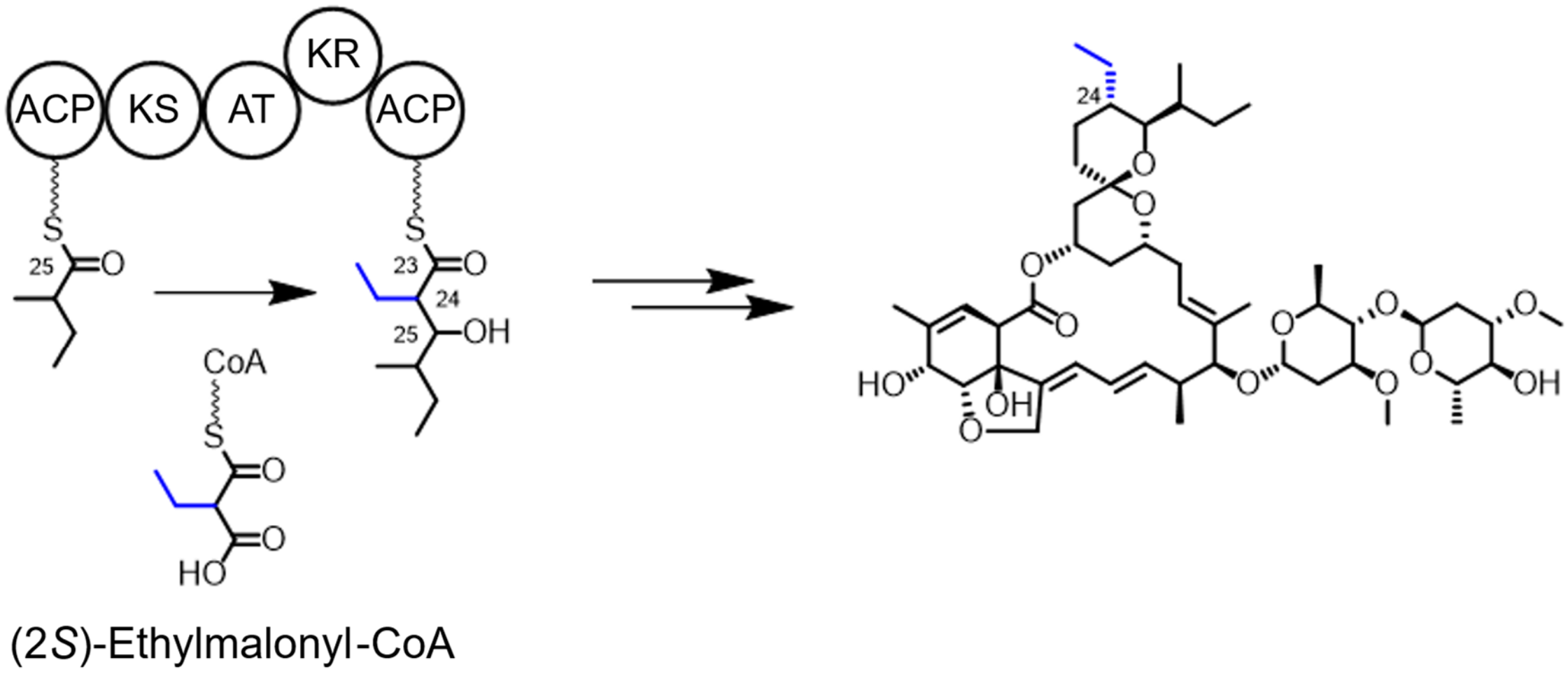

Fig. S57. Proposed biosynthetic mechanism for the introduction of ethyl group on C24. ACP – acyl carrier protein, KS – β-ketosynthase, AT – acyltransferase and KR – β-ketoreductase[23].

6.3. Comparison of isotropic displacement parameters of ivermectin B_{1a} before and after HPLC separation.

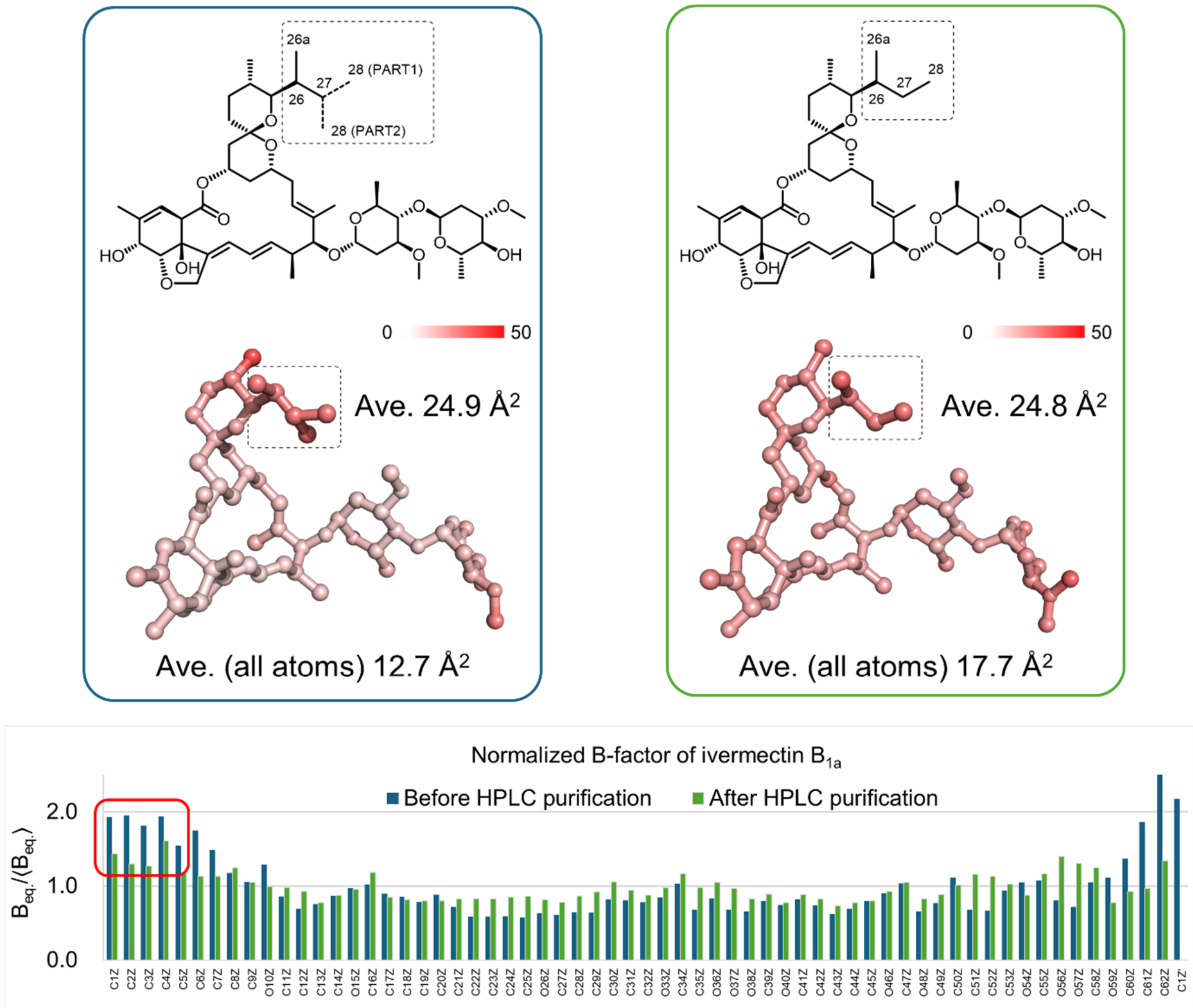

Fig. S58. Comparison of isotropic displacement parameters ($B_{eq.}$) of ivermectin B_{1a} before (*blue*) and after (*light green*) HPLC separation. Magnitudes of $B_{eq.}$ of each atom in molecular structures represented by red color maps (*top*). Normalized B-factors ($B_{eq.}/\langle B_{eq.}\rangle$) of each atom in ivermectin B_{1a} (*bottom*).

7. MOF-guest binding energy calculation

7.1. Calculation methods.

Initial structures for the computational chemistry calculations were obtained from the cif files for each complex and the crystallographic site within, in which non-essential solvent molecules were removed. Electronic energies were calculated using the GFN2-xTB method with the application of the periodic boundary condition using the DFTB+ program. The interaction energy between the MOF and the guest was obtained using the formula: $E_{binding} = E_{MOF} + E_{guest} - (E_{MOF + guest})$ and are given in the unit of kJ mol^{-1}.

7.2. Correlation between binding energy and framework deformation.

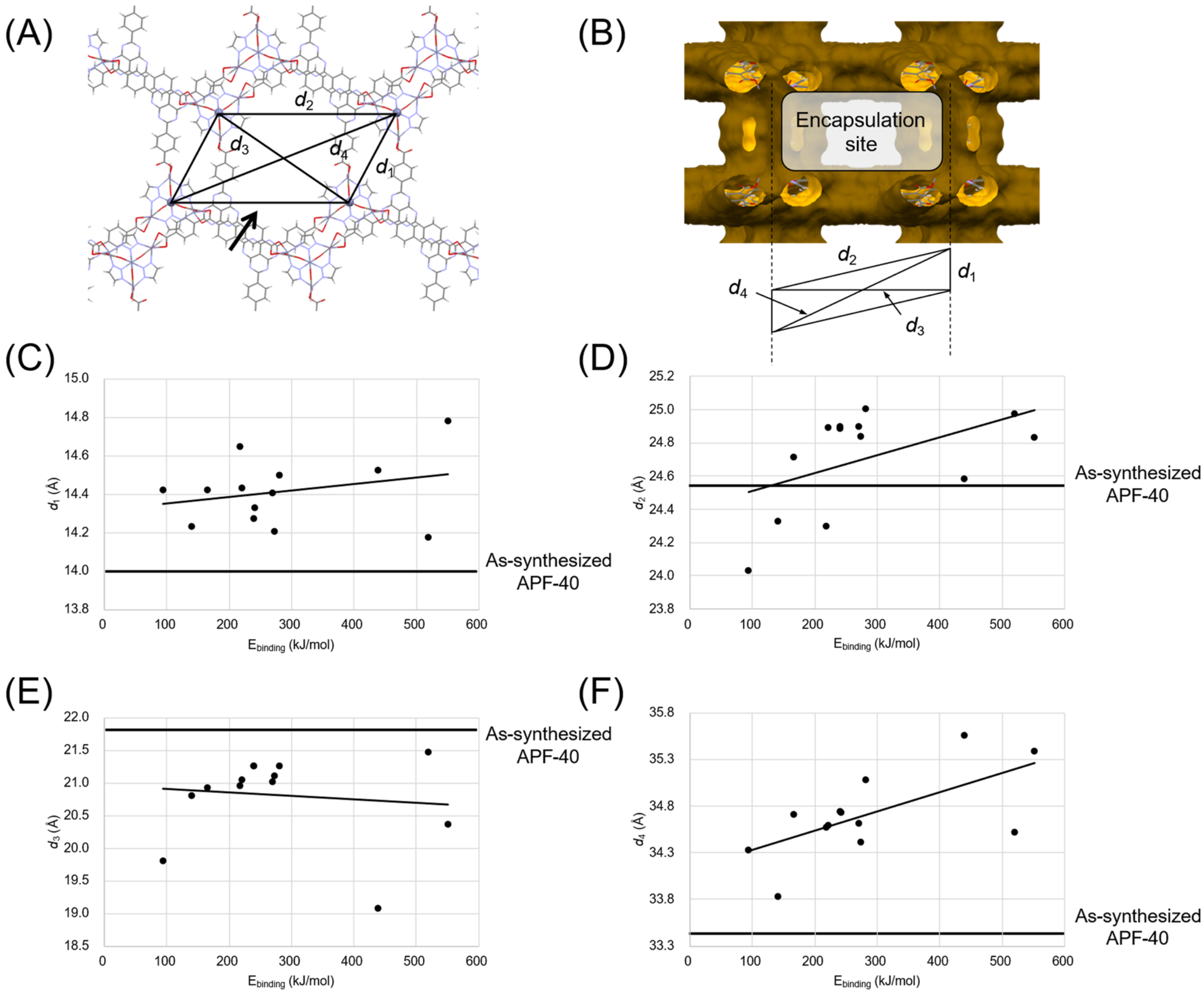

Fig. S59. Plots of host-guest binding energy in APF-40 vs distances between Zn_8 clusters of the interpenetrated nets defined by parallelogram dimensions outlining the narrow channel. (A) Structure of APF-40 showing the corresponding d_1, d_2, d_3 and d_4 distances. (B) Narrow channel of APF-40 in the direction of the black arrow in (A). The distances d_1 and d_3 correlate with the depth and width of the narrow channel of APF-40, respectively. (C–F) Plots of host-guest binding energy in APF-40 vs d_1, d_2, d_3 and d_4, respectively. Horizontal black lines show the distances in the as-synthesized APF-40.

7.3. Correlation between molecular weight of the guests and binding energy.

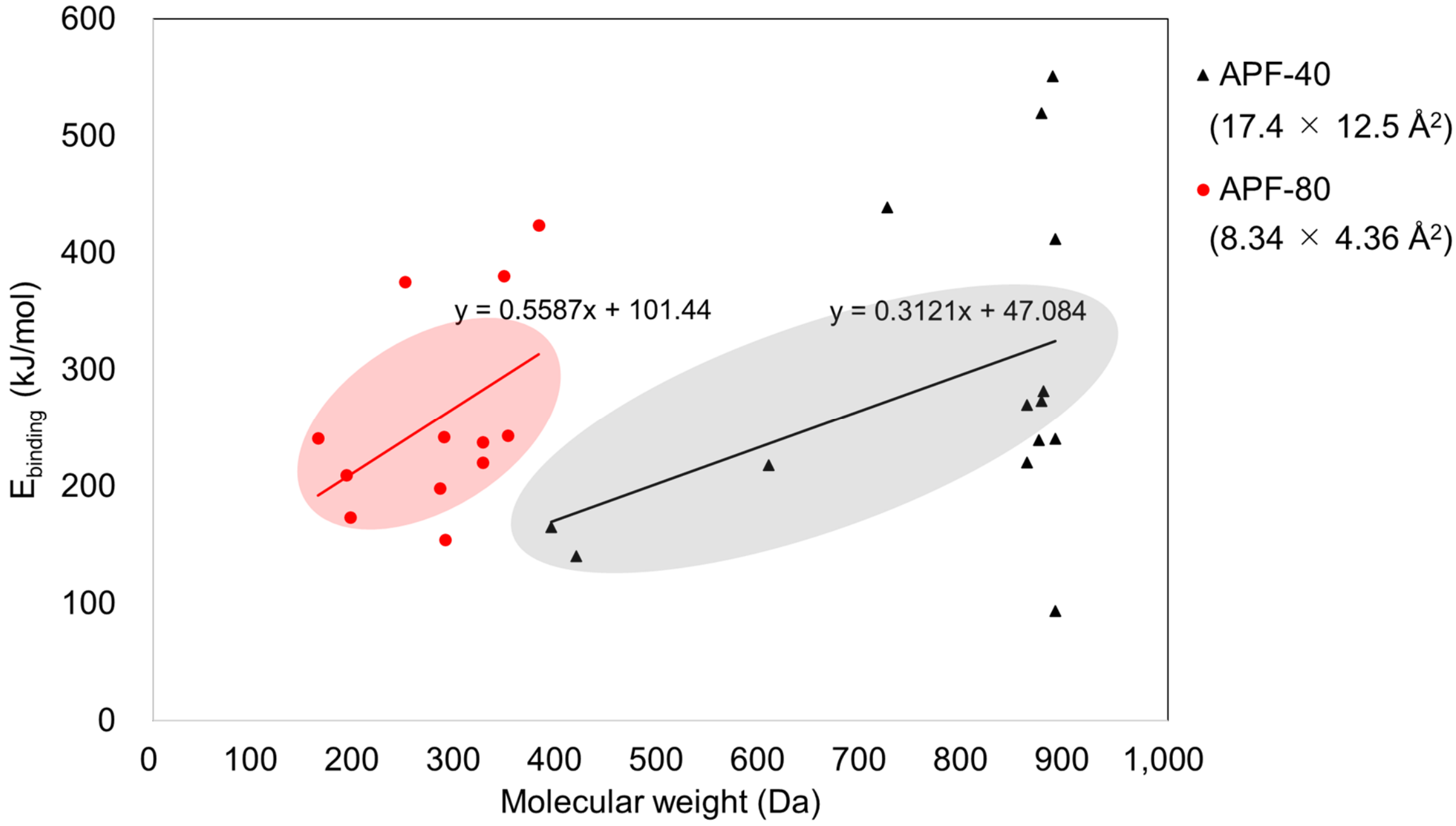

Fig. S60. Plot of guest molecular weight vs host-guest binding energy in APF-40 (*black*) and APF-80[10] (*red*).

7.4. Summary of calculated binding energies and crystallographic occupancies.

Table S5. Calculated binding energies and crystallographic occupancies for each guest site in APF-40. Structures denoted by (*) formed doubled *c*-axis supercells.

Guest name	Site	Occu. (%)	Binding energy (kJ/mol)
Spironolactone	Site Z	42.5(10)	280.1
	Site Y	64.4(9)	140.3
	Site X	25	154.7
Dexamethasone	Site Z	30	165.4
Betamethasone	Site Z	68.5(8)	551.1
Fulvestrant	Site Z	88.2(10)	218.2
Stearyl glycyrrhetinate*	Sites A and M	100	438.8
	Site B	40.3(4)	138.7
	Site C	37.5(3)	233.1
	Site D	62.5(3)	266.7
	Site F	61.5(6)	205.7
	Site G	62.5(3)	88.9
Doramectin*	Site Z	80.6(8)	94.0
	Site Y	35.8(8)	412.2
Commercial ivermectin B_{1a}	Site Z	100	519.4
	Site Y	42.7(8)	437.8
Ivermectin B_{1a}	Site Z	75.4(9)	269.9
Ivermectin B_{1b}	Site Z	65.7(6)	239.8
3′-*O*-Demethylivermectin B_{1a}	Site Z	81.5(6)	220.6
Avermectin B_{1a}	Site Z	77.7(8)	273.6
3,4-Dihydroivermectin B_{1a}	Site Z	60	281.6
Ethyl-ivermectin	Site Z	85.1(12)	240.8

8. Details about the crystallographic analysis of guest-loaded APF-40.

8.1. Visualization of crystallographic restraints and constraints.

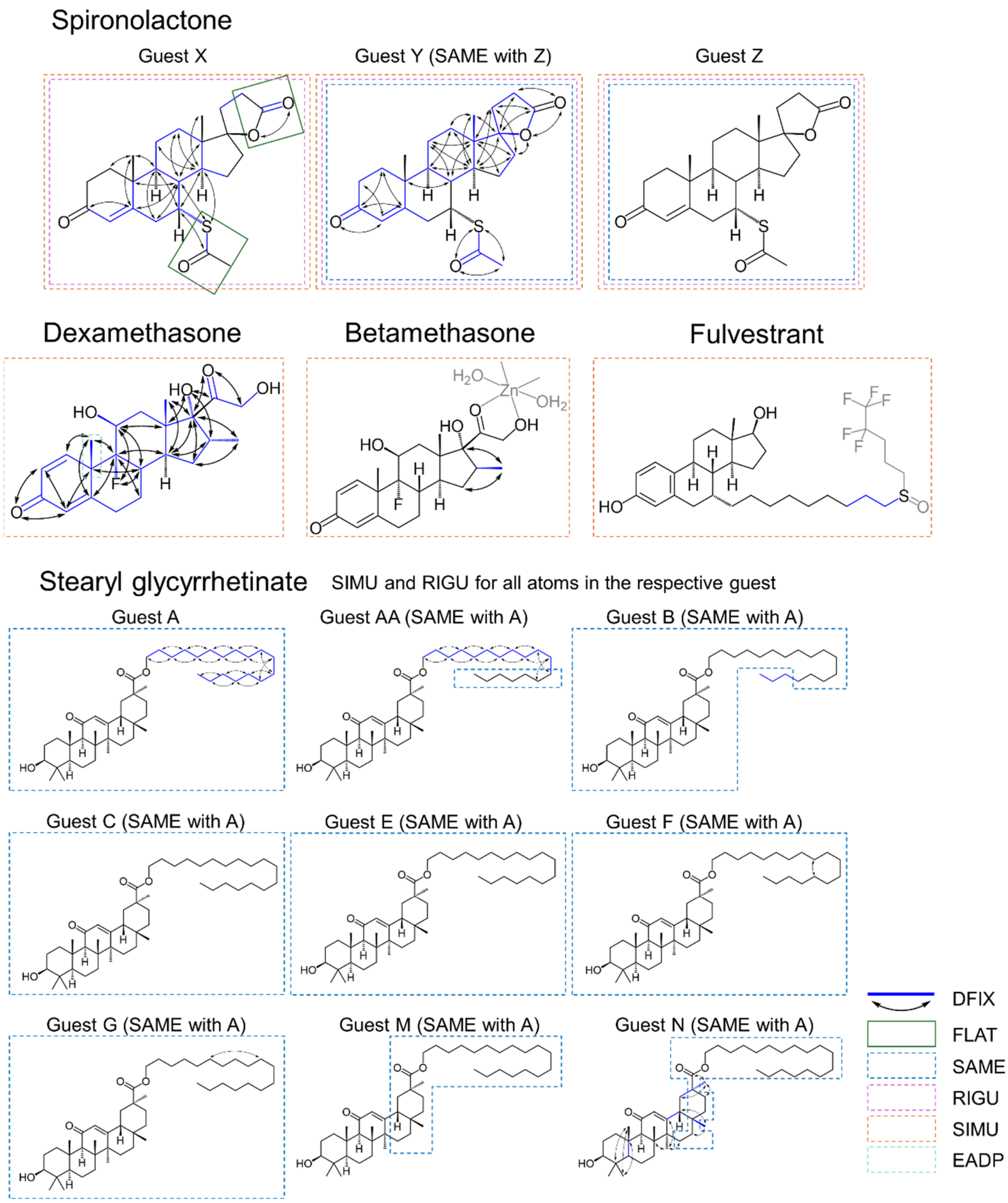

Fig. S61. Chemical structures of spironolactone, dexamethasone, betamethasone, fulvestrant and stearyl glycyrrhetinate showing restraints and constraints applied during the refinement.

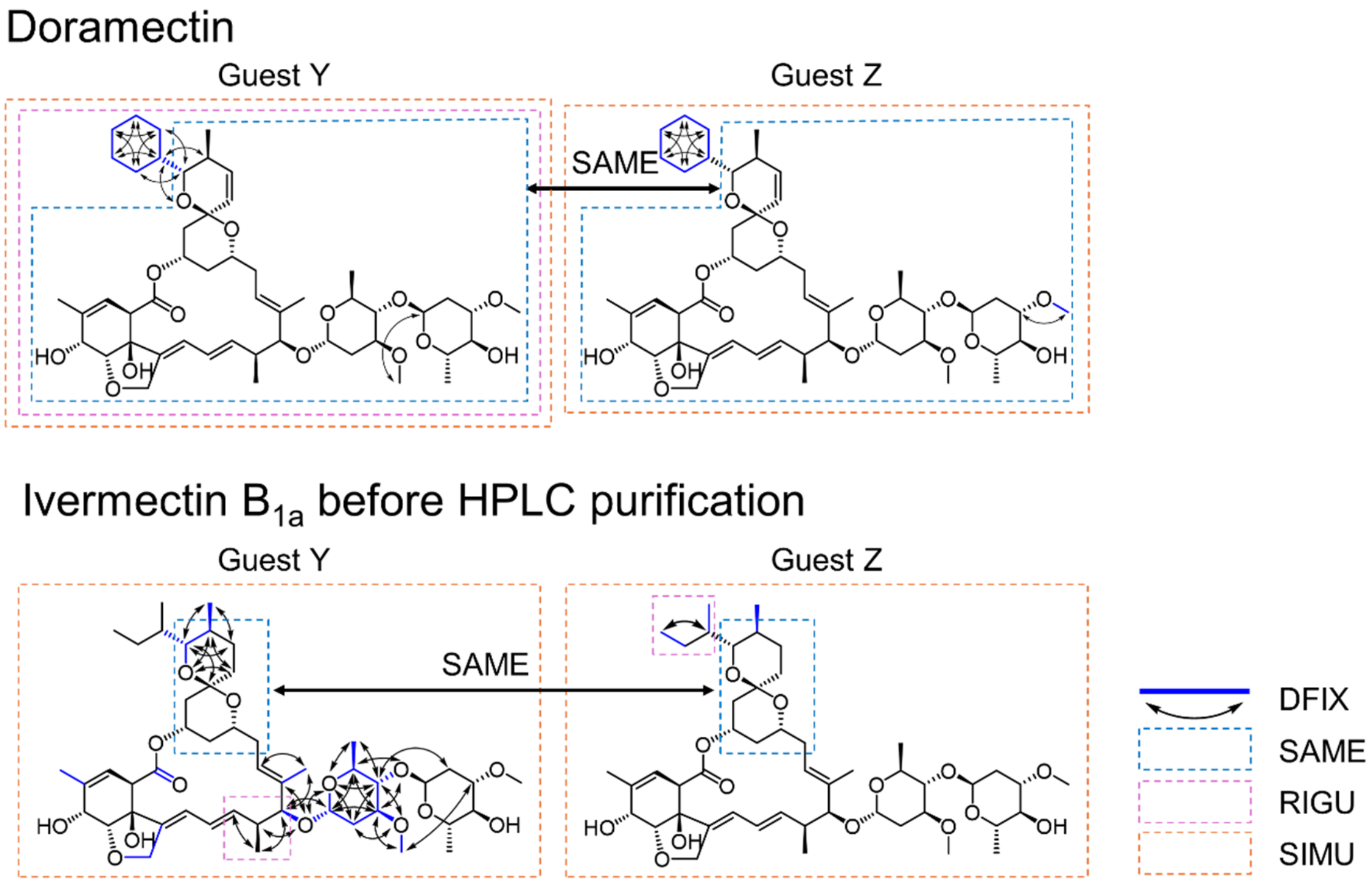

Fig. S62. Chemical structures of doramectin and ivermectin B_{1a} before HPLC purification showing restraints applied during the refinement.

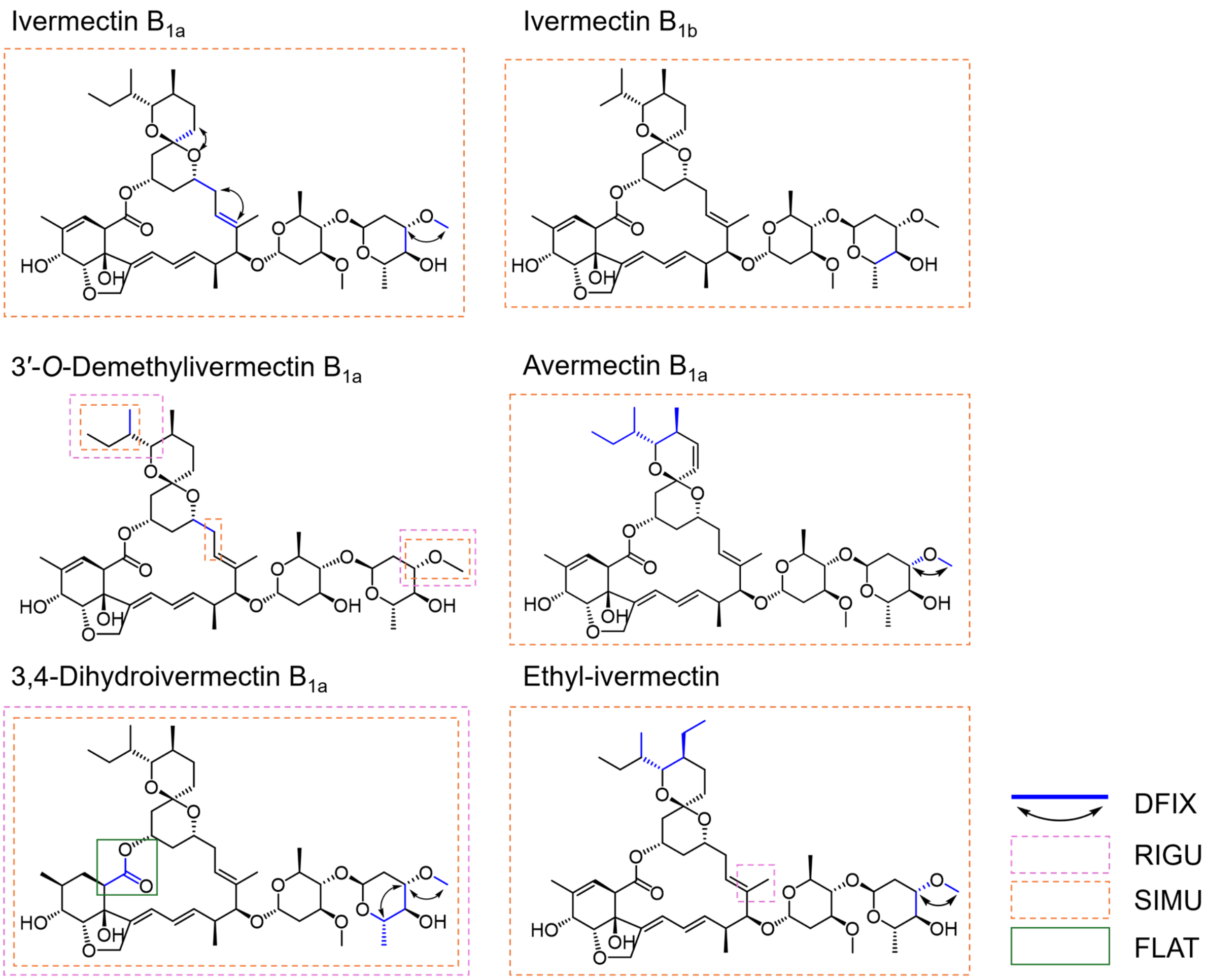

Fig. S63. Chemical structures of ivermectin B_{1a}, ivermectin B_{1b}, 3′-*O*-demethylivermectin B_{1a}, avermectin B_{1a}, 3,4-dihydroivermectin B_{1a} and ethyl-ivermectin showing restraints applied during the refinement.

8.2. Crystallographic tables.

Identification code	TBA(TTHAP)	H_4TCHAP	APF-40
Empirical formula	$C_{94}H_{128}N_{16}O_2$	$C_{34}H_{40}N_6O_{12}S_3$	$C_{100.77}H_{82.72}N_{35.59}O_{32.18}S_3Zn_8$
Formula weight	1514.12	820.90	2926.3
Temperature / K	100	95.00	95.00
Crystal system	monoclinic	monoclinic	monoclinic
Space group	$P2_1/c$	$P2_1/n$	$C2/m$
a / Å	17.2388(2)	9.955(2)	47.800(13)
b / Å	16.8586(2)	35.7180(15)	29.885(11)
c / Å	30.4811(3)	11.0620(14)	24.536(10)
α / °	90	90	90
β / °	101.8220(10)	105.728(14)	120.12(2)
γ / °	90	90	90
Volume / Å^3	8670.58(17)	3786.1(10)	30317(19)
Z	4	4	4
ρ_{calc}g / cm^3	1.160	1.440	0.641
μ / mm^{-1}	0.549	0.306	0.780
F(000)	3280.0	1720.0	5928.0
Crystal size / mm^3	0.31 × 0.27 × 0.18	0.1 × 0.06 × 0.01	0.22 × 0.13 × 0.01
Radiation	Cu Kα (λ = 1.54184)	synchrotron (λ = 0.750)	synchrotron (λ = 0.750)
2Θ range for data collection / °	6.022 to 152.814	2.406 to 71.086	2.024 to 69.062
Index ranges	$-21 \leq h \leq 15$, $-18 \leq k \leq 20$, $-38 \leq l \leq 38$	$-13 \leq h \leq 14$, $-53 \leq k \leq 54$, $-15 \leq l \leq 15$	$-72 \leq h \leq 72$, $-44 \leq k \leq 43$, $-37 \leq l \leq 37$
Reflections collected	64948	40602	308766
Independent reflections	17518 [R_{int} = 0.0288, R_{sigma} = 0.0249]	13220 [R_{int} = 0.0271, R_{sigma} = 0.0280]	50654 [R_{int} = 0.0652, R_{sigma} = 0.0436]
Data/restraints/parameters	17518/772/1219	13220/6/523	50654/606/1092
Goodness-of-fit on F^2	1.589	1.030	1.29
Final R indexes [I>=2σ (I)]	R_1 = 0.1105, wR_2 = 0.3605	R_1 = 0.0600, wR_2 = 0.1708	R_1 = 0.1159, wR_2 = 0.3552
Final R indexes [all data]	R_1 = 0.1283, wR_2 = 0.3848	R_1 = 0.0769, wR_2 = 0.1824	R_1 = 0.1948, wR_2 = 0.4013
Largest diff. peak/hole / e Å^{-3}	0.57/-0.52	0.61/-0.84	0.81/-0.63
CCDC number	2530545	2530546	2530547

Identification code	Spironolactone@APF-40	Dexamethasone@APF-40	Betamethasone@APF-40
Empirical formula	$C_{123.19}H_{103.73}N_{31.9}O_{30.84}S_{4.3}Zn_8$	$C_{204.7}H_{157.2}F_{0.6}N_{64}O_{59.8}S_6Zn_{16}$	$C_{105.74}H_{75.08}F_{0.68}N_{31.32}O_{31.7}S_3Zn_{8.34}$
Formula weight	3185.5	5720.01	2945.86
Temperature / K	95.00	95.00	95.00
Crystal system	monoclinic	monoclinic	monoclinic
Space group	*C*2	*C*2	*C*2
a / Å	47.077(15)	47.013(9)	46.524(11)
b / Å	33.855(9)	32.006(6)	31.572(5)
c / Å	24.326(10)	24.715(5)	24.832(7)
α / °	90	90	90
β / °	120.668(10)	120.39(3)	119.635(14)
γ / °	90	90	90
Volume / $Å^3$	33348(20)	32078(14)	31703(14)
Z	4	2	4
$\rho_{calc}g / cm^3$	0.634	0.592	0.617
μ / mm^{-1}	0.720	0.735	0.774
F(000)	6487.0	5786.0	5947.0
Crystal size / mm^3	0.12 × 0.09 × 0.05	0.15 × 0.05 × 0.02	0.12 × 0.05 × 0.02
Radiation	synchrotron (λ = 0.750)	synchrotron (λ = 0.750)	synchrotron (λ = 0.750)
2Θ range for data collection / °	2.538 to 55.906	2.12 to 49.838	1.726 to 48.108
Index ranges	-58 ≤ h ≤ 58, -42 ≤ k ≤ 42, -30 ≤ l ≤ 30	-52 ≤ h ≤ 52, -35 ≤ k ≤ 35, -27 ≤ l ≤ 27	-49 ≤ h ≤ 49, -34 ≤ k ≤ 34, -26 ≤ l ≤ 26
Reflections collected	223990	156754	141798
Independent reflections	63528 [R_{int} = 0.0615, R_{sigma} = 0.0532]	45026 [R_{int} = 0.0413, R_{sigma} = 0.0351]	40146 [R_{int} = 0.0397, R_{sigma} = 0.0348]
Data/restraints/parameters	63528/3375/2394	45026/1776/1689	40146/1604/1793
Goodness-of-fit on F^2	1.008	1.174	1.063
Final R indexes [I>=2σ (I)]	R_1 = 0.0997, wR_2 = 0.2530	R_1 = 0.1026, wR_2 = 0.2834	R_1 = 0.0970, wR_2 = 0.2625
Final R indexes [all data]	R_1 = 0.1846, wR_2 = 0.3154	R_1 = 0.1517, wR_2 = 0.3340	R_1 = 0.1371, wR_2 = 0.3044
Largest diff. peak/hole / e $Å^{-3}$	0.83/-0.42	0.63/-0.43	0.61/-0.54
Flack parameter	0.124(6)	0.410(6)	0.151(8)
CCDC number	2530548	2530549	2530550

Identification code	Stearyl glycyrrhetinate@APF-40	Fulvestrant@APF-40	Fulvestrant
Empirical formula	$C_{396.11}H_{472.5}N_{63.27}O_{70.45}S_6Zn_{16}$	$C_{448.83}H_{343.95}N_{124.8}O_{105.65}S_{15.53}Zn_{32}$	$C_{32}H_{47}F_5O_{3.33}S$
Formula weight	8485.35	11765.71	612.09
Temperature / K	95.00	95.00	95.00
Crystal system	monoclinic	monoclinic	trigonal
Space group	*C*2	*C*2	*R*3
a / Å	47.633(16)	47.20(2)	23.226(3)
b / Å	33.603(10)	33.108(15)	23.226(3)
c / Å	46.022(10)	24.298(17)	16.5490(15)
α / °	90	90	90
β / °	116.946(7)	120.837(13)	90
γ / °	90	90	120
Volume / Å^3	65666(33)	32603(31)	7731.3(18)
Z	4	1	9
ρ_{calc}g / cm^3	0.858	0.599	1.183
μ / mm^{-1}	0.736	0.729	0.172
F(000)	17726.0	5964.0	2940.0
Crystal size / mm^3	0.12 × 0.07 × 0.01	0.1 × 0.08 × 0.02	0.15 × 0.06 × 0.06
Radiation	synchrotron (λ = 0.750)	synchrotron (λ = 0.750)	synchrotron (λ = 0.750)
2Θ range for data collection / °	1.048 to 55.004	2.12 to 61.5	3.7 to 71.022
Index ranges	$-56 \le h \le 56, -40 \le k \le 41, -56 \le l \le 56$	$-64 \le h \le 64, -42 \le k \le 42, -26 \le l \le 26$	$-34 \le h \le 34, -34 \le k \le 34, -20 \le l \le 19$
Reflections collected	370927	250376	56114
Independent reflections	118601 [R_{int} = 0.0759, R_{sigma} = 0.0823]	72357 [R_{int} = 0.0633, R_{sigma} = 0.0579]	11690 [R_{int} = 0.0319, R_{sigma} = 0.0245]
Data/restraints/parameters	118601/14677/6611	72357/3124/1892	11690/1193/538
Goodness-of-fit on F^2	1.004	1.032	1.178
Final R indexes [I>=2σ (I)]	R_1 = 0.0939, wR_2 = 0.2353	R_1 = 0.1081, wR_2 = 0.2635	R_1 = 0.1226, wR_2 = 0.3064
Final R indexes [all data]	R_1 = 0.1807, wR_2 = 0.2872	R_1 = 0.2019, wR_2 = 0.3248	R_1 = 0.1623, wR_2 = 0.3467
Largest diff. peak/hole / e Å^{-3}	0.81/-0.44	0.76/-0.57	0.69/-0.40
Flack parameter	0.046(3)	0.031(4)	0.09(3)
CCDC number	2530724	2530551	2538982

Identification code	Doramectin@APF-40	Commercial ivermectin B_{1a}@APF-40	Ivermectin B_{1a}@APF-40
Empirical formula	$C_{233.6}H_{179.72}N_{61.6}O_{68.83}S_6Zn_{16}$	$C_{317.06}H_{318.74}N_{64}O_{100.38}S_6Zn_{16}$	$C_{246.99}H_{201.49}N_{61.3}O_{74.32}S_6Zn_{16}$
Formula weight	6189.31	7870.14	6455.63
Temperature/K	95.00	95.00	95.00
Crystal system	monoclinic	monoclinic	monoclinic
Space group	*C*2	*C*2	*C*2
a / Å	46.470(9)	47.340(9)	47.41(3)
b / Å	34.540(7)	30.700(3)	30.267(17)
c / Å	47.240(9)	24.975(4)	24.84(2)
α / °	90	90	90
β / °	117.33(3)	121.568(4)	121.102(9)
γ / °	90	90	90
Volume/Å^3	67358(28)	30926(8)	30512(35)
Z	4	2	2
ρ_{calc}g/cm^3	0.610	0.845	0.703
μ/mm^{-1}	0.704	0.782	0.779
F(000)	12557.0	8096.0	6556.0
Crystal size/mm^3	0.12 × 0.05 × 0.02	0.11 × 0.08 × 0.01	0.2 × 0.15 × 0.01
Radiation	synchrotron (λ = 0.750)	synchrotron (λ = 0.750)	synchrotron (λ = 0.750)
2Θ range for data collection/°	2.158 to 49.248	2.02 to 64.784	1.772 to 49.248
Index ranges	-51 ≤ h ≤ 51, -38 ≤ k ≤ 38, -52 ≤ l ≤ 52	-61 ≤ h ≤ 61, -43 ≤ k ≤ 43, -35 ≤ l ≤ 35	-52 ≤ h ≤ 52, -33 ≤ k ≤ 33, -25 ≤ l ≤ 25
Reflections collected	324750	302087	139683
Independent reflections	94335 [R_{int} = 0.0709, R_{sigma} = 0.0616]	85468 [R_{int} = 0.0554, R_{sigma} = 0.0488]	41079 [R_{int} = 0.0637, R_{sigma} = 0.0596]
Data/restraints/parameters	94335/7497/4093	85468/1508/2751	41079/2874/1930
Goodness-of-fit on F^2	1.164	0.983	1.230
Final R indexes [I>=2σ (I)]	R_1 = 0.1213, wR_2 = 0.3226	R_1 = 0.0931, wR_2 = 0.2632	R_1 = 0.1343, wR_2 = 0.3332
Final R indexes [all data]	R_1 = 0.2145, wR_2 = 0.3872	R_1 = 0.1679, wR_2 = 0.3303	R_1 = 0.2042, wR_2 = 0.3843
Largest diff. peak/hole / e Å^{-3}	0.77/-0.41	0.88/-0.49	0.48/-0.71
Flack parameter	0.116(5)	0.062(4)	0.153(8)
CCDC number	2530725	2530726	2530574

Identification code	Ivermectin B_{1b}@APF-40	3′-*O*-Demethylivermectin B_{1a}@APF-40	Avermectin B_{1a}@APF-40
Empirical formula	$C_{119.49}H_{97.75}N_{31.3}O_{35.3}S_3Zn_8$	$C_{128.17}H_{113.37}N_{32.03}O_{39.66}S_3Zn_8$	$C_{248.19}H_{197.62}N_{60.8}O_{77.36}S_6Zn_{16}$
Formula weight	3156.02	3356.00	6507.72
Temperature/K	95.00	95.00	95.00
Crystal system	monoclinic	monoclinic	monoclinic
Space group	*C*2	*C*2	*C*2
a / Å	46.793(9)	46.77(4)	47.124(8)
b / Å	32.513(5)	33.15(3)	30.563(10)
c / Å	24.891(7)	24.90(3)	24.888(6)
α / °	90	90	90
β / °	121.551(12)	121.495(15)	121.217(9)
γ / °	90	90	90
Volume/$Å^3$	32271(12)	32923(54)	30655(13)
Z	4	4	2
$\rho_{calc}g/cm^3$	0.650	0.677	0.705
μ/mm^{-1}	0.735	0.724	0.776
F(000)	6417.0	6847.0	6614.0
Crystal size/mm^3	0.24 × 0.18 × 0.01	0.15 × 0.08 × 0.01	0.2 × 0.15 × 0.01
Radiation	synchrotron (λ = 0.750)	synchrotron (λ = 0.750)	synchrotron (λ = 0.750)
2Θ range for data collection/°	2.026 to 48.672	1.686 to 50.444	2.226 to 49.248
Index ranges	$-51 \le h \le 51, -35 \le k \le 35, -27 \le l \le 27$	$-53 \le h \le 53, -37 \le k \le 37, -28 \le l \le 28$	$-52 \le h \le 52, -33 \le k \le 33, -27 \le l \le 27$
Reflections collected	146131	166616	144438
Independent reflections	42860 [R_{int} = 0.0354, R_{sigma} = 0.0324]	50267 [R_{int} = 0.0516, R_{sigma} = 0.0478]	41821 [R_{int} = 0.0585, R_{sigma} = 0.0524]
Data/restraints/parameters	42860/808/2018	50267/404/2069	41821/1429/1998
Goodness-of-fit on F^2	1.058	1.042	1.015
Final R indexes [I>=2σ (I)]	R_1 = 0.0948, wR_2 = 0.2544	R_1 = 0.0903, wR_2 = 0.2470	R_1 = 0.1043, wR_2 = 0.2731
Final R indexes [all data]	R_1 = 0.1346, wR_2 = 0.2944	R_1 = 0.1333, wR_2 = 0.2815	R_1 = 0.1800, wR_2 = 0.3359
Largest diff. peak/hole / e $Å^{-3}$	0.61/-0.39	0.81/-0.52	0.55/-0.35
Flack parameter	0.117(5)	0.109(5)	0.093(6)
CCDC number	2530575	2530576	2530577

Identification code	3,4-Dihydroivermectin B_{1a}@APF-40	Ethyl-ivermectin@APF-40
Empirical formula	$C_{236.4}H_{199.2}N_{63.6}O_{69.7}S_6Zn_{16}$	$C_{259.01}H_{222.66}N_{61.8}O_{79.07}S_6Zn_{16}$
Formula weight	6284.46	6704.30
Temperature / K	95.00	95.00
Crystal system	monoclinic	monoclinic
Space group	*C*2	*C*2
a / Å	46.88(3)	47.21(3)
b / Å	31.956(19)	31.07(3)
c / Å	25.006(15)	24.896(14)
α / °	90	90
β / °	121.529(15)	121.337(13)
γ / °	90	90
Volume / Å^3	31931(34)	31190(37)
Z	2	2
ρ_{calc}g / cm^3	0.654	0.714
μ / mm^{-1}	0.743	0.764
F(000)	6393.0	6836.0
Crystal size / mm^3	0.08 × 0.06 × 0.01	0.08 × 0.06 × 0.01
Radiation	synchrotron (λ = 0.750)	synchrotron (λ = 0.750)
2Θ range for data collection / °	2.016 to 44.048	1.746 to 40.634
Index ranges	-46 ≤ h ≤ 46, -31 ≤ k ≤ 31, -24 ≤ l ≤ 24	-43 ≤ h ≤ 43, -28 ≤ k ≤ 28, -23 ≤ l ≤ 23
Reflections collected	112898	87799
Independent reflections	32781 [R_{int} = 0.0598, R_{sigma} = 0.0514]	25787 [R_{int} = 0.1047, R_{sigma} = 0.0938]
Data/restraints/parameters	32781/2521/2052	25787/2978/2052
Goodness-of-fit on F^2	1.050	1.121
Final R indexes [I>=2σ (I)]	R_1 = 0.0989, wR_2 = 0.2634	R_1 = 0.1210, wR_2 = 0.2939
Final R indexes [all data]	R_1 = 0.1543, wR_2 = 0.3073	R_1 = 0.1887, wR_2 = 0.3430
Largest diff. peak/hole / e Å^{-3}	0.53/-0.40	0.74/-0.59
Flack parameter	0.108(7)	0.152(12)
CCDC number	2530578	2530579

8.3. A or B- level checkcif alerts and their responses.

CCDC 2530545 (TBA(TTHAP))

_vrf_PLAT084_tbatthap
;
PROBLEM: High wR2 Value (i.e. > 0.25) .... 0.38 Report
RESPONSE: This alert is a consequence of the relatively low intensity of the diffraction due to the flexible butyl group in the cation, which cause the severe disorder.
;
_vrf_PLAT934_tbatthap
;
PROBLEM: Number of (Iobs-Icalc)/Sigma(W) > 10 Outliers .. 6 Check
RESPONSE:The relatively large number of outliers is attributed to diffuse electron density from disordered solvent DMF molecules and tetrabutyl ammonium ions within the pores that could not be adequately modeled. This is a common feature in porous organic crystal structures where some residual electron density remains unmodeled due to severe disorder. The outliers do not affect the quality of the structure determination for the well-ordered portions, including the guest molecule.
;

CCDC 2530546 (H_4TCHAP)

No alerts.

CCDC 2530547 (APF-40)

_vrf_PLAT602_apf-40
;
PROBLEM: Solvent Accessible VOID(S) in the Unit-Cell .... ! Check
RESPONSE: We assigned as many solvent molecules as possible in the pore.
However, several solvent molecules could not be modeled due to severe disorder in the large pore of the MOF.
;
_vrf_PLAT934_apf-40
;
PROBLEM: Number of (Iobs-Icalc)/Sigma(W) > 10 Outliers .. 24 Check
RESPONSE: In the low-angle region, multiple diffraction peaks overlapped due to the large unit cell parameters of the MOF.
Furthermore, during omega scan measurements, several low-angle reflections were obscured by the shadow of the beamline beamstop.
;
_vrf_PLAT049_apf-40
;
PROBLEM: Calculated Density Less Than 1.0 gcm-3 .... 0.6411 Check
RESPONSE: This alert is related to PLAT602_ALERT_2_A.

In this analysis, the crystal density appears small due to
the presence of voids that should be occupied by solvent molecules.
;
_vrf_PLAT084_apf-40
;
PROBLEM: High wR2 Value (i.e. > 0.25) .... 0.40 Report
RESPONSE: This alert is a consequence of the relatively low intensity of
the diffraction due to the highly porous structure of the MOF.
;
_vrf_PLAT430_apf-40
;
PROBLEM: Short Inter D...A Contact O1Z ..O6H . 2.77 Ang.
RESPONSE: These interactions should be hydrogen bonds.
However, the hydrogen atoms of waters could not be modeled due to
disorder, these alerts are inevitable.
;

CCDC 2530548 (Spironolactone@APF-40)

_vrf_PLAT029_spironolactone
;
PROBLEM: _diffrn_measured_fraction_theta_full value Low . 0.939 Why?
RESPONSE: In this measurement, the crystal was measured using
omega scan. Omega scan measurement provided the best data
quality and it is suitable for this research.
However, diffraction data set of this crystal was slightly
not enough to cover full sphere.
;
_vrf_PLAT049_spironolactone
;
PROBLEM: Calculated Density Less Than 1.0 gcm-3 .... 0.6358 Check
RESPONSE: This alert is related to PLAT602_ALERT_2_A.
In this analysis, the crystal density appears small due to
the presence of voids that should be occupied by solvent molecules.
;
_vrf_PLAT341_spironolactone
;
PROBLEM: Low Bond Precision on C-C Bonds .... 0.028 Ang.
RESPONSE: Since the MOF formed the highly porous framework,
the partial structures of the framework that did not form clear
interactions with the guest molecules had severe disorder.
;
_vrf_PLAT602_spironolactone
;
PROBLEM: Solvent Accessible VOID(S) in the Unit-Cell .... ! Check
RESPONSE: We assigned as many solvent molecules
as possible in the pore.

However, several solvent molecules could not be modeled due to
severe disorder in the large pore of the MOF.
;

CCDC 2530549 (Dexamethasone@APF-40)

_vrf_PLAT029_dexamethasone
;
PROBLEM: _diffrn_measured_fraction_theta_full value Low .   0.951 Why?
RESPONSE: In this measurement, the crystal was measured using
omega scan. Omega scan measurement provided the best data
quality and it is suitable for this research.
However, diffraction data set of this crystal was slightly
not enough to cover full sphere.
;
_vrf_PLAT049_dexamethasone
;
PROBLEM: Calculated Density Less Than 1.0 gcm-3 ....   0.5922 Check
RESPONSE: This alert is related to PLAT602_ALERT_2_A.
In this analysis, the crystal density appears small due to
the presence of voids that should be occupied by solvent molecules.
;
_vrf_PLAT306_dexamethasone
;
PROBLEM: Isolated Oxygen Atom (H-atoms Missing ?) ....   O6W Check
RESPONSE: These oxygen atoms are components of the water in the pore of
the framework. Since it was difficult to determine static position
of the hydrogen atoms of the water without clear interactions,
the model has no hydrogens.
;
_vrf_PLAT341_dexamethasone
;
PROBLEM: Low Bond Precision on C-C Bonds ....   0.03453 Ang.
RESPONSE: Since the MOF formed the highly porous framework,
the partial structures of the framework that did not form clear
interactions with the guest molecules had severe disorder.
;
_vrf_PLAT430_dexamethasone
;
PROBLEM: Short Inter D...A Contact O6W   ..O4Z   .   2.68 Ang.
RESPONSE: These interactions should be hydrogen bonds.
However, the hydrogen atoms of waters could not be modeled due to
disorder, these alerts are inevitable.
;
_vrf_PLAT602_dexamethasone
;
PROBLEM: Solvent Accessible VOID(S) in the Unit-Cell ....   ! Check

RESPONSE: We assigned as many solvent molecules
as possible in the pore.
However, several solvent molecules could not be modeled due to
severe disorder in the large pore of the MOF.
;
_vrf_PLAT934_dexamethasone
;
PROBLEM: Number of (Iobs-Icalc)/Sigma(W) > 10 Outliers .. 4 Check
RESPONSE: In the low-angle region, multiple diffraction
peaks overlapped due to the large unit cell parameters of the MOF.
Furthermore, during omega scan measurements, several low-angle
reflections were obscured by the shadow of the beamline beamstop.
;
_vrf_THETM01_dexamethasone
;
PROBLEM: The value of sine(theta_max)/wavelength is less than 0.575
RESPONSE: A full set of data was collected.
However, the crystal diffracted very weakly in the high angle range
beyond d value of 0.89 angstrom due to the highly porous
structure of the MOF.
;

CCDC 2530550 (Betamethasone@APF-40)
_vrf_THETM01_betamethasone
;
PROBLEM: The value of sine(theta_max)/wavelength is less than 0.550
RESPONSE: A full set of data was collected.
However, the crystal diffracted very weakly in the high angle range
beyond d value of 0.92 angstrom due to the highly porous
structure of the MOF.
;
_vrf_PLAT602_betamethasone
;
PROBLEM: Solvent Accessible VOID(S) in the Unit-Cell .... ! Check
RESPONSE: We assigned as many solvent molecules
as possible in the pore.
However, several solvent molecules could not be modeled due to
severe disorder in the large pore of the MOF.
;
_vrf_PLAT934_betamethasone
;
PROBLEM: Number of (Iobs-Icalc)/Sigma(W) > 10 Outliers .. 16 Check
RESPONSE: In the low-angle region, multiple diffraction
peaks overlapped due to the large unit cell parameters of the MOF.
Furthermore, during omega scan measurements, several low-angle
reflections were obscured by the shadow of the beamline beamstop.

;
_vrf_PLAT029_betamethasone
;
PROBLEM: _diffrn_measured_fraction_theta_full value Low . 0.945 Why?
RESPONSE: In this measurement, the crystal was measured using
omega scan. Omega scan measurement provided the best data
quality and it is suitable for this research.
However, diffraction data set of this crystal was slightly
not enough to cover full sphere.
;
_vrf_PLAT049_betamethasone
;
PROBLEM: Calculated Density Less Than 1.0 gcm-3 .... 0.6175 Check
RESPONSE: This alert is related to PLAT602_ALERT_2_A.
In this analysis, the crystal density appears small due to
the presence of voids that should be occupied by solvent molecules.
;
_vrf_PLAT341_betamethasone
;
PROBLEM: Low Bond Precision on C-C Bonds .... 0.03053 Ang.
RESPONSE: Since the MOF formed the highly porous framework,
the partial structures of the framework that did not form clear
interactions with the guest molecules had severe disorder.
;
_vrf_PLAT430_betamethasone
;
PROBLEM: Short Inter D...A Contact O4W ..O10Z . 2.82 Ang.
RESPONSE: These interactions should be hydrogen bonds.
However, the hydrogen atoms of waters could not be modeled due to
disorder, these alerts are inevitable.
;

CCDC 2530724 (Stearyl glycyrrhetinate@APF-40)
_vrf_PLAT049_stearyl
;
PROBLEM: Calculated Density Less Than 1.0 gcm-3 .... 0.8504 Check
RESPONSE: This alert is related to PLAT602_ALERT_2_A.
In this analysis, the crystal density appears small due to
the presence of voids that should be occupied by solvent molecules.
;
_vrf_PLAT306_stearyl
;
PROBLEM: Isolated Oxygen Atom(s) (H-atoms Missing ?) .... 3 Check
RESPONSE: These oxygen atoms are components of the water in the pore of
the framework. Since it was difficult to determine static position
of the hydrogen atoms of the water without clear interactions,

the model has no hydrogens.
;
_vrf_PLAT341_stearyl
;
PROBLEM: Low Bond Precision on C-C Bonds .... 0.02375 Ang.
RESPONSE: Since the MOF formed the highly porous framework,
the partial structures of the framework that did not form clear
interactions with the guest molecules had severe disorder.
;
_vrf_PLAT430_stearyl
;
PROBLEM: Short Inter D...A Contact O1W ..O23X . 2.72 Ang.
RESPONSE: These interactions should be hydrogen bonds.
However, the hydrogen atoms of waters could not be modeled due to
disorder, these alerts are inevitable.
;
_vrf_PLAT602_stearyl
;
PROBLEM: Solvent Accessible VOID(S) in the Unit-Cell .... ! Check
RESPONSE: We assigned as many solvent molecules
as possible in the pore.
However, several solvent molecules could not be modeled due to
severe disorder in the large pore of the MOF.
;
_vrf_PLAT934_stearyl
;
PROBLEM: Number of (Iobs-Icalc)/Sigma(W) > 10 Outliers .. 2 Check
RESPONSE: In the low-angle region, multiple diffraction
peaks overlapped due to the large unit cell parameters of the MOF.
Furthermore, during omega scan measurements, several low-angle
reflections were obscured by the shadow of the beamline beamstop.
;

CCDC 2530551 (Fulvestrant@APF-40)
_vrf_PLAT029_fulvestrant
;
PROBLEM: _diffrn_measured_fraction_theta_full value Low . 0.937 Why?
RESPONSE: In this measurement, the crystal was measured using
omega scan. Omega scan measurement provided the best data
quality and it is suitable for this research.
However, diffraction data set of this crystal was slightly
not enough to cover full sphere.
;
_vrf_PLAT049_fulvestrant
;
PROBLEM: Calculated Density Less Than 1.0 gcm-3 .... 0.5993 Check

RESPONSE: This alert is related to PLAT602_ALERT_2_A.
In this analysis, the crystal density appears small due to
the presence of voids that should be occupied by solvent molecules.
;
_vrf_PLAT341_fulvestrant
;
PROBLEM: Low Bond Precision on C-C Bonds .... 0.02473 Ang.
RESPONSE: Since the MOF formed the highly porous framework,
the partial structures of the framework that did not form clear
interactions with the guest molecules had severe disorder.
;
_vrf_PLAT430_fulvestrant
;
PROBLEM: Short Inter D...A Contact O11Z ..N6B . 2.84 Ang.
RESPONSE: These interactions should be hydrogen bonds.
However, the hydrogen atoms of waters could not be modeled due to
disorder, these alerts are inevitable.
;
_vrf_PLAT602_fulvestrant
;
PROBLEM: Solvent Accessible VOID(S) in the Unit-Cell .... ! Check
RESPONSE: We assigned as many solvent molecules
as possible in the pore.
However, several solvent molecules could not be modeled due to
severe disorder in the large pore of the MOF.
;
_vrf_PLAT934_fulvestrant
;
PROBLEM: Number of (Iobs-Icalc)/Sigma(W) > 10 Outliers .. 15 Check
RESPONSE: In the low-angle region, multiple diffraction
peaks overlapped due to the large unit cell parameters of the MOF.
Furthermore, during omega scan measurements, several low-angle
reflections were obscured by the shadow of the beamline beamstop.
;

CCDC 2538982 (Fulvestrant)
No alerts.

CCDC 2530725 (Doramectin@APF-40)
_vrf_THETM01_doramectin
;
PROBLEM: The value of sine(theta_max)/wavelength is less than 0.575
RESPONSE: A full set of data was collected.
However, the crystal diffracted very weakly in the high angle range
beyond d value of 0.90 angstrom due to the highly porous
structure of the MOF.

;

_vrf_PLAT602_doramectin
;
PROBLEM: Solvent Accessible VOID(S) in the Unit-Cell .... ! Check
RESPONSE: We assigned as many solvent molecules
as possible in the pore.
However, several solvent molecules could not be modeled due to
severe disorder in the large pore of the MOF.
;

_vrf_PLAT934_doramectin
;
PROBLEM: Number of (Iobs-Icalc)/Sigma(W) > 10 Outliers .. 24 Check
RESPONSE: In the low-angle region, multiple diffraction
peaks overlapped due to the large unit cell parameters of the MOF.
Furthermore, during omega scan measurements, several low-angle
reflections were obscured by the shadow of the beamline beamstop.
;

_vrf_PLAT049_doramectin
;
PROBLEM: Calculated Density Less Than 1.0 gcm-3 .... 0.6100 Check
RESPONSE: This alert is related to PLAT602_ALERT_2_A.
In this analysis, the crystal density appears small due to
the presence of voids that should be occupied by solvent molecules.
;

_vrf_PLAT084_doramectin
;
PROBLEM: High wR2 Value (i.e. > 0.25) .... 0.39 Report
RESPONSE: This alert is a consequence of the relatively low intensity of
the diffraction due to the highly porous structure of the MOF.
;

_vrf_PLAT341_doramectin
;
PROBLEM: Low Bond Precision on C-C Bonds .... 0.04164 Ang.
RESPONSE: Since the MOF formed the highly porous framework,
the partial structures of the framework that did not form clear
interactions with the guest molecules had severe disorder.
;

_vrf_PLAT430_doramectin
;
PROBLEM: Short Inter D...A Contact O1W ..O51Z . 2.64 Ang.

RESPONSE: These interactions should be hydrogen bonds.
However, the hydrogen atoms of waters could not be modeled due to
disorder, these alerts are inevitable.
;

CCDC 2530726 (Commercial ivermectin B_{1a}@APF-40)
_vrf_PLAT029_impureivermectin
;
PROBLEM: _diffrn_measured_fraction_theta_full value Low . 0.934 Why?
RESPONSE: In this measurement, the crystal was measured using
omega scan. Omega scan measurement provided the best data
quality and it is suitable for this research.
However, diffraction data set of this crystal was slightly
not enough to cover full sphere.
;
_vrf_PLAT602_impureivermectin
;
PROBLEM: Solvent Accessible VOID(S) in the Unit-Cell .... ! Check
RESPONSE: We assigned as many solvent molecules
as possible in the pore.
However, several solvent molecules could not be modeled due to
severe disorder in the large pore of the MOF.
;
_vrf_PLAT934_impureivermectin
;
PROBLEM: Number of (Iobs-Icalc)/Sigma(W) > 10 Outliers .. 16 Check
RESPONSE: In the low-angle region, multiple diffraction
peaks overlapped due to the large unit cell parameters of the MOF.
Furthermore, during omega scan measurements, several low-angle
reflections were obscured by the shadow of the beamline beamstop.
;
_vrf_PLAT049_impureivermectin
;
PROBLEM: Calculated Density Less Than 1.0 gcm-3 .... 0.8516 Check
RESPONSE: This alert is related to PLAT602_ALERT_2_A.
In this analysis, the crystal density appears small due to
the presence of voids that should be occupied by solvent molecules.
;
_vrf_PLAT341_impureivermectin
;
PROBLEM: Low Bond Precision on C-C Bonds .... 0.02335 Ang.
RESPONSE: Since the MOF formed the highly porous framework,
the partial structures of the framework that did not form clear
interactions with the guest molecules had severe disorder.
;
_vrf_PLAT430_impureivermectin

;
PROBLEM: Short Inter D...A Contact O5W ..O34A . 2.57 Ang.
RESPONSE: These interactions should be hydrogen bonds.
However, the hydrogen atoms of waters could not be modeled due to
disorder, these alerts are inevitable.
;

CCDC 2530574 (Ivermectin B_{1a}@APF-40)
_vrf_PLAT029_Ivermectin_B1a
;
PROBLEM: _diffrn_measured_fraction_theta_full value Low . 0.946 Why?
RESPONSE: In this measurement, the crystal was measured using
omega scan. Omega scan measurement provided the best data
quality and it is suitable for this research.
However, diffraction data set of this crystal was slightly
not enough to cover full sphere.
;
_vrf_PLAT049_Ivermectin_B1a
;
PROBLEM: Calculated Density Less Than 1.0 gcm-3 .... 0.7025 Check
RESPONSE: This alert is related to PLAT602_ALERT_2_A.
In this analysis, the crystal density appears small due to
the presence of voids that should be occupied by solvent molecules.
;
_vrf_PLAT084_Ivermectin_B1a
;
PROBLEM: High wR2 Value (i.e. > 0.25) .... 0.38 Report
RESPONSE: This alert is a consequence of the relatively low intensity of
the diffraction due to the highly porous structure of the MOF.
;
_vrf_PLAT341_Ivermectin_B1a
;
PROBLEM: Low Bond Precision on C-C Bonds .... 0.04093 Ang.
RESPONSE: Since the MOF formed the highly porous framework,
the partial structures of the framework that did not form clear
interactions with the guest molecules had severe disorder.
;
_vrf_PLAT430_Ivermectin_B1a
;
PROBLEM: Short Inter D...A Contact O1W ..O23A . 2.73 Ang.
RESPONSE: These interactions should be hydrogen bonds.
However, the hydrogen atoms of waters could not be modeled due to
disorder, these alerts are inevitable.
;
_vrf_PLAT602_Ivermectin_B1a
;

PROBLEM: Solvent Accessible VOID(S) in the Unit-Cell .... ! Check
RESPONSE: We assigned as many solvent molecules
as possible in the pore.
However, several solvent molecules could not be modeled due to
severe disorder in the large pore of the MOF.
;
_vrf_PLAT934_Ivermectin_B1a
;
PROBLEM: Number of (Iobs-Icalc)/Sigma(W) > 10 Outliers .. 6 Check
RESPONSE: In the low-angle region, multiple diffraction
peaks overlapped due to the large unit cell parameters of the MOF.
Furthermore, during omega scan measurements, several low-angle
reflections were obscured by the shadow of the beamline beamstop.
;

_vrf_THETM01_Ivermectin_B1a
;
PROBLEM: The value of sine(theta_max)/wavelength is less than 0.575
RESPONSE: A full set of data was collected.
However, the crystal diffracted very weakly in the high angle range
beyond d value of 0.90 angstrom due to the highly porous
structure of the MOF.
;

CCDC 2530575 (Ivermectin B_{1b}@APF-40)
_vrf_PLAT049_Ivermectin_B1b
;
PROBLEM: Calculated Density Less Than 1.0 gcm-3 .... 0.6496 Check
RESPONSE: This alert is related to PLAT602_ALERT_2_A.
In this analysis, the crystal density appears small due to
the presence of voids that should be occupied by solvent molecules.
;
_vrf_PLAT341_Ivermectin_B1b
;
PROBLEM: Low Bond Precision on C-C Bonds .... 0.03025 Ang.
RESPONSE: Since the MOF formed the highly porous framework,
the partial structures of the framework that did not form clear
interactions with the guest molecules had severe disorder.
;
_vrf_PLAT430_Ivermectin_B1b
;
PROBLEM: Short Inter D...A Contact O1W ..N4B . 2.80 Ang.
RESPONSE: These interactions should be hydrogen bonds.
However, the hydrogen atoms of waters could not be modeled due to
disorder, these alerts are inevitable.
;

_vrf_PLAT602_Ivermectin_B1b
;
PROBLEM: Solvent Accessible VOID(S) in the Unit-Cell .... ! Check
RESPONSE: We assigned as many solvent molecules
as possible in the pore.
However, several solvent molecules could not be modeled due to
severe disorder in the large pore of the MOF.
;
_vrf_PLAT934_Ivermectin_B1b
;
PROBLEM: Number of (Iobs-Icalc)/Sigma(W) > 10 Outliers .. 10 Check
RESPONSE: In the low-angle region, multiple diffraction
peaks overlapped due to the large unit cell parameters of the MOF.
Furthermore, during omega scan measurements, several low-angle
reflections were obscured by the shadow of the beamline beamstop.
;

_vrf_THETM01_Ivermectin_B1b
;
PROBLEM: The value of sine(theta_max)/wavelength is less than 0.550
RESPONSE: A full set of data was collected.
However, the crystal diffracted very weakly in the high angle range
beyond d value of 0.91 angstrom due to the highly porous
structure of the MOF.
;

CCDC 2530576 (3′-*O*-Demethylivermectin B_{1a}@APF-40)

_vrf_PLAT049_3-O-Demethyl_ivermectin_B1a
;
PROBLEM: Calculated Density Less Than 1.0 gcm-3 .... 0.6779 Check
RESPONSE: This alert is related to PLAT602_ALERT_2_A.
In this analysis, the crystal density appears small due to
the presence of voids that should be occupied by solvent molecules.
;
_vrf_PLAT306_3-O-Demethyl_ivermectin_B1a
;
PROBLEM: Isolated Oxygen Atom (H-atoms Missing ?) .... O3W Check
RESPONSE: These oxygen atoms are components of the water in the pore of
the framework. Since it was difficult to determine static position
of the hydrogen atoms of the water without clear interactions,
the model has no hydrogens.
;
_vrf_PLAT341_3-O-Demethyl_ivermectin_B1a
;
PROBLEM: Low Bond Precision on C-C Bonds .... 0.02699 Ang.
RESPONSE: Since the MOF formed the highly porous framework,

the partial structures of the framework that did not form clear
interactions with the guest molecules had severe disorder.
;
_vrf_PLAT430_3-O-Demethyl_ivermectin_B1a
;
PROBLEM: Short Inter D...A Contact O3W ..O16A . 2.70 Ang.
RESPONSE: These interactions should be hydrogen bonds.
However, the hydrogen atoms of waters could not be modeled due to
disorder, these alerts are inevitable.
;
_vrf_PLAT602_3-O-Demethyl_ivermectin_B1a
;
PROBLEM: Solvent Accessible VOID(S) in the Unit-Cell .... ! Check
RESPONSE: We assigned as many solvent molecules
as possible in the pore.
However, several solvent molecules could not be modeled due to
severe disorder in the large pore of the MOF.
;
_vrf_PLAT934_3-O-Demethyl_ivermectin_B1a
;
PROBLEM: Number of (Iobs-Icalc)/Sigma(W) > 10 Outliers .. 23 Check
RESPONSE: In the low-angle region, multiple diffraction
peaks overlapped due to the large unit cell parameters of the MOF.
Furthermore, during omega scan measurements, several low-angle
reflections were obscured by the shadow of the beamline beamstop.
;
_vrf_THETM01_3-O-Demethyl_ivermectin_B1a
;
PROBLEM: The value of sine(theta_max)/wavelength is less than 0.575
RESPONSE: A full set of data was collected.
However, the crystal diffracted very weakly in the high angle range
beyond d value of 0.88 angstrom due to the highly porous
structure of the MOF.
;

CCDC 2530577 (Avermectin B_{1a}@APF-40)
_vrf_PLAT029_Avermectin_B1a
;
PROBLEM: _diffrn_measured_fraction_theta_full value Low . 0.952 Why?
RESPONSE: In this measurement, the crystal was measured using
omega scan. Omega scan measurement provided the best data
quality and it is suitable for this research.
However, diffraction data set of this crystal was slightly
not enough to cover full sphere.
;
_vrf_PLAT049_Avermectin_B1a

;
PROBLEM: Calculated Density Less Than 1.0 gcm-3 .... 0.7053 Check
RESPONSE: This alert is related to PLAT602_ALERT_2_A.
In this analysis, the crystal density appears small due to
the presence of voids that should be occupied by solvent molecules.
;
_vrf_PLAT341_Avermectin_B1a
;
PROBLEM: Low Bond Precision on C-C Bonds .... 0.042 Ang.
RESPONSE: Since the MOF formed the highly porous framework,
the partial structures of the framework that did not form clear
interactions with the guest molecules had severe disorder.
;
_vrf_PLAT430_Avermectin_B1a
;
PROBLEM: Short Inter D...A Contact O8W ..O23A . 2.69 Ang.
RESPONSE: These interactions should be hydrogen bonds.
However, the hydrogen atoms of waters could not be modeled due to
disorder, these alerts are inevitable.
;
_vrf_PLAT602_Avermectin_B1a
;
PROBLEM: Solvent Accessible VOID(S) in the Unit-Cell .... ! Check
RESPONSE: We assigned as many solvent molecules
as possible in the pore.
However, several solvent molecules could not be modeled due to
severe disorder in the large pore of the MOF.
;
_vrf_PLAT934_Avermectin_B1a
;
PROBLEM: Number of (Iobs-Icalc)/Sigma(W) > 10 Outliers .. 11 Check
RESPONSE: In the low-angle region, multiple diffraction
peaks overlapped due to the large unit cell parameters of the MOF.
Furthermore, during omega scan measurements, several low-angle
reflections were obscured by the shadow of the beamline beamstop.
;
_vrf_THETM01_Avermectin_B1a
;
PROBLEM: The value of sine(theta_max)/wavelength is less than 0.575
RESPONSE: A full set of data was collected.
However, the crystal diffracted very weakly in the high angle range
beyond d value of 0.90 angstrom due to the highly porous
structure of the MOF.
;

CCDC 2530578 (3,4-Dihydroivermectin B_{1a}@APF-40)

_vrf_PLAT049_Dihydro_ivermectin_B1a
;
PROBLEM: Calculated Density Less Than 1.0 gcm-3 .... 0.6537 Check
RESPONSE: This alert is related to PLAT602_ALERT_2_A.
In this analysis, the crystal density appears small due to
the presence of voids that should be occupied by solvent molecules.
;
_vrf_PLAT341_Dihydro_ivermectin_B1a
;
PROBLEM: Low Bond Precision on C-C Bonds .... 0.04107 Ang.
RESPONSE: Since the MOF formed the highly porous framework,
the partial structures of the framework that did not form clear
interactions with the guest molecules had severe disorder.
;
_vrf_PLAT430_Dihydro_ivermectin_B1a
;
PROBLEM: Short Inter D...A Contact O3W ..O32B . 2.65 Ang.
RESPONSE: These interactions should be hydrogen bonds.
However, the hydrogen atoms of waters could not be modeled due to
disorder, these alerts are inevitable.
;
_vrf_PLAT602_Dihydro_ivermectin_B1a
;
PROBLEM: Solvent Accessible VOID(S) in the Unit-Cell .... ! Check
RESPONSE: We assigned as many solvent molecules
as possible in the pore.
However, several solvent molecules could not be modeled due to
severe disorder in the large pore of the MOF.
;
_vrf_PLAT934_Dihydro_ivermectin_B1a
;
PROBLEM: Number of (Iobs-Icalc)/Sigma(W) > 10 Outliers .. 8 Check
RESPONSE: In the low-angle region, multiple diffraction
peaks overlapped due to the large unit cell parameters of the MOF.
Furthermore, during omega scan measurements, several low-angle
reflections were obscured by the shadow of the beamline beamstop.
;

_vrf_THETM01_Dihydro_ivermectin_B1a
;
PROBLEM: The value of sine(theta_max)/wavelength is less than 0.550
RESPONSE: A full set of data was collected.
However, the crystal diffracted very weakly in the high angle range
beyond d value of 1.00 angstrom due to the highly porous
structure of the MOF.

;

CCDC 2530579 (Ethyl_ivermectin@APF-40)

_vrf_PLAT049_Ethyl_ivermectin
;
PROBLEM: Calculated Density Less Than 1.0 gcm-3 .... 0.7139 Check
RESPONSE: This alert is related to PLAT602_ALERT_2_A.
In this analysis, the crystal density appears small due to
the presence of voids that should be occupied by solvent molecules.
;
_vrf_PLAT341_Ethyl_ivermectin
;
PROBLEM: Low Bond Precision on C-C Bonds .... 0.06253 Ang.
RESPONSE: Since the MOF formed the highly porous framework,
the partial structures of the framework that did not form clear
interactions with the guest molecules had severe disorder.
;
_vrf_PLAT430_Ethyl_ivermectin
;
PROBLEM: Short Inter D...A Contact O1W ..N4A . 2.68 Ang.
RESPONSE: These interactions should be hydrogen bonds.
However, the hydrogen atoms of waters could not be modeled due to
disorder, these alerts are inevitable.
;
_vrf_PLAT602_Ethyl_ivermectin
;
PROBLEM: Solvent Accessible VOID(S) in the Unit-Cell .... ! Check
RESPONSE: We assigned as many solvent molecules
as possible in the pore.
However, several solvent molecules could not be modeled due to
severe disorder in the large pore of the MOF.
;
_vrf_PLAT934_Ethyl_ivermectin
;
PROBLEM: Number of (Iobs-Icalc)/Sigma(W) > 10 Outliers .. 5 Check
RESPONSE: In the low-angle region, multiple diffraction
peaks overlapped due to the large unit cell parameters of the MOF.
Furthermore, during omega scan measurements, several low-angle
reflections were obscured by the shadow of the beamline beamstop.
;
_vrf_THETM01_Ethyl_ivermectin
;
PROBLEM: The value of sine(theta_max)/wavelength is less than 0.550
RESPONSE: A full set of data was collected.
However, the crystal diffracted very weakly in the high angle range
beyond d value of 1.08 angstrom due to the highly porous

structure of the MOF.
;

9. References.

(1) Kabsch, W. *XDS*. *Acta Crystallogr. D Biol. Crystallogr.* **2010**, *66* (2), 125–132. https://doi.org/10.1107/S0907444909047337.

(2) Sheldrick, G. M. *SHELXT* – Integrated Space-Group and Crystal-Structure Determination. *Acta Crystallogr. Sect. Found. Adv.* **2015**, *71* (1), 3–8. https://doi.org/10.1107/S2053273314026370.

(3) Sheldrick, G. M. Crystal Structure Refinement with *SHELXL*. *Acta Crystallogr. Sect. C Struct. Chem.* **2015**, *71* (1), 3–8. https://doi.org/10.1107/S2053229614024218.

(4) Dolomanov, O. V.; Bourhis, L. J.; Gildea, R. J.; Howard, J. A. K.; Puschmann, H. *OLEX2* : A Complete Structure Solution, Refinement and Analysis Program. *J. Appl. Crystallogr.* **2009**, *42* (2), 339–341. https://doi.org/10.1107/S0021889808042726.

(5) Wu, Y.; Shi, L.; Xu, L.; Ying, J.; Miao, X.; Hua, B.; Chen, Z.; Sessler, J. L.; Huang, F. Supramolecular Docking Structure Determination of Alkyl-Bearing Molecules. *Nature* **2025**, *640* (8059), 676–682. https://doi.org/10.1038/s41586-025-08833-2.

(6) Wada, Y.; Usov, P. M.; Chan, B.; Mukaida, M.; Ohmori, K.; Ando, Y.; Fuwa, H.; Ohtsu, H.; Kawano, M. Atomic-Resolution Structure Analysis inside an Adaptable Porous Framework. *Nat. Commun.* **2024**, *15* (1), 81. https://doi.org/10.1038/s41467-023-44401-w.

(7) Rosenberger, L.; Von Essen, C.; Khutia, A.; Kühn, C.; Georgi, K.; Hirsch, A. K. H.; Hartmann, R. W.; Badolo, L. Crystalline Sponge Affinity Screening: A Fast Tool for Soaking Condition Optimization without the Need of X-Ray Diffraction Analysis. *Eur. J. Pharm. Sci.* **2021**, *164*, 105884. https://doi.org/10.1016/j.ejps.2021.105884.

(8) Pei, X.; Bürgi, H.-B.; Kapustin, E. A.; Liu, Y.; Yaghi, O. M. Coordinative Alignment in the Pores of MOFs for the Structural Determination of N-, S-, and P-Containing Organic Compounds Including Complex Chiral Molecules. *J. Am. Chem. Soc.* **2019**, *141* (47), 18862–18869. https://doi.org/10.1021/jacs.9b10501.

(9) Tu, T. N.; Scheer, M. A Novel Crystalline Template for the Structural Determination of Flexible Chain Compounds of Nanoscale Length. *Chem* **2023**, *9* (1), 227–241. https://doi.org/10.1016/j.chempr.2022.10.016.

(10) Nakagawa, T.; Wada, Y.; Chan, B.; Baba, T.; Hanaya, K.; Koseki, Y.; Asano, R.; Aoki, K.; Usov, P. M.; Kawano, M. Structural Elucidation of Nucleophilic Compounds through Synergistic Coordination and Hydrogen Bonding in a Metal–Organic Framework. *J. Am. Chem. Soc.* **2025**, *147* (32), 29013–29025. https://doi.org/10.1021/jacs.5c07192.

(11) Tashiro, S.; Nakata, K.; Hayashi, R.; Shionoya, M. Multipoint Hydrogen Bonding-Based Molecular Recognition of Amino Acids and Peptide Derivatives in a Porous Metal-Macrocycle Framework: Residue-Specificity, Diastereoselectivity, and Conformational Control. *Small* **2021**, *17* (22), 2005803. https://doi.org/10.1002/smll.202005803.

(12) He, W.; Yu, Y.; Iizuka, K.; Takezawa, H.; Fujita, M. Supramolecular Coordination Cages as Crystalline Sponges through a Symmetry Mismatch Strategy. *Nat. Chem.* **2025**, *17* (5), 653–662. https://doi.org/10.1038/s41557-025-01750-x.

(13) Choi, D.; Lee, H.; Lee, J. J.; Jung, O.-S. Practical Porous Matrix for Molecular Structure Determination of General Liquid Chemicals. *Cryst. Growth Des.* **2017**, *17* (12), 6677–6683. https://doi.org/10.1021/acs.cgd.7b01301.

(14) Mon, M.; Bruno, R.; Ferrando-Soria, J.; Bartella, L.; Di Donna, L.; Talia, M.; Lappano, R.; Maggiolini, M.; Armentano, D.; Pardo, E. Crystallographic Snapshots of Host–Guest Interactions in Drugs@metal–Organic Frameworks: Towards Mimicking Molecular Recognition Processes. *Mater. Horiz.* **2018**, *5* (4), 683–690. https://doi.org/10.1039/C8MH00302E.
(15) Liu, J.-C.; Huang, W.-P.; Tian, Y.-X.; Xu, W.; Ye, W.-C.; Jiang, R.-W. A New Versatile Crystalline Sponge for Organic Structural Analysis without the Need for Activation. *J. Mater. Chem. A* **2024**, *12* (21), 12609–12618. https://doi.org/10.1039/D3TA07946E.
(16) Lunn, R. D. J.; Tocher, D. A.; Sidebottom, P. J.; Montgomery, M. G.; Keates, A. C.; Carmalt, C. J. Encapsulation of Aromatic Compounds and a Non-Aromatic Herbicide into a Gadolinium-Based Metal–Organic Framework via the Crystalline Sponge Method. *Cryst. Growth Des.* **2020**, *20* (11), 7238–7245. https://doi.org/10.1021/acs.cgd.0c00901.
(17) Inokuma, Y.; Matsumura, K.; Yoshioka, S.; Fujita, M. Finding a New Crystalline Sponge from a Crystallographic Database. *Chem. – Asian J.* **2017**, *12* (2), 208–211. https://doi.org/10.1002/asia.201601551.
(18) Balestri, D.; Mazzeo, P. P.; Carraro, C.; Demitri, N.; Pelagatti, P.; Bacchi, A. Stepwise Evolution of Molecular Nanoaggregates Inside the Pores of a Highly Flexible Metal–Organic Framework. *Angew. Chem.* **2019**, *131* (48), 17503–17511. https://doi.org/10.1002/ange.201907621.
(19) Habib, F.; Tocher, D. A.; Carmalt, C. J. Encapsulation of N-Containing Compounds in a New Hydrophilic Cd-Based Crystalline Sponge *via* Coordinative Alignment Method. *CrystEngComm* **2023**, *25* (35), 5001–5011. https://doi.org/10.1039/D3CE00592E.
(20) Yang, X.-Y.; Yuan, S.; Qin, J.-S.; Lollar, C.; Alsalme, A.; Zhou, H.-C. A Flexible Thioether-Based MOF as a Crystalline Sponge for Structural Characterization of Liquid Organic Molecules. *Mater. Chem. Front.* **2017**, *1* (9), 1764–1767. https://doi.org/10.1039/C7QM00152E.
(21) Qiu, Q.; Chen, C.-X.; Wei, Z.-W.; Cao, C.-C.; Zhu, N.-X.; Wang, H.-P.; Wang, D.; Jiang, J.-J.; Su, C.-Y. A Flexible Cu-MOF as Crystalline Sponge for Guests Determination. *Inorg. Chem.* **2019**, *58* (1), 61–64. https://doi.org/10.1021/acs.inorgchem.8b02993.
(22) Tipthara, P.; Kobylinski, K. C.; Godejohann, M.; Hanboonkunupakarn, B.; Roth, A.; Adams, J. H.; White, N. J.; Jittamala, P.; Day, N. P. J.; Tarning, J. Identification of the Metabolites of Ivermectin in Humans. *Pharmacol. Res. Perspect.* **2021**, *9* (1). https://doi.org/10.1002/prp2.712.
(23) Li, S.; Li, Z.; Pang, S.; Xiang, W.; Wang, W. Coordinating Precursor Supply for Pharmaceutical Polyketide Production in Streptomyces. *Curr. Opin. Biotechnol.* **2021**, *69*, 26–34. https://doi.org/10.1016/j.copbio.2020.11.006.
